# Efficient prime editing in two-cell mouse embryos using PEmbryo

**DOI:** 10.1038/s41587-023-02106-x

**Published:** 2024-02-06

**Authors:** Rebecca P. Kim-Yip, Ryan McNulty, Bradley Joyce, Antonio Mollica, Peter J. Chen, Purnima Ravisankar, Benjamin K. Law, David R. Liu, Jared E. Toettcher, Evgueni A. Ivakine, Eszter Posfai, Britt Adamson

**Affiliations:** 1 Department of Molecular Biology, Princeton University, Princeton, NJ USA; 2 Lewis-Sigler Institute for Integrative Genomics, Princeton University, Princeton, NJ USA; 3 Genetics and Genome Biology Program, The Hospital for Sick Children, Toronto, ON Canada; 4 Department of Biochemistry, University of Toronto, Toronto, ON Canada; 5 Merkin Institute of Transformative Technologies in Healthcare, Broad Institute of MIT and Harvard, Cambridge, MA USA; 6 Department of Chemistry and Chemical Biology, Harvard University, Cambridge, MA USA; 7 Howard Hughes Medical Institute, Harvard University, Cambridge, MA USA; 8 Department of Physiology, University of Toronto, Toronto, ON Canada; 9 Department of Molecular Genetics, University of Toronto, Toronto, ON Canada; 10 Present Address: Prime Medicine, Inc., Cambridge, MA USA; 11 Present Address: Immunology and Microbial Pathogenesis Program, Weill Cornell Graduate School of Medical Sciences, New York, NY USA

**Keywords:** Genomics, Genetic techniques, Embryology

## Abstract

Using transient inhibition of DNA mismatch repair during a permissive stage of development, we demonstrate highly efficient prime editing of mouse embryos with few unwanted, local byproducts (average 58% precise edit frequency, 0.5% on-target error frequency across 13 substitution edits at 8 sites), enabling same-generation phenotyping of founders. Whole-genome sequencing reveals that mismatch repair inhibition increases off-target indels at low-complexity regions in the genome without any obvious phenotype in mice.

## Abstract (web summary)

Prime editing is used to efficiently edit early-stage mouse embryos with few on-target byproducts.

## Main

Engineered CRISPR-Cas systems have revolutionized our ability to alter the genomes of mice, greatly enhancing our ability to model genetic diseases and study mammalian development^1,2^. Technical constraints nevertheless persist and continue to limit applications. A particular challenge is that, due to low or inconsistent editing efficiencies^3,4^, unwanted generation of on-target byproducts^4–9^ and/or limited versatility associated with specific approaches^10–13^, studying the effects of specific genetic changes typically requires new mouse lines to be established for each variant of interest. An editing system capable of enabling same-generation phenotyping through flexible, high-efficiency on-target editing would therefore be advantageous. To date, same-generation phenotyping has been demonstrated for generating knockouts and knock-ins by taking advantage of Cas9-induced DNA double-strand breaks and endogenous DNA repair^4,14–16^, albeit with on-target somatic mosaicism that complicates interpretation of phenotype^17,18^, or more recently by using base editors^19,20^. In principle, prime editing offers a way to achieve similar capabilities but with fewer unwanted alterations to the targeted genomic locus and with flexibility in the types of small edits installed^21^. This approach uses reverse transcription to ‘write’ programmed edits into the genome and thus allows many edit types (that is, base substitutions, deletions and small insertions) to be installed with few observed byproducts. Unfortunately, attempts to use prime editing in mouse embryos have, thus far, found low-efficiency precise editing (typically <20% per embryo and often undetectable) or a high frequency of undesired outcomes^22–26^, with results varying across prime editing systems, target sites and studies. Here, by testing enhanced prime editing systems^27^ and deploying editing components during a permissive stage of development, we show how prime editing can be used to efficiently edit mouse embryos and, in a proof-of-principle experiment, achieve same-generation phenotyping.

The simplest form of prime editing is a two-component system requiring only a programmable Cas9 nickase fused to an engineered reverse transcriptase (nCas9-RT) and a prime editing guide RNA (pegRNA) that specifies an edit and genomic target (Supplementary Fig. 1a)^21^. When delivered to cells, these components bind the target DNA, nick the non-complementary strand and release an unbound 3′ DNA flap. This flap can then anneal to the 3′ end of the pegRNA and prime reverse transcription to synthesize the specified edit into the nicked DNA strand. Mechanisms of DNA repair and/or replication then presumably incorporate the edit into the genome^27,28^. The first report of prime editing^21^ showed that the efficiency of this process can be improved by introducing a complementary-strand nick near the pegRNA target sequence with an additional single guide RNA (sgRNA), albeit with a concomitant increase in unintended, on-target outcomes. We and others later discovered that endogenous mechanisms of DNA mismatch repair (MMR) impede prime editing of small edits with or without the complementary-strand nick and promote the formation of unwanted byproducts^27,28^. Guided by this insight, we engineered a dominant negative MMR protein (MLH1dn) that can improve both the efficiency and precision of prime editing in cultured cells^27^. Prime editing without and with the complementary nick is designated PE2 and PE3, respectively; in the presence of MLH1dn, we refer to these systems as PE4 and PE5.

**Fig. 1:**
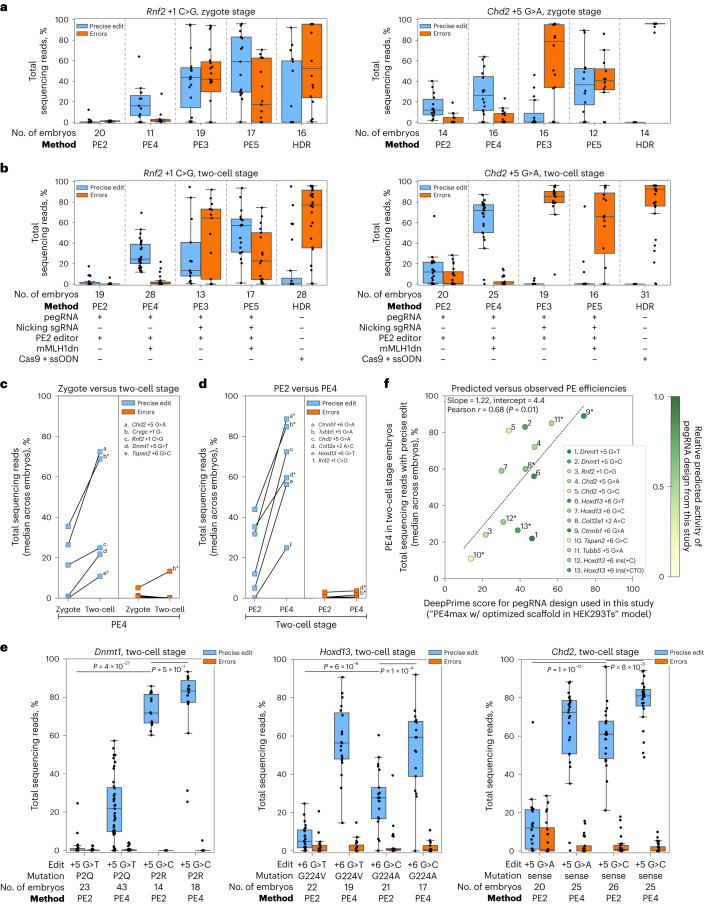
Dominant negative mMLH1 and delivery at the two-cell stage improves prime editing in mouse embryos. **a**, Percentages of total reads containing only the indicated precise edit (blue) or errors (orange) in *Rnf2* (left) or in *Chd2* (right). Each data point represents an individual embryo edited at the zygote stage. Editing conditions indicated in **b**. **b**, Same as **a**, except each data point represents an individual embryo edited at the two-cell stage. HDR, homology-directed repair; ssODN, single-strand oligonucleotide donor. **c**, Median precise edit (blue) and error (orange) frequencies across embryos microinjected with PE4 components (editor mRNA, pegRNA, mMLH1dn mRNA) at the zygote or two-cell stage. Plot includes data also represented in **a**, **b** and **e**. **d**, Same as **c**, except plot represents data from embryos microinjected at the two-cell stage with PE2 components (editor mRNA, pegRNA) or PE4 components (editor mRNA, pegRNA, mMLH1dn mRNA). Plot includes data also represented in **b** and **e**. **e**, Percentages of total reads containing only the indicated precise edit (blue) or errors (orange) from individual embryos microinjected at the two-cell stage. Plots include *Chd2* results from **b**. **f**, Comparison of predicted prime editing efficiencies (DeepPrime score) from a deep-learning-based model^44^ trained on editing results in HEK293T cells using an optimized prime editor (PEmax) and hMLH1dn to observed prime editing (PE) efficiencies in mouse embryos microinjected at the two-cell stage with PE4 components (editor mRNA, pegRNA, mMLH1dn mRNA) from this study. Each dot represents the specific pegRNA design used in our study. Color shade indicates the relative predicted score of the pegRNA compared to the maximum score predicted by the DeepPrime-FT model across all feasible pegRNA designs (Methods) for a given target site/edit. Pearson correlation coefficient (*r* = 0.68) reported with *P* value (*P* = 0.01) from two-sided *t*-test. Dashed line represents fit from linear least-squares regression. Throughout our study, asterisks specify use of the optimized PEmax editor (PE2*, PE4* methods), as opposed to the PE2 editor^21^ (PE2, PE4 methods). Data in **a**–**f** are compiled from multiple experiments (Supplementary Tables 7–9 and Methods). For **c** and **d**, lowercase letters indicate edit and black lines connect results for the same edit across conditions. For **c**–**e**, *P* values are from two-sided Student’s *t*-tests. For box plots, boxes indicate the median and interquartile range (IQR) for each group of embryos with whiskers extending 1.5 × IQR past the upper and lower quartiles.

Applications of prime editing in embryos have reported poor PE2 efficiencies and extensive PE3-generated byproducts^26,29^. Reasoning that suppression of MMR could provide an enhanced strategy in this setting as well, we tested systems of prime editing with and without mouse codon-optimized MLH1dn (mMLH1dn) in embryos (PE2, PE3, PE4 and PE5). For initial experiments, we chose two edits previously shown to support prime editing in mouse cells^26,27^: a + 1 C > G substitution at *Rnf2* that disrupts the protospacer adjacent motif (PAM)-proximal seed region of the target sequence, and a + 5 G > A substitution at *Chd2* that disrupts the PAM directly (Supplementary Fig. 1b,c). We obtained synthesized pegRNAs encoding these edits and microinjected them into the cytoplasm of mouse zygotes with in vitro-transcribed mRNA encoding a version of the nCas9-RT editor (specifically, the prime editor 2 or ‘PE2’ construct^21^), without or with complementary nicking sgRNAs, and without or with mRNA encoding mMLH1dn^27^ (Supplementary Tables 1–3). We cultured zygotes to the blastocyst stage and evaluated editing by amplicon sequencing (Fig. 1a and Supplementary Tables 4–8). Throughout this study, we analyzed amplicon sequencing data by categorizing reads as unmodified (‘WT’), modified with only the programmed edit (‘precise edit’) or modified with any unintended sequence change near the edit site (‘errors’) (Methods, Supplementary Fig. 2a and Supplementary Tables 7–14). When using a complementary-strand nick or comparing to data generated with one, we also evaluated errors around the secondary nick site. Notably, because an error classification could signify either the presence of an editing byproduct or a technical artifact introduced during PCR and/or sequencing (Supplementary Fig. 2a–c), we formally compared samples microinjected with prime editing components to unedited controls (embryos microinjected with only nCas9-RT mRNA or uninjected) when assessing editing outcomes and subtracted the average error rate in the control group (2–6% of reads depending on the target site) from reported values (Methods, Supplementary Figs. 2a–c and 3a,b and Supplementary Table 7). Embryos classified as unedited or devoid of errors may therefore represent either failure of editing or editing below the limit of detection.

Across prime editing systems, we observed markedly different frequencies of editing in zygotes (Fig. 1a and Supplementary Table 8). In PE2-edited embryos, we observed minimal to moderate modification of the edit site (average precise editing: *Rnf2* 1%, *Chd2* 16%; average adjusted errors at target site: *Rnf2* 1%, *Chd2* 3%), with no embryo showing precise editing at high frequencies (>50%). In PE3-edited embryos, we achieved higher frequencies of overall editing but found on-target byproducts to be common (average precise editing: *Rnf2* 42%, *Chd2* 8%; average adjusted errors at target site: *Rnf2* 44%, *Chd2* 60%), consistent with previous reports^17,26,29^. In PE5-edited embryos (microinjected with the same nicking sgRNAs as PE3), we again observed high frequencies of modification but, here too, observed frequent on-target byproducts (average precise editing: *Rnf2* 56%, *Chd2* 39%; average adjusted errors at target site: *Rnf2* 27%, *Chd2* 40%). These byproducts had similar sequence features to those in PE3-edited embryos, including two major types: deletions that remove at least some of the sequence between nick sites and ‘combined’ outcomes with both the intended modification and a 3′ deletion (Supplementary Figs. 4a,b and 5a,b). Given these outcomes, we concluded that, in this setting, neither PE3 nor PE5 hold a major advantage over conventional editing with homology-directed repair (HDR), which also frequently generated unwanted, on-target mutations (Fig. 1a and Supplementary Tables 8 and 15). We did, however, observe that inclusion of mMLH1dn with PE2 in zygotes (PE4) yielded reasonable levels of precise editing at both loci without substantially increasing byproduct formation (average precise editing: *Rnf2* 20%, *Chd2* 28%; average adjusted errors at target site: *Rnf2* 4%, *Chd2* 4%).

Motivated by our previous work showing that installation of large DNA fragments with nuclease-active Cas9 is highly efficient in two-cell-stage embryos^30^, we also tested PE2, PE3, PE4 and PE5 at this stage of development (Fig. 1b, Supplementary Fig. 6a,b and Supplementary Table 8). For these experiments, we used similar microinjection procedures and delivered the same editing components tested in zygotes, except here, we performed individual microinjections into the cytoplasm of each cell of two-cell-stage embryos. We cultured the embryos to the blastocyst stage and sequenced the target site. Similar to zygotes, PE2 in two-cell-stage embryos showed mostly low to moderate levels of programmed editing (average precise editing: *Rnf2* 3%, *Chd2* 14%), whereas PE3 and PE5 generated many on-target byproducts (average adjusted errors at target site: *Rnf2* 47% with PE3 and 28% with PE5, *Chd2* 82% with PE3 and 59% with PE5) (Supplementary Figs. 7a,b and 8a,b). Editing with PE4, though, achieved high levels of programmed editing at one locus (average precise editing, *Chd2* 63%), moderate levels of programmed editing at the other (average precise editing, *Rnf2* 29%) and minimal on-target byproduct formation at both sites (average adjusted errors at target site: *Rnf2* 3%, *Chd2* 3%). We reasoned that this system warranted further investigation.

**Fig. 2:**
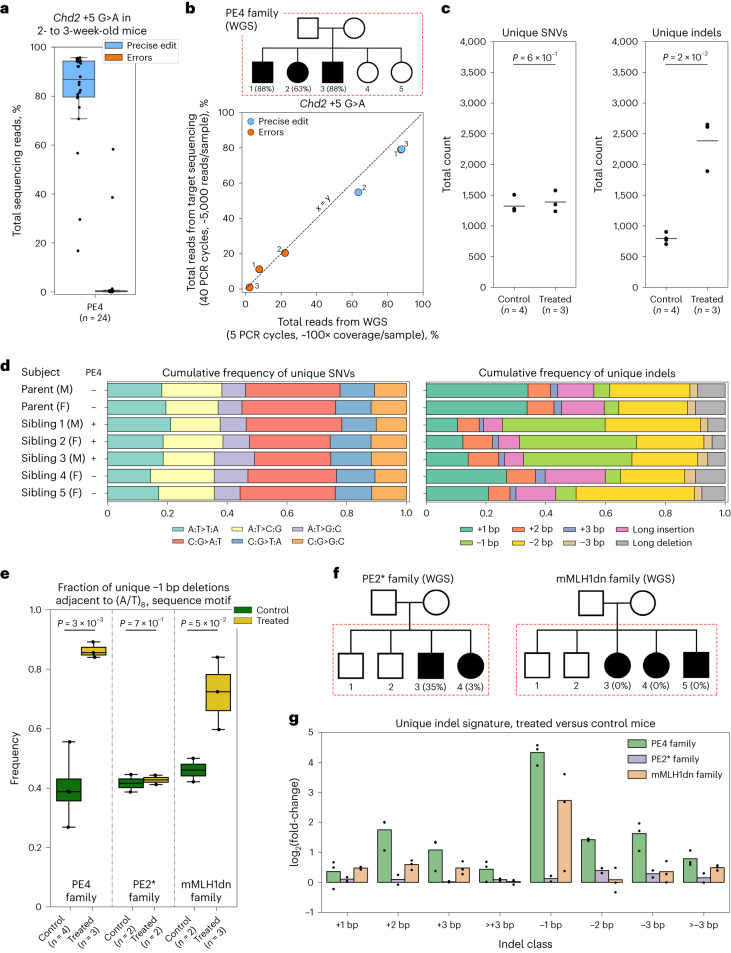
WGS after transient MMR inhibition in embryos. **a**, Percentages of total reads containing the precise +5 G > A edit (blue) or errors (orange) in *Chd2* from ear clips of 2- to 3-week-old mice developed from embryos microinjected with PE4 components (PE2 editor mRNA, pegRNA, mMLH1dn mRNA) at the two-cell stage. Data compiled from multiple experiments (Supplementary Table 13 and Methods). **b**, Pedigree of ‘PE4 family’ (top). Black indicates the ‘treated’ group of select progeny microinjected at the two-cell stage with PE4 components (PE2 editor mRNA, *Chd2* + 5 G > A pegRNA, mMLH1dn mRNA). Unshaded family members indicate mice/embryos treated as the ‘control’ group, including sibling progeny microinjected at the two-cell stage with PE2 editor mRNA only. Percentages indicate precise edit efficiency at E12.5 as determined by WGS. Plot (bottom) compares editing frequencies in treated embryos across sequencing methods (target versus whole-genome sequencing). Superscripts denote individual progeny from ‘PE4 family’. Dashed line represents *x* = *y*. **c**, Total unique SNVs (left) and total unique indels (right) detected in members of the ‘PE4 family’ after joint genotyping (black line indicates mean from each group, *P* values from two-sided Welch’s *t*-tests). **d**, Cumulative frequencies of unique SNVs (left) or unique indels (right) by type for members of the ‘PE4 family’. F, female; M, male. **e**, Fraction of unique −1 bp deletions directly adjacent to poly(A/T) nucleotide tracts in treated and control mice/embryos from each indicated family (*P* values from two-sided Welch’s *t*-test). Treated and control groups are defined in **b** and **f**. **f**, Pedigrees of additional mouse families. Black denotes treated groups. Unshaded siblings comprise control groups. For the ‘PE2* family’ (left), treated embryos were microinjected with PE2* components (PEmax mRNA, *Chd2* + 5 G > A pegRNA) at the two-cell stage, whereas control embryos were microinjected with pegRNA only. For the ‘mMLH1dn family’ (right), treated embryos were microinjected with mMLH1dn mRNA and the *Chd2* + 5 G > A pegRNA (but no editor) at the two-cell stage, whereas control embryos were microinjected with pegRNA only. One control embryo (not indicated) was omitted from analysis for this family after sequencing failed quality control (Methods). Percentages indicate precise edit efficiency in treated embryos at E12.5. **g**, Number of unique indels detected in treated embryos in each family (*n* = 3, PE4 family; *n* = 2, PE2* family; *n* = 3, mMLH1dn family) relative to the average of control mice/embryos from the same family. Data points represent fold-change for individual mice/embryos. Bars indicate the mean difference. For pedigree diagrams, red dashed boxes indicate mice/embryos subjected to WGS. For box plots, boxes indicate the median and IQR of each group with whiskers extending 1.5 × IQR (**a**) and 2 × IQR (**e**) past the upper and lower quartiles.

To further test PE4-based editing in two-cell-stage embryos, we selected additional edits across seven different target loci: +2 A > C in *Col12a1* (ref. ^26^), +5 G > T in *Dnmt1* (ref. ^21^), +5 G > A in *Tubb5* (ref. ^31^), +6 G > C in *Tspan2* (ref. ^25^), +6 G > A in *Ctnnb1* (ref. ^32^), +6 G > T in *Hoxd13* (ref. ^23^) and deletion of +1 G in *Crygc*^24^. Using pegRNAs specifying these edits, we evaluated each component of the approach: (1) editing in two-cell-stage embryos (Fig. 1c) and (2) editing with mMLH1dn (Fig. 1d, Supplementary Fig. 9a,b and Supplementary Table 9). Comparison of PE4 editing between zygotes and two-cell-stage embryos confirmed that microinjection at the two-cell stage produced higher rates of precise edit installation with low rates of errors (*P* < 0.05 for 3 of 5 edits, two-sided Student’s *t*-test), using either the standard PE2 editor or an optimized editor called PEmax (denoted by and asterisk) that we confirmed is compatible with PEmbryo when testing recent advances^27,33^ (Supplementary Fig. 10a–d and Supplementary Table 10). Comparison of editing with and without mMLH1dn in two-cell-stage embryos similarly revealed that PE4 outperforms PE2, achieving higher rates of precise editing with low rates of errors (*P* < 0.05 for 6 of 6 edits, two-sided Student’s *t*-test). Notably, most of these edits were previously tested in zygotes and demonstrated only low-to-intermediate installation rates (although comparisons with published data are difficult due to differences in conditions and quantification). Given these promising results, we termed this approach (PE4 at the two-cell stage) PEmbryo.

Canonical substrates of the mammalian MMR machinery include single base mispairs and small extrahelical loops of ∼1–10 nt (refs. ^34–36^); however, such structures are not all repaired with equal efficiency. C⋅C mismatches, for example, are poor MMR substrates^37–39^, and G > C prime edits, which should form C⋅C mispair intermediates, are accordingly less sensitive to MLH1dn in cultured cells. G > C edits also tend to have higher installation frequencies in the presence of MMR, suggesting that they ‘evade’ suppression by MMR^27,40^. To test the idea of MMR evasion in embryos, we modified three of our pegRNAs (*Dnmt1* + 5 G > T, *Hoxd13* + 6 G > T, *Chd2* + 5 G > A) so that each would encode a G > C substitution. PE2-based editing with these pegRNAs showed that all three G > C substitutions were installed at a higher frequency than G > T or G > A in the same positions (increases in average precise editing of 38-fold for *Dnmt1*, 3.9-fold for *Hoxd13* and 4.4-fold for *Chd2*), albeit with some remaining sensitivity to MMR, as demonstrated by further increases from inclusion of mMLH1dn (average precise editing with PEmbryo: *Dnmt1* 77%, *Hoxd13* 53% and *Chd2* 78%) (Fig. 1e and Supplementary Table 9). Overall, these results suggest that the more an edit is shielded from MMR, the more efficiently it will be installed.

Notably, other prime edit types have also been suggested to evade MMR, including ones designed to generate heteroduplex intermediates with three to five contiguous mispairs^27^. Evaluation of such edits in embryos, however, revealed MMR responsiveness (Supplementary Fig. 11a,b and Supplementary Table 11), suggesting that rules for MMR evasion are likely to be complex. Nevertheless, results from testing these edits demonstrated that installation of other edit types are improved by PEmbryo. To evaluate whether even larger edit types could be installed, we designed a series of *Hoxd13* pegRNAs encoding 1-, 3-, 8- and 17-nt insertions^41^, keeping the primer binding site and the 3′ homology arm of the RT template constant (Supplementary Fig. 12a). Although we observed successful installation of these insertions, rates were lower than we had observed earlier with matched substitution edits (G > T, G > C), with a greater fraction of embryos containing errors and a decrease in efficiency as insert length increased (average precise editing: 1-bp insertion 34%, 3-bp insertion 31%, 8-bp insertion 12%, 17-bp insertion: 2%; average adjusted errors 5–10%) (Supplementary Fig. 12b and Supplementary Table 12).

Given observation of high rates of precise editing at several of our targets (*Ctnnb1* + 6 G > A, *Tubb5* + 5 G > A, *Dnmt1* + 5 G > C, *Chd2* + 5 G > C, deletion of +1 G in *Crygc*) (Fig. 1c,d and Supplementary Fig. 9a,b), we next asked if there exists a method for identifying high-efficiency targets. Recently, deep-learning models have been developed to predict prime editing efficiency across target sites, edits and pegRNA designs (Supplementary Fig. 13a)^41–44^. Using our PE4 results (median frequency of precise editing per group) from microinjecting two-cell-stage mouse embryos for all applicable edits (*n* = 13), we compared editing efficiencies at these sites to predictions from the DeepPrime-FT model^44^ trained on editing results in HEK293T cells using the PE4max approach (PE4 method with the PEmax editor, denoted PE4* in our study). Comparison of model scores based on our specific pegRNA designs revealed significant correlation (Pearson *r* = 0.68, *P* = 0.01, two-sided Student’s *t*-test; Fig. 1f) and, when restricting analysis to only edits made with PEmax (*n* = 6), our results strongly matched model predictions with striking correlation (Pearson *r* = 0.93, *P* = 0.006; Supplementary Fig. 13b). Furthermore, model predictions suggested that for several target sites/edits, our pegRNA design could be optimized to further increase editing efficiencies (Fig. 1f, Supplementary Fig. 13b,c and Supplementary Table 16). These findings demonstrate a means of using computational design to optimize prime editing in mouse embryos.

Our promising results in embryos motivated us to ask if PEmbryo could be used to genetically engineer mice. Given that mice deficient for MMR (for example, mutations in *Mlh1* and *Pms2*) are infertile^45–47^, an immediate concern was that transient expression of mMLH1dn could impact fertility. We therefore microinjected mMLH1dn mRNA into two-cell-stage embryos, transferred these embryos into pseudopregnant females and monitored the viability and fertility of resulting offspring. We found that mMLH1dn had no obvious effect on pup numbers (31 pups born from 48 embryos compared to 30 from 55 control embryos) (Supplementary Table 17). Additionally, crossing male and female offspring (two each) to wild-type mice produced litter sizes typical of the CD1 strain used (14, 17, 8 and 13 pups). mMLH1dn therefore did not interfere with the generation of viable and fertile mice. Next, to genetically engineer mice with PEmbryo, we microinjected two-cell-stage embryos with PE4 components including the *Chd2* + 5 G > A pegRNA, which encodes a silent mutation. Manipulated embryos were transferred into pseudopregnant females (67 embryos), genomic DNA (gDNA) was obtained from resulting pups (*n* = 24) and editing at the target site was evaluated by amplicon sequencing. Similar to observations from blastocysts (Fig. 1b), we observed high frequencies of precise editing and low frequencies of byproduct generation (average precise editing: 81%; average adjusted errors at target site: 4%) (Fig. 2a, Supplementary Fig. 14 and Supplementary Table 13). As with microinjection of mMLH1dn mRNA alone, we did not observe fertility or viability changes in *Chd2*-edited mice or any other obvious phenotype (Supplementary Table 18). A second concern was that editing may not be applicable to mice of different genetic backgrounds. In addition to the CD1 embryos used for the majority of this study, we therefore also edited C57Bl/6J embryos with the +1 C > G substitution at *Rnf2* with PEmbryo (Supplementary Fig. 15 and Supplementary Table 14). From this experiment, we observed similar editing frequencies between the two strains (average precise editing: CD1 30%, C57Bl/6J 25%, *P* = 0.3, two-sided Student’s *t*-test).

Genomic instability caused by genetic disruption of MMR has been well established in cultured cell lines^48–50^, various cancers^51–53^ and mice^47,54^, but the genotypic effects of transient MMR suppression have not been well studied. To comprehensively evaluate the impact of transient mMLH1dn expression on the mouse genome, we performed a family-based genetic analysis of PEmbryo-edited embryos (Fig. 2b). Briefly, we edited C57BL/6 J embryos with the *Chd2* + 5 G > A substitution, collected genomic DNA at embryonic day 12.5 (E12.5) and performed whole-genome sequencing (WGS) of these embryos (designated the treated group; shaded subjects in Fig. 2b pedigree) along with unedited sibling embryos microinjected with nCas9-RT mRNA only and both parents (control group; unshaded subjects in Fig. 2b pedigree). Average sequencing depth ranged from 100× to 140× across samples. As above, we chose the *Chd2* + 5 G > A edit because the mutation installed does not disrupt the *Chd2* amino acid sequence. This edit is therefore not expected to result in any confounding phenotypes. Analysis of the *Chd2* locus in PEmbryo-edited family members (*n* = 3) again revealed high rates of precise editing (63–88%) and low-to-moderate rates of target site errors (0–22%), with similar editing frequencies obtained from whole-genome and targeted sequencing (Fig. 2b). Given successful on-target editing, we evaluated changes to the rest of the genome. Consistent with targeted evaluation of MLH1dn in cell lines^27^ and heterozygous *Mlh1*^+/−^ mice^55,56^, microsatellite regions from PEmbryo-edited mice showed no obvious increase in variation (Supplementary Fig. 16 and Supplementary Table 19). Hypothesizing that disruption of MMR machinery would lead to the accumulation of sporadic, medium-to-low frequency alleles as a result of unfixed errors introduced during DNA replication early in development, we looked more globally at the total number of single-nucleotide variants (SNVs) and insertion/deletion events (indels) unique to each family member. Although we observed no significant differences in the number or type of unique SNVs detected between treated (edited embryos) and control groups (sibling embryos and parents), we did detect a 2.5-fold increase in the number of unique indels present in the PEmbryo-edited embryos (*P* = 0.02, two-sided Welch’s *t*-test; Fig. 2c,d). This was primarily driven by an increase in short (1–2 bp) deletions adjacent to regions of high-sequence repetitiveness such as mono- and dinucleotide tracts throughout the genome, consistent with mutational signatures previously observed in nullizygous, *Mlh1*^−/−^ mice^46,54,57,58^ (Fig. 2d,e and Supplementary Figs. 17 and 18).

To confirm that the observed increase in indels was the result of mMLH1dn, we repeated family-based WGS analysis in a pedigree of mice in which select progeny were microinjected at the two-cell stage with mMLH1dn mRNA and the *Chd2* + 5 G > A pegRNA but without any editor (‘mMLH1dn family’), as well as a pedigree in which select progeny were microinjected with PEmax mRNA and the *Chd2* + 5 G > A pegRNA but without mMLH1dn (‘PE2* family’) (Fig. 2f). Once more, we observed no changes in the total number or types of unique SNVs (Supplementary Fig. 19a–c), but two of three mMLH1dn-injected embryos recapitulated the strong increase in −1 bp deletions near mononucleotide tracts observed in the PEmbryo-edited embryos (Fig. 2e–g and Supplementary Figs. 20a–c and 21a–c). Notably, similar to PEmbryo-edited embryos, other classes of deletions were also observed to increase in embryos from ‘mMLH1dn’ and ‘PE2* families’; however, the causal component of these increases could not be well distinguished due to low sample size and technical variation across experimental families (Fig. 2f,g and Supplementary Figs. 20a–c and 21a–c). In summary, we find that, with or without prime editing, transient disruption to MMR early in development promotes genetic instability but with no detectable phenotypic consequences in our study.

**Fig. 3:**
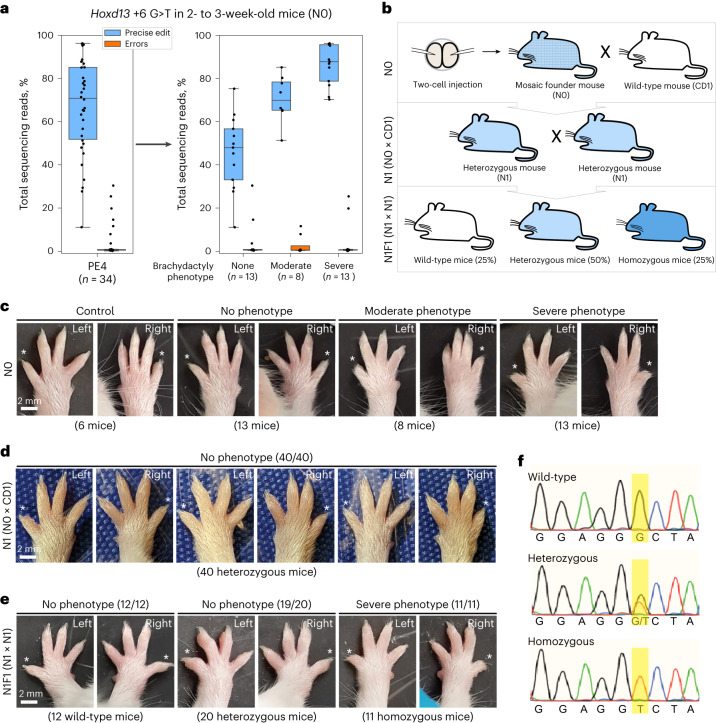
Prime editing with PEmbryo allows for same-generation phenotyping of a substitution edit in mice. **a**, Percentages of total reads containing the precise +6 G > T edit (blue) or errors (orange) in *Hoxd13* from ear clips of 34 pups developed from embryos microinjected with PE4 components (PE2 editor mRNA, pegRNA and mMLH1dn mRNA) at the two-cell stage. Plot on the right depicts the same data as shown on the left, but with mice sorted into three groups based on the severity of the brachydactyly phenotype of the fifth digit on the front limbs (none, moderate or severe). Boxes indicate the median and IQR of each group with whiskers extending 1.5 × IQR past the upper and lower quartiles. **b**, Schematic for breeding of N0 founder mice to generate N1 and N1F1 generations with different genotypes for *Hoxd13* + 6 G > T (G224V). Checkered texture indicates mosaic pattern of edits in the founder mouse. Light blue shading represents heterozygous mice with one copy of the edit. Dark blue shading represents homozygous mice with two copies of the edit. Percentages of the N1F1 generation indicate the expected (not actual) mendelian frequencies of each genotype. **c**, Representative images of left and right front paws of pups from *Hoxd13-*edited N0 founder mice sorted by phenotype severity before sequencing. Control images from comparably aged (18-day-old), *Chd2*-edited pups from the same microinjection experiment. Asterisks indicate fifth digits. **d**, Representative images of left and right front paws of pups from N1 mice heterozygous for *Hoxd13* + 6 G > T (G224V). **e**, Representative images of left and right front paws of pups from N1F1 mice sorted by genotype: wild-type, heterozygous or homozygous for *Hoxd13* + 6 G > T (G224V). **f**, Sanger sequencing traces for N1F1 mice wild-type, heterozygous or homozygous for *Hoxd13* + 6 G > T (G224V). Yellow shading indicates the target site. Trace colors correspond to the base call at each site: T, thymine (red), C, cytosine (blue), A, adenine (green), G, guanine (black).

Encouraged by the efficient and precise on-target editing rates observed with PEmbryo and the lack of observed phenotypes associated with off-target effects, we next asked whether the approach could allow same-generation phenotyping of substitution edits, without the need to establish genetically engineered mouse lines. For this experiment, we targeted the +6 G > T substitution in the *Hoxd13* gene, which generates a single amino acid change (G224V) in the encoded protein. Because a number of mutations in *Hoxd13* have been associated with digit abnormalities in humans and mice, such as syndactyly (fused digits) and brachydactyly (short digits)^59–62^ and with male-specific sterility^63,64^, this edit allowed an opportunity for phenotyping. Similar to our results from sequencing blastocyst embryos, PEmbryo-edited pups (68 two-cell-stage embryos edited and transferred, 34 pups born and analyzed), harbored high frequencies of the precise edit and low frequencies of target site errors (average precise editing: 67%; average adjusted errors: 4%) (Fig. 3a and Supplementary Table 13). Encouragingly, phenotyping these pups revealed that 21 out of 34 *Hoxd13*-edited pups displayed shortened fifth digits on their front limbs (Fig. 3b,c), and further categorizing these brachydactyly phenotypes into ‘moderate’ and ‘severe’ showed that the efficiency of precise editing correlated with phenotype severity (Fig. 3a and Supplementary Fig. 22). Because the majority of the mice categorized as ‘moderate’ and ‘severe’ were among those with no evidence of unwanted mutations at the target site, these data show that PEmbryo is applicable for rapid, same-generation phenotyping of genetic variants in mice.

Next, to determine whether the HOXD13 G224V phenotype was recessive or dominant, we crossed founder mice (N0) containing the *Hoxd13* + 6 G > T edit to wild-type mice and obtained heterozygous N1 animals (Fig. 3b). Indicating the brachydactyly phenotype is recessive, heterozygous mice had normal digits on their front limbs (Fig. 3d). Additionally, when two heterozygous mice were crossed to produce the N1F1 generation, the phenotype reappeared in homozygous N1F1 progeny (11/11 mice), but not wild-type (12/12 mice) or heterozygous (19/20 mice) littermates (Fig. 3e,f). From these crosses, we also found that five out of six male N0 mice with >65% precise editing were infertile, whereas two N0 males with precise editing rates of ∼30–35%, as well as highly edited female mice, produced offspring (Supplementary Table 18). Assaying the fertility of heterozygous and homozygous males (N1 males generated by crossing N0 females with the *Hoxd13* + 6 G > T edit to wild-type mice and N1F1 males generated by crossing N1 heterozygous mice, respectively) revealed that this male-specific fertility phenotype was also recessive (Supplementary Table 20). These results demonstrate that PEmbryo is a powerful method for phenotyping even recessive mutations in the founder generation and will also likely enable same-generation characterization of phenotypes with low penetrance.

Although the impact of MMR on prime editing is now well appreciated in cultured cells^27,28,41^, how this form of DNA repair would affect applications in embryos remained an open question. Here, we show that, when deployed with a complementary-strand nick (PE3), prime editing generates unwanted on-target outcomes in embryos, even when MMR is suppressed (PE5). This observation suggests that prime editing intermediates with adjacent nicks are inherently prone to mutagenic processing in embryos, possibly due to the formation of double-strand breaks. Strategies to avoid such intermediates (for example, by introducing a complementary-strand nick only after resolution of the reverse transcribed strand with PE3b^21^ or PE5b^27^ approaches) may therefore prove useful in embryos^26^; however, such strategies are constrained in their scope of targets by sequence requirements. Alternatively, we show that by using an engineered MMR inhibitor and editing during the two-cell stage of development (PEmbryo), we can achieve high installation efficiencies of small programmed edits (primarily substitutions) without the use of complementary-strand nicks. We thus establish MMR as a major block to installation of small prime edits in embryos and simultaneously provide an approach for overcoming this limitation. Moreover, we demonstrate that small prime edits known to evade detection by MMR (G > C) have higher rates of prime editing with and without mMLH1dn, making these edits particularly attractive in this setting.

Intriguingly, for several edits, we observed higher frequencies of precise editing in embryos than typical with transient delivery of mMLH1dn in cultured cells^27^. Although several factors that differed between studies could explain this observation, cells may also be particularly amenable to prime editing in early development. From a technical perspective, directly microinjecting mRNA-encoded editing components in two-cell-stage embryos may simply allow these components to be present at higher levels and throughout more cell cycles than in other settings. Indeed, although the zygote and two-cell stage in mouse embryos last ∼18–20 h each, subsequent cell cycles are only ∼10–12 h (ref. ^65^), and because PEmbryo exhibits low error frequencies, each new cell cycle should provide new, editable templates, which could account for high editing efficiency. Consistent with this idea, frequencies of PE2 editing have been shown to increase over time in cultured cells when editing components are stably expressed or continually reintroduced^66^.

Irrespective of the underlying mechanism, using PEmbryo, we achieved frequencies of precise on-target editing high enough to establish engineered mouse lines from a single-digit number of embryos and, by producing litters of founder animals largely devoid of small, unwanted, on-target mutations, demonstrated same-generation phenotyping without the need to establish genetically engineered mouse lines. Further, although genome-scale evaluation of off-targets revealed that temporary inhibition of MMR promotes small deletions at repetitive sequence regions throughout the genome—thus providing a note of caution for applications of PE4 and PE5 where such off-target effects may be intolerable—we found that PEmbryo did not result in obvious phenotypic changes nor viability issues in mice, and thus do not preclude use of the technique for modeling purposes. Indeed, PEmbryo may be best suited to rapid phenotyping of many variants, where a causative allele is uncertain or several candidates are of interest, possibly in advance of building outcrossed lines of a few high-priority edits. Additionally, PEmbryo may be well-suited to introducing multiple edits on the same allele, which is challenging due to the formation of large deletions^15,67^, and to editing within essential genes, where unwanted gene disruption may cause embryonic lethality.

Moving forward, independent improvements to prime editing may also enhance PEmbryo. We demonstrated that a recently developed computational model (DeepPrime-FT^44^) trained to predict prime editing efficiencies when inhibiting MMR correlated well with the efficiencies we observed in embryos. Therefore, although our study relied on previously validated pegRNA sequences and target sites^21,23–27,31^, computational tools for predicting active pegRNAs designs and edit efficiencies from sequence context should reduce the need for laborious prescreening^41–44^. With such advances in mind, we highlight that our work not only represents an important step in pinpointing optimized conditions for using prime editing in embryos now but also serves as a foundation for implementing future improvements.

## Methods

### RNA synthesis

We obtained chemically synthesized pegRNAs and sgRNAs from Synthego or Integrated DNA Technologies with chemical modifications at the 5′ and 3′ termini (that is, 2′-O-methyl modified bases and 3′ phosphorothioate linkages) (Supplementary Tables 1, 2 and 15). All standard pegRNAs were quality controlled by Integrated DNA Technologies (IDT). Enhanced pegRNAs (epegRNAs) were too long for quality assurance per IDT. Both pegRNAs and epegRNAs were evaluated in-house using a nanodrop spectrophotometer and observed to range between 24 and 38 pmol μl^−1^, except the pegRNA encoding the 17-nt insertion target to *Hoxd13*, which was 14 pmol μl^−1^. Concentrations were not adjusted from manufacturer’s quality-controlled amounts before microinjection. pegRNA designs were obtained from previous studies and modified for this work^21,23–27,31^. sgRNAs sequences were obtained from previous studies^26,27^ or designed using CRISPOR^68^ (Supplementary Tables 1, 2 and 15).

mRNA for the PE2 editor, PEmax editor and mMLH1dn (Supplementary Table 3) were synthesized as described previously^27^. Briefly, genes were amplified from the corresponding plasmid templates (Addgene, 132775, 178113 and 174825, respectively) using IDT PAGE-purified forward and reverse primers encoding the T7 promoter and a 119-nt poly(A) tail, respectively. Resulting amplicons were column purified and used as the template for RNA synthesis by in vitro transcription (IVT) using HiScribe T7 (NEB, E2040S), Clean Cap AG (TriLink, N-7113) and N1-methylpseudouridine-5′-triphosphate (TriLink, N-1081) reagents. Compared to the mouse MLH1 protein, mMLH1dn lacks the three C-terminal amino acids (ERC). mRNA for Cas9 was synthesized as described previously^30^. Briefly, the IVT template pCS2-Cas9 (Addgene, 122948) was digested using *Not*I enzyme and used as the template for IVT using mMessage mMachine Sp6 Transcription Kit (Thermo Fisher Scientific, AM1340).

One of the baseline nCas9-RT editors used in this study was designed and designated ‘PE2’ by Anzalone and colleagues^21^; we refer to this editor as the PE2 editor or PE2 nCas9-RT throughout our study to distinguish from the PE2 method. This PE2 editor includes the H840A variant of *Streptococcus pyogenes* Cas9 fused through a (SGGS)x2-XTEN16-(SGGS)x2 linker to a variant of the Moloney murine leukemia virus reverse transcriptase (RT) that includes the mutations D200N, T306K, W313F, T330P and L603W. We also used an optimized editor designed and designated ‘PEmax’ by Chen and colleagues^27^, which is PE2 further engineered with a human codon-optimized RT, a 34-aa linker containing a bipartite SV40 NLS, an additional C-terminal c-Myc NLS and R221K N394K mutations in SpCas9. We referred to this optimized nCas9-RT editor as PEmax, the PEmax editor or PEmax nCasRT.

### Oligonucleotides

Single-strand DNA oligonucleotides used for HDR were synthesized and PAGE-purified by either Millipore Sigma or IDT (Supplementary Table 15). DNA oligonucleotides used for PCR were synthesized by IDT and purified by standard desalting (Supplementary Tables 4–6).

### Cytoplasmic microinjection of zygotes and two-cell-stage embryos

Cytoplasmic microinjection of zygotes and two-cell-stage embryos was performed as described previously^30,69^. Briefly, zygotes and two-cell-stage embryos for microinjection were collected at 20 h and 44 h after hCG injection, respectively. Microinjection was performed using an inverted Leica microscope (Dmi8) and mechanical micromanipulators (Leica Microsystems), assisted by the micro-ePore system (World Precision Instruments). Injection pressure was provided by a FemtoJet (Eppendorf). Microinjections were performed in M2 medium (CytoSpring, M2115) in an open glass chamber. Microinjection mixes were prepared in 15 μl total nuclease-free injection buffer (10 mM Tris-HCl, pH 7.4, and 0.25 mM EDTA) as follows: PE2, PE2 nCas9-RT mRNA (100 ng μl^−1^) and pegRNA (75 pmol); PE3, PE2 nCas9-RT mRNA (100 ng μl^−1^), pegRNA (75 pmol) and sgRNA (50 pmol); PE4, PE2 nCas9-RT mRNA (100 ng μl^−1^), pegRNA (75 pmol) and mMlh1dn mRNA (200 ng μl^−1^); PE5, PE2 nCas9-RT mRNA (100 ng μl^−1^), pegRNA (75 pmol), sgRNA (50 pmol) and mMlh1dn mRNA (200 ng μl^−1^); HDR, Cas9 mRNA (100 ng μl^−1^), sgRNA (100 pmol) and donor ssODN (30 ng μl^−1^). For PE2* and PE4* methods, PEMax samples replaced the PE2 nCas9-RT mRNA (100 ng μl^−1^) with PEmax nCas9-RT mRNA (100 ng μl^−1^). epegRNA samples replaced pegRNA (75 pmol) with epegRNA (75 pmol). Microinjection mixes were filtered through Corning Costar Spin-X centrifuge tube-filters (Millipore Sigma, CLS8162) before use. The number of embryos injected in a given experiment was limited by both biological (for example, litter size) and technical (for example, the number of embryos which could be microinjected by a single technician within a reasonable time period) constraints. Therefore, datasets were generated across many individual experiments that each took place over several days. Results reported in the text are therefore an agglomeration of data from individual experiments, with comparisons often made using results from multiple, different experiments. Annotations for each individual embryos, including date of processing, are included in Supplementary Tables 7–14. These tables are organized to include data associated with specific figure panels and thus treatment groups used for multiple comparisons may appear in multiple tables. For repeated treatments groups (that is, specific prime editor, pegRNA design, target site, edit and stage of microinjection), editing rates were consistent across separate experiments.

### Mouse lines and embryos

Mice were housed in an Association for Assessment and Accreditation of Laboratory Animal Care International-accredited facility following the *Guide for the Care and Use of Laboratory Animals*. Animal maintenance and husbandry followed the laboratory Animal Welfare Act. Princeton University’s Institutional Animal Care and Use Committee approved all animal procedures (IACUC protocol number 2133). Mice were subjected to a daily light cycle of 14 h, with an ambient temperature of 21 °C and average ambient humidity of 48%. CD1 (ICR) and C57Bl/6J mouse lines purchased from Charles River Laboratories were used as embryo donors in this study. Embryos were collected from superovulated and mated females in M2 media^30,69^. Zygotes were isolated from the oviduct at E0.5 and washed clean of cumulus cells through a brief treatment with 300 μg ml^−1^ hyaluronidase (Sigma). Two-cell embryos were collected at E1.5. After microinjections, embryos were either immediately transferred into pseudopregnant females or cultured in small drops of EmbryoMax Advanced KSOM Embryo Medium (Millipore Sigma) under paraffin oil (LifeGlobal) at 37 °C, with 5% O_2_ and 6% CO_2_, until they reached the blastocyst stage (E4.5). To generate post-implantation embryos or live pups from microinjected embryos, 25–30 embryos were surgically transferred into the oviducts of CD1 pseudopregnant females immediately after microinjection.

### Embryo or tissue collection and gDNA extraction

Embryo collection and gDNA extraction were performed as described previously^30,69^. Briefly, individual embryos were collected at the blastocyst stage into 4 μl extraction buffer and 1 μl tissue preparation buffer from the Red Extract-N-Amp kit (Sigma XNAT-100RXN). After 15 min of lysis at room temperature, samples were heat-inactivated at 95 °C for 5 min and cooled to room temperature, and 4 μl neutralization buffer was added. DNA was stored at 4 °C until use, for no more than 3 days before subsequent PCR steps. For 2-week-old mice, ear clips were collected from individual animals and then lysed in 40 μl extraction buffer and 10 μl tissue preparation buffer from the Red Extract-N-Amp kit. After 15 min of lysis at room temperature, samples were heat-inactivated at 95 °C for 5 min and cooled to room temperature, and 40 μl neutralization buffer was added. Ear clip preps were stored at 4 °C and processed within 3 days.

### Amplicon library preparation and sequencing

gDNA was collected from edited embryos or mice (ear clip samples), and the target region was amplified in three rounds of PCR before sequencing. Briefly, an initial PCR step (PCR1) amplified our sequence of interest. For *Rnf2*- and *Chd2*-targeted embryos, each 71-μl reaction was composed of 45 pmol each primer, all 9 μl gDNA, 36 μl Phire Hot Start II polymerase master mix (Thermo Fisher Scientific) and molecular-grade water. PCR1 was performed with the following thermocycling conditions: 98 °C for 30 s, 35 cycles of 98 °C for 10 s, 60 °C for 10 s and 72 °C for 30 s, followed by 72 °C for 4 min. For all other target sites/edits, 90-μl PCR1 reactions were composed of 45 pmol (each) forward and reverse primers, all 9 μl gDNA, 45 μl Phire Hot Start II polymerase master mix and molecular-grade water. PCR1 was performed with the following thermocycling conditions: 98 °C for 30 s, 35 cycles of 98 °C for 10 s, 60 °C for 20 s and 72 °C for 1 min and 30 s, followed by 72 °C for 4 min. For genotyping of mice, PCR1 was composed of 2 μl gDNA, 5 pmol of each primer, 8 μl Phire Hot Start II polymerase master mix (*Chd2*) or 10 μl polymerase master mix (*Hoxd13*) and molecular-grade water. PCR1 was performed with the corresponding thermocycler conditions detailed above.

Following PCR1, 2 μl PCR1 product was transferred to a second PCR reaction (PCR2, 20 μl total volume) to add flanking regions that included a 4-bp custom R1 index using 10 pmol each primer with 1x NEBNext Ultra II Q5 master mix (NEB, M0544L) with cycle conditions: 98 °C for 30 s, 8 cycles of 98 °C for 10 s, 64 °C for 20 s and 72 °C for 20 s, followed by 72 °C for 2 min. Finally, 2 μl PCR2 product was transferred to a third PCR reaction (PCR3, 20 μl total volume) to add final Illumina adapters using 10 pmol each primer with 1x NEBNext Ultra II Q5 master mix with cycle conditions: 98 °C for 30 s, 7 cycles of 98 °C for 10 s, 60 °C for 30 s and 72 °C for 30 s, followed by 72 °C for 2 min. Amplicons were pooled and purified by SPRIselect beads (Beckman Coulter, B23318) and then sequenced on an Illumina MiSeq using 300 cycles R1, 8 cycles i7 and 8 cycles i5. Primer sequences for all three PCR steps are provided in Supplementary Tables 4–6.

### Analysis of prime editing efficiency

Dual-indexed target site libraries were sequenced on an Illumina Miseq with 300 cycle V2 reagent kits using a Nano (1M reads) or Micro (4M reads) flow cell depending on the run. Sequence structure was 300 × 8 × 8 × 0 (R1, I7, I5, R2, respectively). Typically, 10–30% phi-X was included to increase base diversity. To differentiate between samples, 4-bp custom barcodes were included in the PCR2 forward primer, resulting in a total barcode length of 20 nt. Libraries were prepared using 96-well plates and pooled volumetrically for sequencing. Typically, we observed ∼5–10% of samples drop out between microinjection experiments and final sequencing results, primarily as a result of failing PCR1 (observed by gel electrophoresis). No obvious differences in dropout rates were observed between groups of edits or indexing primer combinations. Targeted sequencing depth was 10,000 reads per sample.

After sequencing, output base call (BCL) files were converted to FASTQ sequence files containing the raw reads using bcl2fastq (v2.20). Raw reads were demultiplexed with custom python (v3.8.12) scripts specifying a hamming distance ≤1 between query and reference barcodes. Demultiplexed reads were processed to convert any base calls with quality score <30 to ambiguous (‘N’). Processed reads with >10% ambiguous base calls within 40 bp of the edit site were discarded. To further validate reads, the first 40 bases (‘seed region’) of each read, which does not overlap the edit/nick sites for any of the amplicons in this study (minimum distance of edit/nick site from start of read is 75 bp), were compared to the expected (wild-type) amplicon sequence and reads with less than 90% homology were removed. Typically, >90% of reads were maintained through these initial filtering steps.

Filtered reads were aligned to the respective locus using the align.globalds() function within the pairwise2 module of Biopython (v1.78) which implements the Needleman-Wunsch algorithm as modified by Gotoh using a scoring criteria of 1, 0, −3, −1 for matches, mismatches, gap initiations and gap extensions, respectively. Defined region(s) of the alignment were then considered to classify outcomes with either of two methods (Supplementary Fig. 2). For method 1, which was used to analyze all samples in Fig. 1a,b and Supplementary Fig. 1d, a 40-nt region surrounding the pegRNA nick site (18 nt in 5′ direction, 22 nt in 3′ direction) and a 34-nt region surrounding the secondary sgRNA nick site (17 nt in each direction) designed for PE3 and PE5 strategies were considered. For method 2, which was used to analyze samples in all remaining figures of the text, only the 40-nt region surrounding the pegRNA nick site was considered since no secondary nick was employed in any of these groups. Defining alignment windows was necessary to reduce the effect of spontaneous (‘background’) single-nucleotide polymorphisms (SNPs) on determined error rates. These SNPs, observed initially in reads from unedited embryos, are hypothesized to be a result of (1) sequencing errors; (2) errors introduced during PCR1 of library preparation, which used a lower-fidelity, inhibitor-tolerant polymerase and high number of cycles to amplify target regions from embryonic lysates; and/or (3) somatic mutations. Aligned reads were binned into three sequence categories: (1) reads exactly matching the reference sequence within the considered region(s), (2) reads containing only the intended edit (‘precise edit’) within considered region(s) and (3) reads displaying an unintended sequence change (‘errors’) within the considered region(s). For all figures in the main text, the average background error rate (2–6% depending on the target site and analysis method) measured in unedited embryos was subtracted from each relevant sample when reporting error rates (‘adjusted errors’). To summarize editing rates across embryos within a given treatment group, mean and median editing rates are stated throughout the text and clearly distinguished. Results from the same treatment group were often used for multiple comparisons and thus included in multiple figures throughout.

### Determination of editing outcome

All samples, including controls, contained a background level of high-confidence SNPs spread variably across read sequences, presumably caused by errors introduced during PCR amplification, sequencing errors, and/or natural genetic variation, resulting in a low level of read misclassifications. To distinguish between samples containing precise edits and unedited samples, a strict cutoff of 1% of reads containing the precise edit was used. To determine samples containing significant levels of unintended byproducts as a result of prime editing, the percentage of reads classified as containing errors in unedited controls were used to construct a background model of noise for each target site which was approximated as normally distributed. Samples were compared against these distributions to determine embryos displaying significant errors using a one-sided *z*-test with Bonferroni adjusted *P* value cutoff of 0.001 (Supplementary Tables 7–14). All amplicon datasets from unedited controls, across different targets, sequencing runs and analysis methods (for example, with or without consideration of editing around a secondary, ‘nicked’ region) displayed a percentage of unedited wild-type reads ranging from 94–99% and a background percentage of reads classifying as errors (containing non-reference SNPs within the considered regions) between 1% and 6% (Supplementary Fig. 2). Microinjected samples deemed ‘unedited’, failing to show percentages of precise edit or error read classifications that met the criteria outlined above, therefore represent (1) levels of editing below the limit of detection imposed by the background and our statistical cutoffs or (2) the result of a failed microinjection.

### Whole-genome analysis

C57Bl/6J males and females were purchased from Charles River Laboratories. Three- to four-week-old females were superovulated as previously described^30,69^ and mated. Two-cell embryos were collected from individual females at E1.5 as previously described in M2 media^30,69^. Simultaneously, the spleen from each female was removed and retained for later genomic extraction. Embryos from a male/female pair were microinjected with the following mixes prepared in 15 μl total nuclease-free injection buffer: PE4 mouse family, nCas9-RT mRNA (PE2 editor, 100 ng μl^−1^), pegRNA (75 pmol) and mMlh1dn mRNA (200 ng μl^−1^) or nCas9-RT mRNA (PE2 editor, 100 ng μl^−1^) alone; PE2* mouse family, nCas9-RT mRNA (PEMax editor, 100 ng μl^−1^) and pegRNA (75 pmol) or pegRNA (75 pmol) alone; mMLH1dn mouse family, pegRNA (75 pmol) and mMlh1dn mRNA (200 ng μl^−1^) or pegRNA (75 pmol) alone. After microinjections, embryos from a single C57Bl/6J female/male pair were immediately transferred into pseudopregnant CD1 females and gestated for 11 more days (E12.5). Recipient females were sacrificed and E12.5 embryos removed from individual decidua. Half of each embryo was processed for gDNA extraction. The corresponding sire for the embryos was sacrificed, the spleen removed and retained for gDNA extraction. Using the PureLink Genomic DNA Kit (Invitrogen), 10 mg spleen or embryo tissue was processed according to manufacturer’s specifications. Briefly, tissue was lysed in 180 μl Genomic Digestion Buffer with 20 μl freshly added Proteinase K. The tissue was incubated for at least 4 h at 55 °C until completely lysed, after which 20 μl RNase A was added. RNA was degraded for 2 min at room temperature, 200 μl Binding Buffer and 200 μl of 100% ethanol added and the resulting gDNA purified by column.

Dual-indexed sequencing libraries were prepared from 100–200 ng purified gDNA per sample using the Tagmentation-based Illumina DNA prep kit (20018704) according to manufacturer specifications including 5–8 cycles of PCR and double-sided size selection to enrich for library fragments between 300 and 600 bp. Libraries from the PE4 mouse family (*n* = 7) were pooled at equal concentration and sequenced on a Novaseq 6000 using an S4 flow cell and V1.5 reagent kit with read structure 150 × 8 × 8 × 150 (R1, I7, I5 and R2, respectively). Libraries from the mMLH1dn family (*n* = 6) and PE2* family (*n* = 4) were pooled and sequenced in the same manner in a separate S4 run. Reads were merged across lanes with Samtools (v1.15.1), demultiplexed using fastq-multx (v1.4.2) allowing up to two mismatches (-m 2) and requiring a minimum distance of two (-d 2) between the best and second best barcode matches, and trimmed/filtered using Trimmomatic (v0.39) with arguments ILLUMINACLIP:adapters.fa:2:30:10 LEADING:20 TRAILING:20 SLIDINGWINDOW:4:20 MINLEN:50. Filtered, paired reads were aligned to the NCBI’s mouse reference genome assembly GRCm39 (GCF_000001635.27) using the bwa mem algorithm (v0.7.17) with default parameters. Alignment rates exceeded 99% for all samples. For the mMLH1dn mouse family, one control offspring was removed from the dataset after reporting a significantly lower percentage of properly paired reads (93% versus 99% for all other samples). Aligned reads were deduplicated using Picard (v2.27.1) MarkDuplicates() function with argument Remove_Duplicates = True, which retained 70–80% of reads per sample. Final genomic coverage for samples from PE4 family ranged between 100× and 140× after deduplication. Coverage for samples from the mMLH1dn family and PE2* family ranged between 70× and 90× after deduplication and were subsequently down sampled to 70× for variant analysis using samtools view. Variant calling was performed with GATK (v4.2.6.1) HaplotypeCaller with included argument–dont-use-soft-clipped-bases and final joint analysis for samples within a given family was performed using GenotypeGVCFs. The resulting .vcf files were analyzed with custom Python (v3.8.12) scripts.

### Off-target analysis

To evaluate potential off-target mutations in whole-genome-sequenced mice/embryos as a result of prime editing, candidate sites were predicted based on the *Chd2* +5 G>A pegRNA spacer (5′-GCGGTAGCTCCCAGAACGGT-3′) using Cas-OFFinder^70^, specifying the 5′-NGG-3′ PAM requirement and allowing up to four mismatches and a maximum DNA/RNA bulge = 2 nt. No variants were detected within 100 bp of the identified off-target regions (*n* = 38 sites) for edited offspring within the PE4 mouse family (Supplementary Table 21). To initially evaluate genomic stability, 12 microsatellite regions (U12235, AA003063, L24372, D1Mit79, D9Mit67, D1Mit355, D4Mit27, D15Mit59, D14Mit15, D18Mit15, D7Mit91 and D10Mit2) and two genes (*Tgfbr2* and *Bax*) were selected based off previous studies of mouse *Mlh1* deficiency (Supplementary Table 19). The number of variants detected in these regions for each sample within the PE4 mouse family was determined from .vcf files generated by joint variant analysis of aligned WGS reads and compared between edited and unedited samples (Supplementary Fig. 14). For global analysis of genome stability, unique variants were defined as being detected in only one sample within a given family, requiring a minimum depth of 30 aligned reads at the reported genomic position for each sequenced sample within the family and a minimum variant allele (read) frequency of 0.2 within the sample in which the variant was detected. Variants within sex chromosomes (NC_000086.8 and NC_000087.8) were not considered.

### Predicting prime editing efficiency

Prime editing efficiencies were predicted using fine-tuned deep-learning-based models trained on results of applying different prime editing systems to perform a range of edits in HEK293T cells^44^. Inputting 121-nt sequences specifying the unedited and edited target site, the models predict prime editing efficiencies, reported as DeepPrime scores, for all pegRNA designs that enable the edit. Default parameters constraining the pegRNA design space for any given edit were used including a maximum reverse transcription template of 40 nt and a primer binding site ranging from 1 to 17 nt. For Fig. 1f and Supplementary Fig. 11b,c, we chose to focus on the ‘PE4max with optimized scaffold in HEK293Ts’ DeepPrime-FT model when comparing to PE4 results observed in mouse embryos.

### Reporting summary

Further information on research design is available in the Nature Portfolio Reporting Summary linked to this article.

## Online content

Any methods, additional references, Nature Portfolio reporting summaries, source data, extended data, supplementary information, acknowledgements, peer review information; details of author contributions and competing interests; and statements of data and code availability are available at 10.1038/s41587-023-02106-x.

## Supplementary information

Supplementary Information Supplementary Figs. 1–22.

Reporting Summary

Supplementary Data Supplementary Tables 1–21.

## Data Availability

Demultiplexed sequence datasets for all samples included in this work are available on NCBI’s Sequence Read Archive through BioProject accession PRJNA1040158 (ref. ^71^). Metadata on all collected samples are included in Supplementary Tables 7–14. Variant call formatted (VCF) files from WGS analysis are available at https://github.com/badamsonlab/PEmbryo/. Any additional information is available from the corresponding authors upon request.

## Code availability

All relevant code from this study including scripts to determine editing efficiencies from target sequencing data and variant classification from WGS analysis, as well as code to reproduce published figures, are available at https://github.com/badamsonlab/PEmbryo (ref. ^72^).
