# Supplementary material for: Efficient prime editing in two-cell mouse embryos using PEmbryo
Source: Nat Biotechnol. 2024 Feb 6;42(12):1822–30. doi: 10.1038/s41587-023-02106-x (PMC11631759; doi:10.1038/s41587-023-02106-x)
Supplement: Supplementary file 1 — Supplementary Figs. 1–22. [file 41587_2023_2106_MOESM1_ESM.pdf]

nature biotechnology

Brief Communication https://doi.org/10.1038/s41587-023-02106-x

Efficient prime editing in two-cell mouse embryos using PEmbryo

In the format provided by the authors and unedited

Supplementary Information

Efficient prime editing in two-cell mouse embryos using PEmbryo

Supplementary Figure 1. Overview of prime editing approach.

Supplementary Figure 2. Target sequencing of unedited mouse embryos.

Supplementary Figure 3. Fraction of base calls disagreeing from the reference sequence for classified reads across embryos.

Supplementary Figure 4. Unintended byproducts generated at the *Rnf2* target site when editing with PE3 and PE5 at the zygote stage.

Supplementary Figure 5. Unintended byproducts generated at the *Chd2* target site when editing with PE3 and PE5 at the zygote stage.

Supplementary Figure 6. Comparison of editing outcome frequencies across prime editing methods in embryos edited at the two-cell stage.

Supplementary Figure 7. Unintended byproducts generated at the *Rnf2* target site when editing with PE3 and PE5 at the two-cell stage.

Supplementary Figure 8. Unintended byproducts generated at the *Chd2* target site when editing with PE3 and PE5 at the two-cell stage.

Supplementary Figure 9. Optimization of prime editing conditions in the early mouse embryo.

Supplementary Figure 10. Testing optimized prime editing components in embryos.

Supplementary Figure 11. Effect of mMLH1dn on prime editing efficiency for edits with contiguous substitutions in embryos.

Supplementary Figure 12. Editing efficiencies for insertions of different lengths in *Hoxd13* with PEmbryo.

Supplementary Figure 13. Comparison of PEmbryo editing efficiencies to predictions from DeepPrime[44].

Supplementary Figure 14. *Chd2* editing outcome frequencies in PEmbryo edited mice.

Supplementary Figure 15. PEmbryo editing in common mouse strains.

Supplementary Figure 16. Assessment of genomic stability at select microsatellite regions.

Supplementary Figure 17. Genomic distribution of unique -1 bp deletions in samples from PE4 mouse family.

Supplementary Figure 18. Genomic distribution of unique -2 bp deletions in samples from PE4 mouse family.

Supplementary Figure 19. Number of unique SNVs by type for treated and control samples in mouse families.

Supplementary Figure 20. Number of unique indels by type for treated and control samples in mouse families.

Supplementary Figure 21. Sequence motifs near unique deletions detected in whole genome sequenced mouse families.

Supplementary Figure 22. *Hoxd13* editing outcome frequencies in PEmbryo edited mice.

a

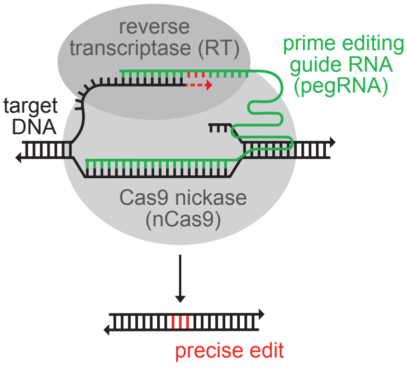

b *Rnf2* +1 C>G substitution edit

pegRNA nick ▼

AGGATGTATTATATTACCTGAGGTGTTCGTTGTAACTC[...]$_{25}$CTTAATGGTTGAGTTCCATTTGTCTGCACAGCCTGAGA

G edit

▲ sgRNA nick on opposite strand

c *Chd2* +5 G>A substitution edit

pegRNA nick ▼

GCGGTAGCTCCCAGAACGGTGGGCATCCATATGCCTCC[...]$_{10}$GTGGTGGTCCTTATACCAATGCTGCTCATACTGATGGT

A edit

▲ sgRNA nick on opposite strand

Supplementary Figure 1. Overview of prime editing approach. a, Schematic of prime editing including the nCas9-RT editor protein and prime editing guide RNA (pegRNA) **b,** *Rnf2* +1 C>G substitution edit with target sequence (light gray), PAM (dark gray), and nick sites (arrows) used in this study indicated. **c,** *Chd2* +5 G>A substitution edit with target sequence (light gray), PAM (dark gray), and nick sites (arrows) used in this study indicated.

a

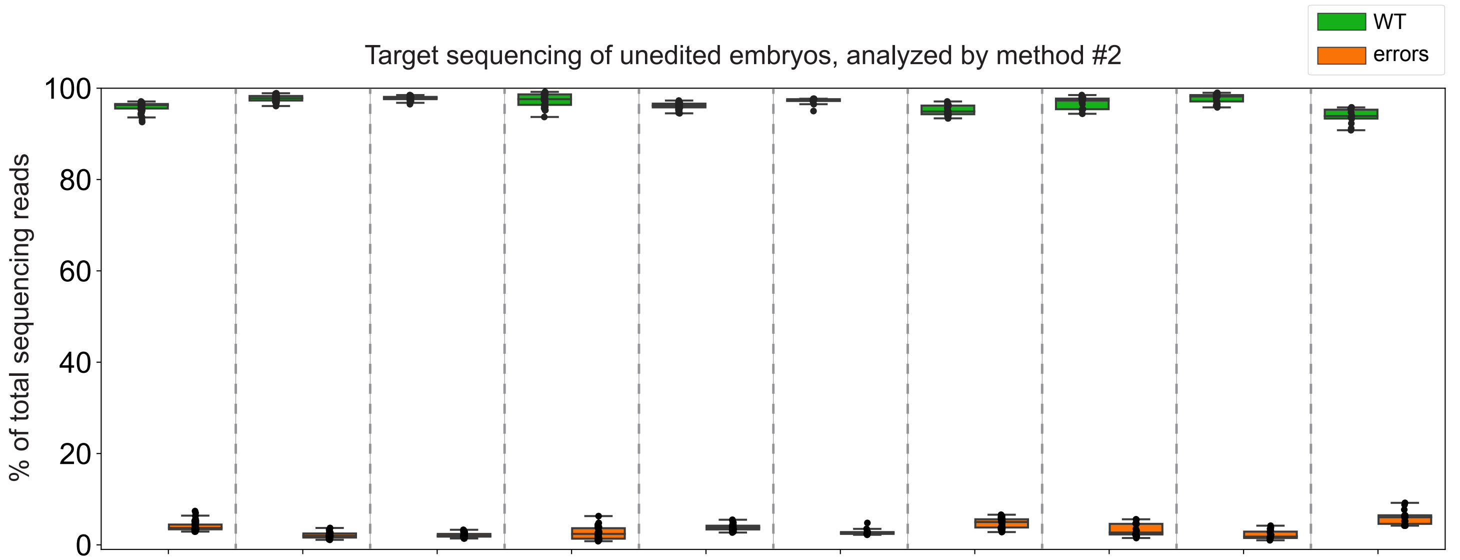

**Target**	*Dnmt1*	*Rnf2*	*Chd2*	*Hoxd13_1*	*Hoxd13_2*	*Col12a1*	*Ctnnb1*	*Crygc*	*Tspan2*	*Tubb5*
**Method**	control	control	control	control	control	control	control	control	control	control
**# Embryos**	71	47	41	42	40	13	29	16	21	14
**Avg. Error**	4.0%	2.1%	2.1%	2.5%	3.8%	2.8%	4.8%	3.2%	2.3%	6.0%

b **Example of reads classified as errors in unedited embryos, analysis method #1 (Figure 1a-b)**

Rnf2 locus

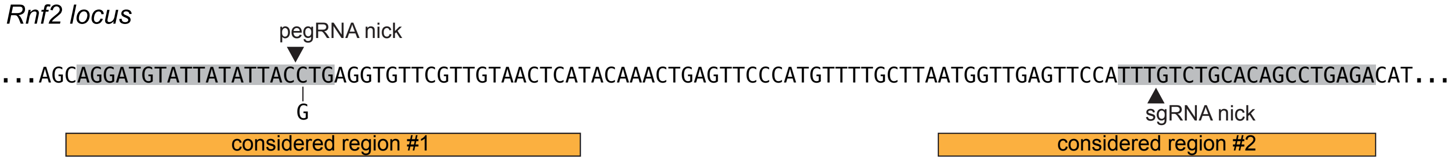

AGCAGGATGTATTATATTACCTGAGGTGTTCGTTGTAACTCATACAAACTGAGTTCCCATGTTTTGCTTAATGGTTGAGTTCCATTTGTCTACACAGCCTGAGACAT
AGCAGGATGTATTATATTACCTGAGGTGTTTGTTGTAACTCATACAAACTGAGTTCCCATGTTTTGCTTAATGGTTGAGTTCCATTTGTCTGCACAGCCTGAGACAT
AGCAGGATGTATTATATTACCTGAGGTGTTCGTTGTAACTCATACAAACTGAGTTCCCATGTTTTGCTTAATGGTTGAGTTCCATTTGTCTGCACAGCCTAAGACAT
AGCAGGATGTATTATGTTACCTGAGGTGTTCGTTGTAACTCATACAAACTGAGTTCCCATGTTTTGCTTAATGGTTGAGTTCCATTTGTCTGCACAGCCTGAGACAT
AGCAGGATGTATTATATTACCTGAGGTGTTCGTTGTAACTCATACAAACTGAGTTCCCATGTTTTGCTTAATGGTTGAGTTCCATTTGTCGGCACAGCCTGAGACAT
AGCAGGATGTATTATATTACCTGAGGTGTTCGTTGTAACTCATACAAACTGAGTTCCCATGTTTTGCTTAATGATTGAGTTCCATTTGTCTGCACAGCCTGAGACAT

Background error frequency (*Rnf2,* method #1) = 4.2 ± 0.9

c **Example of reads classified as errors in unedited embryos, analysis method #2 (All figures except Figure 1a-b)**

Rnf2 locus

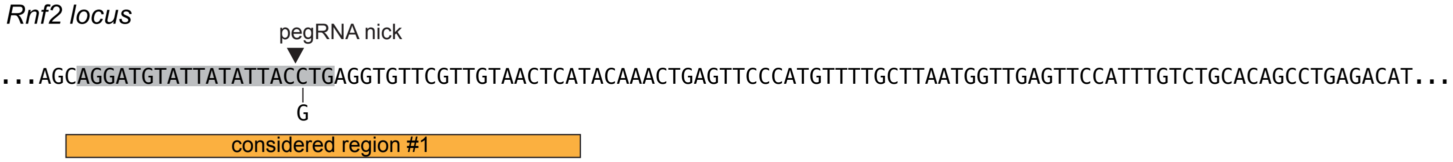

AGCAGGATGTATTATGTTACCTGAGGTGTTCGTTGTAACTCATACAAACTGAGTTCCCATGTTTTGCTTAATGGTTGAGTTCCATTTGTCTGCACAGCCTGAGACAT
AGCAGGATGTATTATATTACCTGAGGTGTTCGTTGCAACTCATACAAACTGAGTTCCCATGTTTTGCTTAATGGTTGAGTTCCATTTGTCTGCACAGCCTGAGACAT
AGCAGGATGTATCATATTACCTGAGGTGTTCGTTGTAACTCATACAAACTGAGTTCCCATGTTTTGCTTAATGGTTGAGTTCCATTTGTCTGCACAGCCTGAGACAT
AGCGGGATGTATTATATTACCTGAGGTGTTCGTTGTAACTCATACAAACTGAGTTCCCATGTTTTGCTTAATGGTTGAGTTCCATTTGTCTGCACAGCCTGAGACAT
AGCAGGATGTATTATATTACCTGAGGTGTTCGTTGTAACACATACAAACTGAGTTCCCATGTTTTGCTTAATGGTTGAGTTCCATTTGTCTGCACAGCCTGAGACAT
AGCAGGATGTATTATATTACCTGAGGTGTTCGTTGCAACTCATACAAACTGAGTTCCCATGTTTTGCTTAATGGTTGAGTTCCATTTGTCTGCACAGCCTGAGACAT

Background error frequency (*Rnf2,* method #2) = 2.1 ± 0.5

Supplementary Figure 2. Target sequencing of unedited mouse embryos. a, Percentage of total reads classified as wild-type (“WT”, green) or containing errors (“errors”, orange) from control embryos using analysis method #2 which does not consider a secondary nick site (Methods, Supplementary Figures 1b-c). Each datapoint represents an individual embryo. Target site, group size, and the average percentage of reads containing an error near the edit site across embryos are indicated. Data are compiled from separate experiments (Supplementary Table 7, Methods). Box plots indicate the median and interquartile range (IQR) of each group with whiskers extending 2*IQR past the upper and lower quartiles. **b,** Top) *Rnf2* locus with the regions considered during editing outcome determination when analyzing reads by method #1, as in Figures 1a-b and Supplementary Figure 6, which considers both a primary edit site and a secondary nick site to account for PE3 and PE5 methods. Bottom) Example of reads classified as containing errors from unedited embryos analyzed by method #1 **c,** Top) *Rnf2* locus with the regions considered for editing outcome determination when analyzing reads by method #2, as in all figures except Figure 1a-b and Supplementary Figure 6. Bottom) Example of reads classified as containing errors from unedited embryos analyzed by method #2.

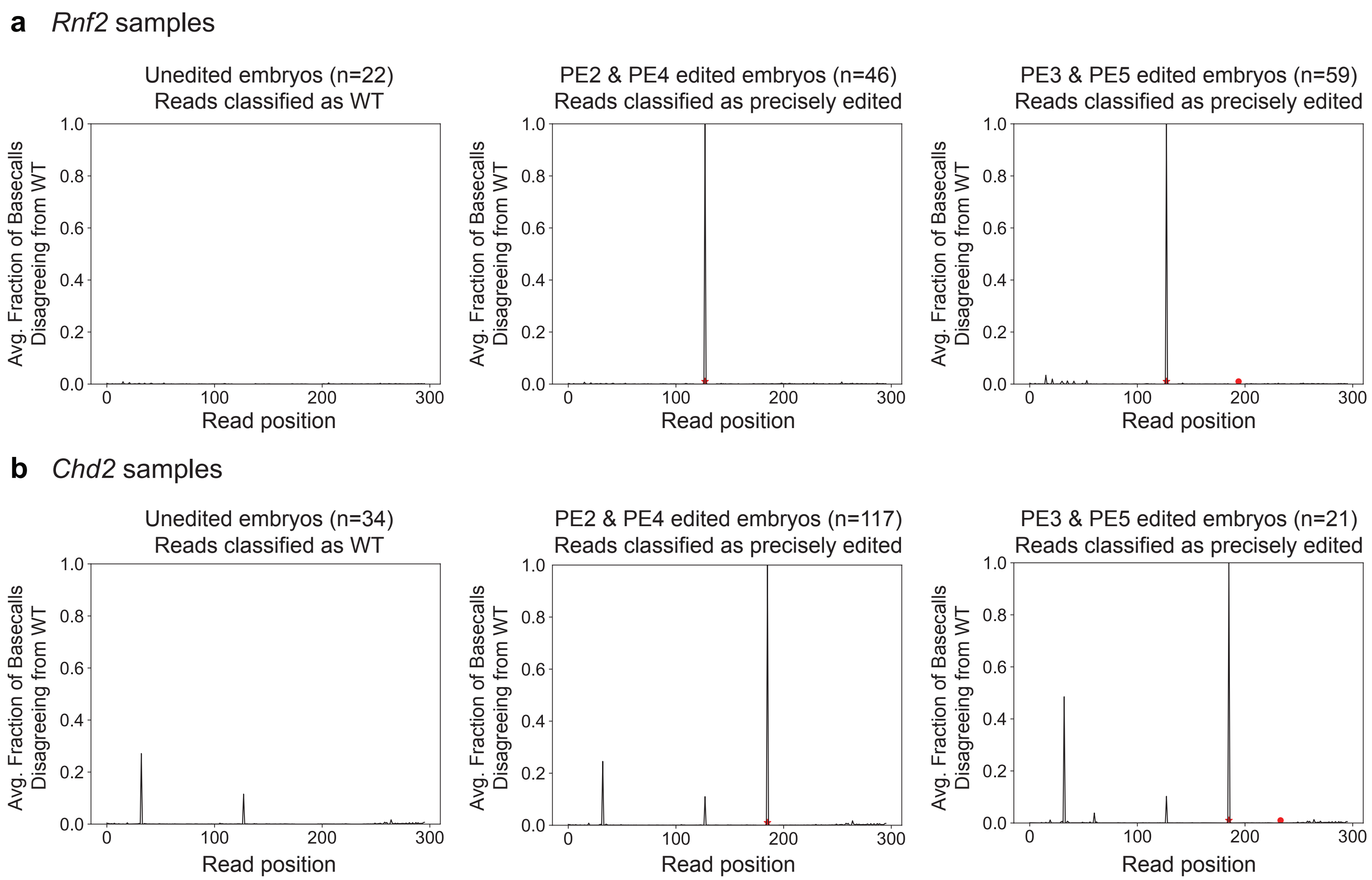
a Rnf2 samples
Unedited embryos (n=22)
Reads classified as WT
PE2 & PE4 edited embryos (n=46)
Reads classified as precisely edited
PE3 & PE5 edited embryos (n=59)
Reads classified as precisely edited
b Chd2 samples
Unedited embryos (n=34)
Reads classified as WT
PE2 & PE4 edited embryos (n=117)
Reads classified as precisely edited
PE3 & PE5 edited embryos (n=21)
Reads classified as precisely edited
Avg. Fraction of Basecalls Disagreeing from WT
Read position
0.0
0.2
0.4
0.6
0.8
1.0
0
100
200
300

Supplementary Figure 3. Fraction of base calls disagreeing from the reference sequence for classified reads across embryos. a, The average fraction of base calls from *Rnf2* samples differing from the reference amplicon sequence for all reads classified as “wild-type” (for unedited embryos) or “precisely edited” (for edited embryos with > 100 reads classified as precisely edited) along the length of the amplicon. Star symbol denotes the position of the intended edit. Red circle marks the secondary nick site for embryos edited with PE3 or PE5. **b**, Same as (a) but for *Chd2* samples. Editing results are compiled from multiple experiments (Supplementary Tables 7-8, Methods) and represent the same datasets as illustrated in Figure 1a-b.

a

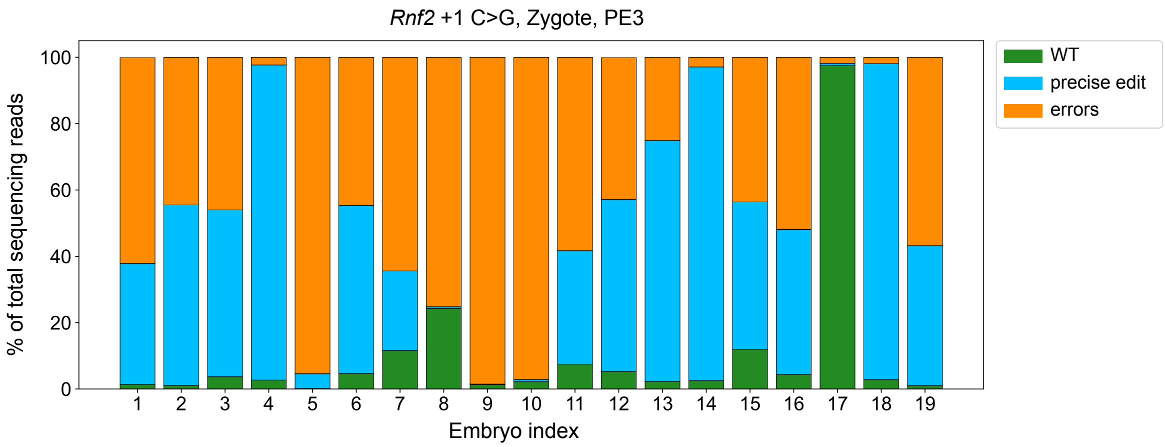

Allele frequency amongst reads with errors

```
Ref ACAGCAGGATGTATTATATTACCTGAGGTGTTCGTTGTAACTCATACAAACTGAGTTCCCATGTTTTGCTTAATGGTTGAGTTCCATTTGTCTGCACAGCCTGAGACATTTCTAGGAATAAAA

 1  ACAGCAGGATGTATTATATTACGTGAGGTGTTCGTTGTAACTCATACAAACTGAGTTCCCATGTTTTGC--------------------------ACAGCCTGAGACATTTCTAGGAATAAAA   77%
 2  ACAGCAGGATGTATTATATTACGTGAGGTGTTCGTTGCTTA-------------------ATGTTTTGCTTAATGGTTGAGTTCCATTTGTCTGCACAGCCTGAGACATTTCTAGGAATAAAA   91%
 3  ACAGCAGGATGTATTATATTACCTGAGGTGTTCGTTGTAACTCATACAAACTGAGTTCCCATGTTTTGCTTAATGGTTGAG------------------CCTGAGACATTTCTAGGAATAAAA   88%
 4  ACAGCAGGATGTATTATATTACGTGAGGTGTTCGTTGTAACTCATACAAACTGAGTTCCCATGTTTTGCTTAATGGTTGAGTTCCATTTGTCTGCACGGCCTGAGACATTTCTAGGAATAAAA    8%
 5  ACAGCAGGATGTATTATATTATGTGAGGTGTTCGTTG---------------------------------------------------------CACAGCCTGAGACATTTCTAGGAATAAAA   76%
 6  ACAGCAGGATGTATTATATTACGTGAGGTGTTCGTTGTAACTCATACAAACTGAGTTCCCATGT---------------------------CTGCACAGCCTGAGACATTTCTAGGAATAAAA   55%
 7  ACAGCAGGATGTATTATATTACGTGAGGTGTTCGTTGTAACTCATACAAACTGAGTTCCCATGTTTTGCTTAATGGTTGAGTTCCATTTTGTCTGCACAGCCTGAGACATTTCTAGGAATAAAA   48%
 8  ACAGCAGGATGTATTATATTACCTGAGGTGTTCGTTGTAACTCATACAAACTGAGTTCCCATGTTTTGC--------------------------ACAGCCTGAGACATTTCTAGGAATAAAA   77%
 9  ACAGCAGGATGTATTATATTACCTGAGGTGTTCGTTGTAACTCATACAAACTGAGTTCCCATGTTTTGCTTAATGGT-----------------CTCAGCCTGAGACATTTCTAGGAATAAAA   57%
10  ACAGCAGGATGTATTATATTACCTGAGGTGTTCGTTGTAACTCATACAAACTGAGTTCCCATGTTTTG--------------------------CACAGCCTGAGACATTTCTAGGAATAAAA   81%
11  ACAGCAGGATGTATTATATTACGTGAGGTG----------------------------------------------------------------CACAGCCTGAGACATTTCTAGGAATAAAA   59%
12  ACAGCAGGATGTATTATATTACGTGAGGTGTTCGTTG---------------------------------------------------------CACAGCCTGAGACATTTCTAGGAATAAAA   66%
13  ACAGCAGGATGTATTATATTACGTGAGGTGTTCGTTGTAACTCATACAAACTGAGTTCCCATGTTTTGC--------------------------ACAGCCTGAGACATTTCTAGGAATAAAA   78%
14  CAGCAGGATGTATTATATTACGTGAGGTGTTCGTTGTAACTCATACAAACTGAGTTCCCATGTTTTGCTTAATGGTTGAGTTCCATTTGTCTGCACAGCCTGAGACATTTCTAGGAATAAAAA   11%
15  ACAGCAGGATGTATTATATTACGTGAGGTGTTCGTTG---------------------------------------------------------CACAGCCTGAGACATTTCTAGGAATAAAA   84%
16  ACAGCAGGATGTATTATATTACGTGAGGTGTTCGTTGTAACTCATACAAACTGAG--------------------------TTCCATTTGTCTGCACAGCCTGAGACATTTCTAGGAATAAAA   55%
17  ACAGCAGGATGTATTATATTACCTGAGGTGTTCGTTGTAACTCATACAAACTGAGTTCCCATGTTTTGCTTAATGGTTGAGTTCCATTTGTCTGCATAGCCTGAGACATTTCTAGGAATAAAA    4%
18  ACAGCAGGATGTATTATATTACGTGAGGTGTTCGTTGTAACTCATACAAACTGAGTTCCCATGTTTTGCTTAATGGTTGAGTTCCATTTGTCTACACAGCCTGAGACATTTCTAGGAATAAAA    5%
19  ACAGCAGGA------------------------------------------------------------------------------------------------------------ATAAAA   91%
```

b

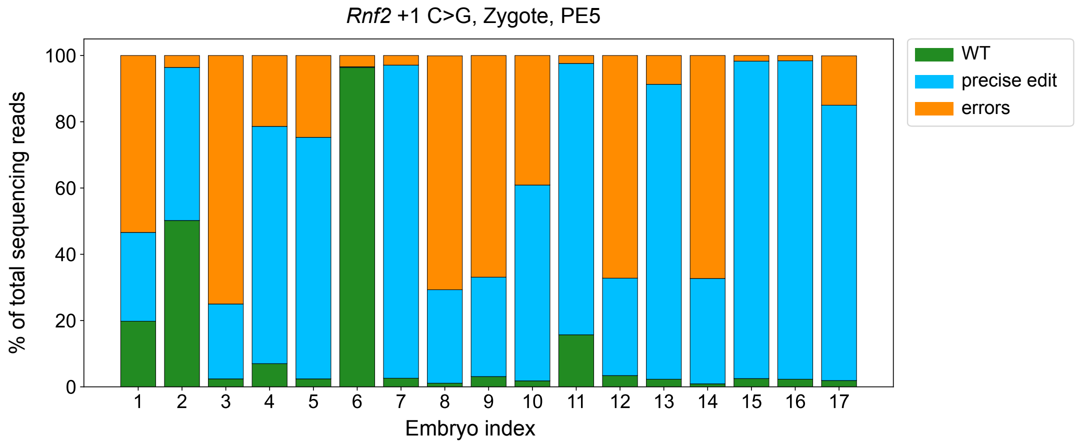

Allele frequency amongst reads with errors

```
Ref ACAGCAGGATGTATTATATTACCTGAGGTGTTCGTTGTAACTCATACAAACTGAGTTCCCATGTTTTGCTTAATGGTTGAGTTCCATTTGTCTGCACAGCCTGAGACATTTCTAGGAATAAAA

 1  ACAGCAGGATGTATTATATTACGTGAGGTGTTCGTTGTAACTCATACAAACTGAGTTCCCATGTTTTGCTTAATGGTTGAG------------------------ACATTTCTAGGAATAAAA   80%
 2  ACAGCAGGATGTATTATATTACGTGAGGTGTTCGTTG---------------------------------------------------------CACAGCCTGAGACATTTCTAGGAATAAAA    6%
 3  ACAGCAGGATGTATTATATTACGTGAGGTGTTCGTTG---------------------------------------------------------CACAGCCTGAGACATTTCTAGGAATAAAA   90%
 4  ACAGCAGGATGTATTATATTACGTGAGGTGTTCGTTGTAACTCATACAAACTGA----------------------------------------------------CATTTCTAGGAATAAAA   44%
 5  ACAGCAGGATGTATTATATTACGTGAGGTGTTCGTTGTAA---------------------------------TGGTTGAGTTC--------TGCACAGCCTGAGACATTTCTAGGAATAAAA   87%
 6  ACAGCAGGATGTATTATATTACCTGAGGTGTTCGTTGTAACTCATACAAACTGAGTTCCCATGTTTTGCTTAATGGCTGAGTTCCATTTGTCTGCACAGCCTGAGACATTTCTAGGAATAAAA    7%
 7  ACAGCAGGATGTATTATATTACGTGAGGTGTTCGTTGTAACTCATACAAACTGAGTTCCCATGTTTTGCTTAATGGTTGAGTTCCATTTGTCTGTACAGCCTGAGACATTTCTAGGAATAAAA    6%
 8  ACAGCAGGATGTATTATATTACGTGAGGTGTTCGTTG[insertion]28[deletion]14TGAGTTCCCATGTTTTGCTTAATGGTTGAGTTCCATTTGTCTGCACAGCCTGAGACATTTCTAGGAATAAAA   91%
 9  ACAGCAGGATGTATTATATTACGTGAGGTGTTCGTTGTA------------------------------------------------------------------------------------   52%
10  ACAGCAGGATGTATTATATTACGTGAGGTGTTCGTTG---------------------------------------------------------CACAGCCTGAGACATTTCTAGGAATAAAA   78%
11  ACAGTAGGATGTATTATATTACGTGAGGTGTTCGTTGTAACTCATACAAACTGAGTTCCCATGTTTTGCTTAATGGTTGAGTTCCATTTGTCTGCACAGCCTGAGACATTTCTAGGAATAAAA    5%
12  ACAGCAGGATGTATTATATTACCTGAGGTGTTCGTTGT-----------------------------------------------------CTGCACAGCCTGAGACATTTCTAGGAATAAAA   48%
13  ACAGCAGGATGTATTATATTACGTGAGGTGTTCGTTGTA------------------------------------------------------GCACAGCCTGAGACATTTCTAGGAATAAAA   68%
14  ACAGCAGGATGTATTATATTACGTGAGGTGTTCGTTG---------------------------------------------------------CACAGCCTGAGACATTTCTAGGAATAAAA   94%
15  ---------------------------------------------------------------------------------------------------------------------------    7%
16  ACAGCAGGATGTATTATATTACGTGAGGTGTTCGTCGTAACTCATACAAACTGAGTTCCCATGTTTTGCTTAATGGTTGAGTTCCATTTGTCTGCACAGCCTGAGACATTTCTAGGAATAAAA    7%
17  ACAGCAGGATGTATTATATTACGTGAGGTGTTCGTTGTAACTCATACAAACTGAGTTCCCATGTTTTGC--------------------------ACAGCCTGAGACATTTCTAGGAATAAAA   86%
```

Supplementary Figure 4. Unintended byproducts generated at the *Rnf2* target site when editing with PE3 and PE5 at the zygote stage. a, Top) Percentages of classified reads from individual embryos microinjected with PE3 editing components at the zygote stage. For each embryo, the programmed edit was a +1 C>G substitution in *Rnf2*. Bottom) Aligned sequence and percentage of the most common byproduct observed in each embryo. Percentages calculated from all reads with an unintended modification. Bolded green letters indicate the precise edit. Bolded red letters indicate errors at the target site. Dashed lines represent deletions. **b,** Same as (a) but for editing performed with PE5 components. Editing results are compiled from multiple experiments (Supplementary Table 8, Methods) and represent the same datasets as illustrated in Figure 1a.

a

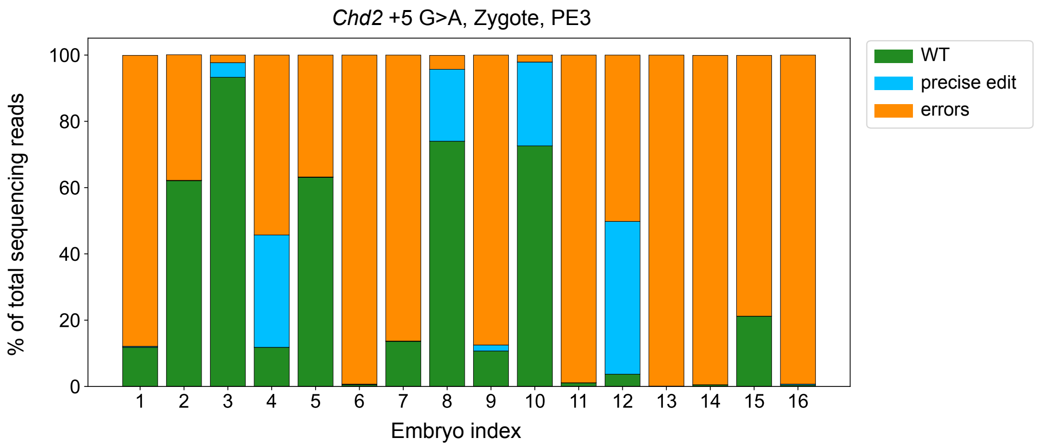


```
                                                                                                                                   Allele frequency
                                                                                                                                   amongst reads
Ref TCTGGACATGTTGTTAGGGCGGTAGCTCCCAGAACGGTGGGCATCCATATGCCTCCGGTCACCATAGTGGTGGTCCTTATACCAATGCTGCTCATACTGATGGTGCCGGTCGCCCCCCCAGGG  with errors

 1  TCTGGACATGTTGTTAGGGCGGTAGCTCCCAGAACGGTGAGCATC--------------------------------------------GCTCATACTGATGGTGCCGGTCGCCCCCCCAGGG  34%
 2  ------------------------------------------------------------ACCATAGTGGTGGTCCTTATACCAATGGTGCCGTATGAGACTATTATG------------GGG  61%
 3  TCTGGACATGTTGTTAGGGCAGTAGCTCCCAGAACGGTGGGCATCCATATGCCTCCGGTCACCATAGTGGTGGTCCTTATACCAATGCTGCTCATACTGATGGTGCCGGTCGCCCCCCCAGGG  27%
 4  TCTGGACATGTTGTTAGGGCGGTAGCTCCCAGAACTC-----------------------------------------------ATACTGTTCATACTGATGGTGCCGGTCGCCCCCCCAGGG  44%
 5  TCTGGACATGTTGTTAGGGCGGTAGCTCCCAGAACGGTGGGCATCCATATGCCTCC--------------------------CAACGCTGCTCATACTGATGGTGCCGGTCGCCCCCCCAGGG  82%
 6  ---------------------------------------------------------------------------------------------------------------------------  56%
 7  TCTGGACATGTTGTTAGGGCGGTAGCTCCCAGAAC----------------------------------------------------------------------------------------  27%
 8  TCTGGACATGTTGTTAGGGCGGTAGCTCCCAGAACGGTGAGCATC--------------------------------------------GCTCATACTGATGGTGCCGGTCGCCCCCCCAGGG  45%
 9  TCTGGACATGTTGTTAGGGCGGTAGCTCCCAGAACGGTG---------------------------------------------------CTCATACTGATGGTGCCGGTCGCCCCCCCAGGG  36%
10  TCTGGACATGTTGTTAGGGCGGTAGCTCCCAGAACGGTGCGCATCCATATGCCTCCGGTCACCNTAGTGGTGGTCCTTATACCAATGCTGCTCATACTGATGGTGCCGGTCGCCCCCCCAGGG   5%
11  TCTGGACATGTTGTTAGGGCGGTAGCTCCCAGAACGGTGGGCATCCATATGCCTCCGGTCACCATAGTGGTGGTCCTTATAC------TGCTCATACTGATGGTGCCGGTCGCCCCCCCAGGG  50%
12  TCTGGACATGTTGTTAGGGCGGTAGCTCCCAG-----------------------------------------------------------------------------------------GG  53%
13  TCTGGACATGTTGTTAGGGCGGTAGCTC-----------------------------------------------------------------ATACTGATGGTGCCGGTCGCCCCCCCAGGG  46%
14  ---------------------------------------------------------------------------------------------ATACTGATGGTGCCGGTCGCCCCCCCAGGG  41%
15  TCTGGACATGTTGTTAGGGCGGTAGCTCCCAGAACGGTGA-------------------------------------------------GCTCATACTGATGGTGCCGGTCGCCCCCCCAGGG  34%
16  TCTGGACATGTTGTTAGGGCGGTAGCTCCCAGAACGGTGAGCATCCATATGCCTCCGGTCACCA--------------------------------------------------------GGG  30%
```

b

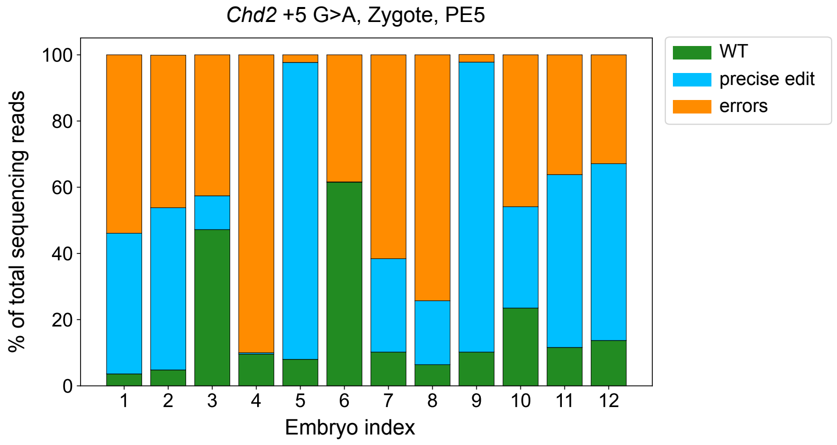


```
                                                                                                                                   Allele frequency
                                                                                                                                   amongst reads
Ref TCTGGACATGTTGTTAGGGCGGTAGCTCCCAGAACGGTGGGCATCCATATGCCTCCGGTCACCATAGTGGTGGTCCTTATACCAATGCTGCTCATACTGATGGTGCCGGTCGCCCCCCCAGGG  with errors

 1  TCTGGACATGTTGTTAGGGCGGTAGCTC-----------------------------------------------------------------ATACTGATGGTGCCGGTCGCCCCCCCAGGG  78%
 2  TCTGGACATGTTGTTAGGGCGGTAGCTCC---------------------------GGTCACCATAGTGGTGGTCCTTATACCAATGCTGCTCATACTGATGGTGCCGGTCGCCCCCCCAGGG  69%
 3  TCTGGACATGTTGTTAGGGCGGTAGCTCCCAG-----------------------------------------------------------------------------------------GG  50%
 4  TCTGGACATGTTGTTAGGGCGGTAGCTCCCAGAACGGTGAGCATC------------------------------------------------------------------------------  43%
 5  TCTGGACATGTTGTTAGGGCGGTAGCTCCCAGAACGGTGCGCATCCATATGCCTCCGGTCACCATAGTGGTGGTCCTTATACCAATGCTGCTCATACTGATGGTGCCGGTCGCCCCCCCAGGG  11%
 6  TCTGGACATGTTGTTAGGGCGGTAGCTCCCAG-----------------------------------------------------------------------------------------GG  90%
 7  -------------------------------------------------------------------------------------------------TGATGGTGCCGGTCGCCCCCCCAGGG  50%
 8  TCTGGACATGTTGTTAGGGCGGTAGC-------------------------------------------------------------------------------------------------  42%
 9  TCTGGACATGTTGTTAGGGCGGTAGCTCCCAGAACGGTGCGCATCCATATGCCTCCGGTCACCATAGTGGTGGTCCTTATACCAATGCTGCTCATACTGATGGTGCCGGTCGCCCCCCCAGGG  27%
10  TCTGGACATGTTGTTAGGCCGGT---------------------------------------------------------------------------------------CGCCCCCCCAGGG  49%
11  TCTGGACATGTTGTTAGGGCGGTAGCTCCCAGAACGGTG---------------------------------------------------CTCATACTGATGGTGCCGGTCGCCCCCCCAGGG  89%
12  TCTGGACATGTTGTTAGGGCGGTAGCTCCCAGAACGGTGAGCATCCATATGCCTCCGGTCACCATAGTGGTGGTCCTTATACCAATGCAATGCTGCTCATACTGATGGTGCCGGTCGCCCCCC  47%
```

Supplementary Figure 5. Unintended byproducts generated at the *Chd2* target site when editing with PE3 and PE5 at the zygote stage. a, Top) Percentages of outcome-classified reads from individual embryos microinjected with PE3 editing components at the zygote stage. For each embryo, the programmed edit was a +5 G>A substitution in *Chd2*. Bottom) Aligned sequence and percentage of the most common byproduct observed in each embryo. Percentages calculated from all reads with an unintended modification. Bolded green letters indicate the precise edit. Bolded red letters indicate errors at the target site. Dashed lines represent deletions. **b,** Same as (a) but for editing performed with PE5 components. Editing results are compiled from multiple experiments (Supplementary Table 8, Methods) and represent the same datasets as illustrated in Figure 1a.

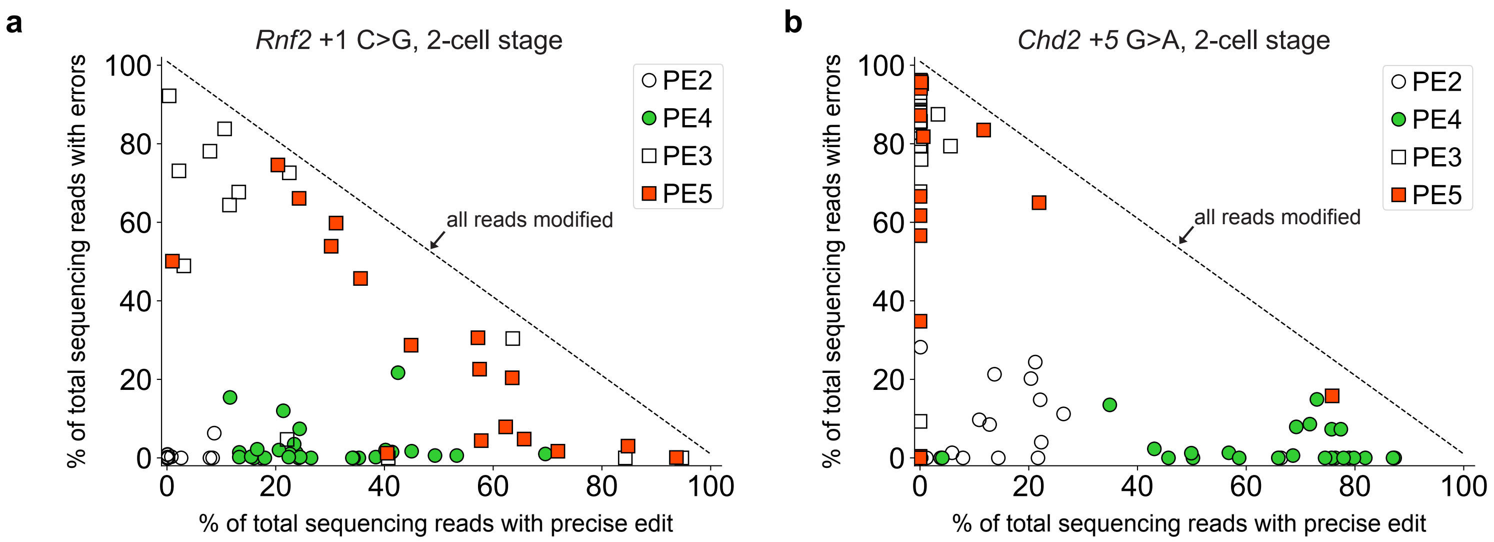
a
Rnf2 +1 C>G, 2-cell stage
% of total sequencing reads with errors
% of total sequencing reads with precise edit
all reads modified
PE2
PE4
PE3
PE5
0
20
40
60
80
100
b
Chd2 +5 G>A, 2-cell stage
% of total sequencing reads with errors
% of total sequencing reads with precise edit
all reads modified
PE2
PE4
PE3
PE5

Supplementary Figure 6. Comparison of editing outcome frequencies across prime editing methods in embryos edited at the two-cell stage. a, Percentages of total reads with the precise *Rnf2* +1 C>G edit (x-axis) verses errors (y-axis) for blastocyst embryos edited at the two-cell stage and sequenced at the blastocyst stage. **b,** Same as (a) but for embryos with the *Chd2* +5 G>A edit. Editing results are compiled from multiple experiments (Supplementary Tables 7-8, Methods) and represent the same datasets as illustrated in Figure 1b.

a

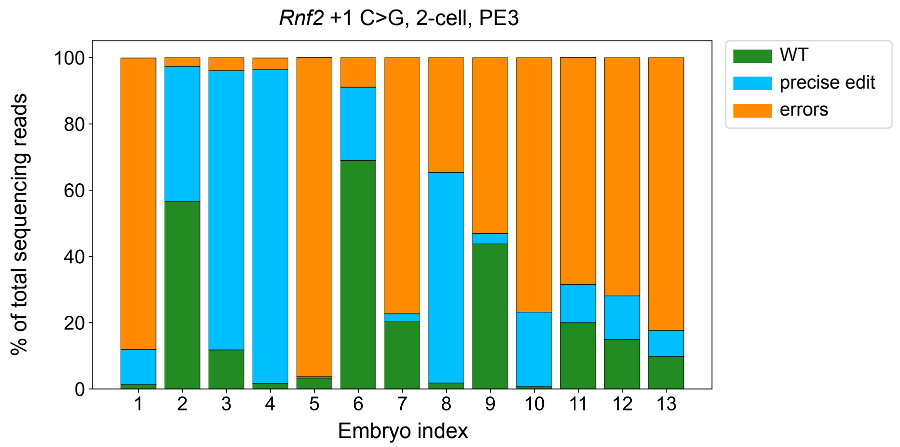


```
                                                                                                                             Allele frequency
                                                                                                                             amongst reads
Ref ACAGCAGGATGTATTATATTACCTGAGGTGTTCGTTGTAACTCATACAAACTGAGTTCCCATGTTTTGCTTAATGGTTGAGTTCCATTTGTCTGCACAGCCTGAGACATTTCTAGGAATAAAA with errors

 1  ---------------------------------------------------------------------------------------------TGCACAGCCTGAGACATTTCTAGGAATAAAA  41%
 2  ACAGCAGGATGTATTATATTACGTGAGGTGTTCGTTGTAACTCATACAAACTGAGTTCCCATGTTTTGCTTAATGGTTGAGTTCCATTTGTCTGTACAGCCTGAGACATTTCTAGGAATAAAA   4%
 3  ACAGCAGGATGTATTATATTACGTGAGGTGTTCGTCGTAACTCATACAAACTGAGTTCCCATGTTTTGCTTAATGGTTGAGTTCCATTTGTCTGCACAGCCTGAGACATTTCTNGGAATAAAA   7%
 4  ACAGCAGGATGTATTATATTACGTGAGGTGTTCGTTG---------------------CCATGTTTTGCTTAATGGTTGAGTTCCA------------GCCTGAGACATTTCTAGGAATAAAA  14%
 5  -------------------------------------------------------------------------------------ATTTGTCTGCACAGCCTGAGACATTTCTAGGAATAAAA  65%
 6  ACAGCAGGATATATTATATTACCTGAGGTGTTCGTTGTAACTCATACAAACTGAGTTCCCATGTTTTGCTTAATGGTTGAGTTCCATTTGTCTGCACAGCCTGAGNCATTTCTAGGAATAAAA  58%
 7  --------------------------------------------------------------------------------------TTTGTCTGCACAGCCTGAGACATTTCTAGGAATAAAA  88%
 8  ACAGCAGGATGTAGACTTT-----------------------------------------------------------------------------------------TTTCTAGGAATAAAA  86%
 9  ACAGCAGGATGTATTATATTACGTGAGGTGTTCGTTTTGTA----------TGAGTT---AAGTTTTGCTTAATGGTTGAGTTCCATTTGTCTGCACAGCCTGAGACATTTCTAGGAATAAAA  80%
10  -------------------------------------------------------------------------------------------------AGCCTGAGACATTTCTAGGAATAAAA  45%
11  ACAGCAGGATGTATTATATTACGTGAGGTGTTCGTTGTAACTCATACAAACTGAGTTCCCATGTTTTGC--------------------------ACAGCCTGAGACATTTCTAGGAATAAAA  47%
12  ---------------------------------------------------------------------------------------------------------------------------  58%
13  ACAGCAGGATGTATTATATTACGTGAGGTGTTCGTTG---------------------------------------------------------CACAGCCTGAGACATTTCTAGGAATAAAA  66%
```

b

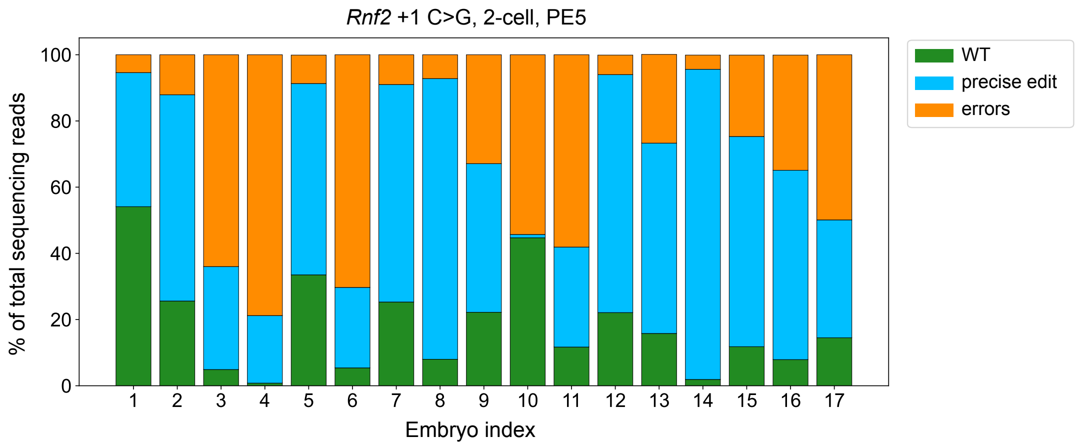


```
                                                                                                                             Allele frequency
                                                                                                                             amongst reads
Ref ACAGCAGGATGTATTATATTACCTGAGGTGTTCGTTGTAACTCATACAAACTGAGTTCCCATGTTTTGCTTAATGGTTGAGTTCCATTTGTCTGCACAGCCTGAGACATTTCTAGGAATAAAA with errors

 1  ACAGCAGGATGTATTATATTACCTGAGGTGTTCGTTGTAACTCATACAAACTGAGTTCCCATGTTTTGCTTAATGGTTGAGTTCCATTTGTCTGCACGGCCTGAGACATTTCTAGGAATAAAA  17%
 2  ACAGCAGGATGTATTATATTACGTGAGGTGTTCGTTGTAACTCATACAAACTGAGTTCCCATGTTTTGCTTAATGGT--------------CTGCACAGCCTGAGACATTTCTAGGAATAAAA  61%
 3  ACAGCAGGATGTATTATATTACGTGAGGTGTTCGTTGTAACTCATACAAACTGAGTTCCCATGTTTTG--------------------------CACAGCCTGAGACATTTCTAGGAATAAAA  78%
 4  ACAGC-----------------------------------------------------------------------------------------------CTGAGACATTTCTAGGAATAAAA  36%
 5  ACAGCAGGATGTATTATATTACGTGAGGTGTTCGTTGTAACTCATACAAACTGAGTTCCCATGTTTTGCTTA--------GTTCCATTTGTCTGCACAGCCTGAGACATTTCTAGGAATAAAA  60%
 6  ACAGCAGGATGTATTATATTACGTGAGGTGTTCGTTGTAACTCATACAAACTGAGTTCCCATGTTTTGCTTAATGGTTG---------------CACAGCCTGAGACATTTCTAGGAATAAAA  53%
 7  ACAGCAGGATGTATTATATTACGTGAGGTGTTCGTTGTAACTCATACAAACTGAGTTCCCATGTTTTGC--------------------------ACAGCCTGAGACATTTCTAGGAATAAAA  49%
 8  ACAGCAGGATGTATTATATTACGTGAGGTGTTCGTTGCAACTTTGTAACTCATACAAACTGAGTTCCCATGTTTTGCTTAATGGTTGAGTTCCATTTGTCTGCACAGCCTGAGACATTTCTAG  16%
 9  ACAGCAGGATGTATTATATTACGTGAGGTGTTCGTTGTAACTCATACAAACTGAGTTCCCATGTTTTGCTTAAT-----------------TTGCACAGCCTGAGACATTTCTAGGAATAAAA  78%
10  ACAGCAGGATGTATTATATTACGTGAGGTGTTCGTTGTAACTCATACAAACTGAGTTCCCATGTTTTGC--------------------------ACAGCCTGAGACATTTCTAGGAATAAAA  44%
11  ACAGCAGGATGTATTATATTACGTGAGGTGTTCGTTGTAACTCATACAAACTGAGTTCCCATGTTTTGCTTAATGGT----------------------------------------------  61%
12  ACAGCAGGATGTATTATATTACGTGAGGTGTTCGTTGTAACTCATACAA--------------------------------------------------------------------------  30%
13  ACAGCAGGATGTATTATATTACGTGAGGTGTTCGTTGTAACTCATACAAACTGAGTTCCCATGTTT-------------------------CTGCACAGCCTGAGACATTTCTAGGAATAAAA  40%
14  ACAGCAGGATGTATTATATTACGTGAGGTGTTCGTTGTAACTCATACAAACTGAGTTCCCATGTTTTGCTTAATGGTTGAGTTCCATTTGTCTACACAGCCTGAGACATTTCTAGGAATAAAA   6%
15  ACAGCAGGATGTATTATATTACGTGAGGTGTTCGTTGTAACTCATACAAACTGAGTTCCCATGTTTTGCTTAATGGTTGAGTT---------TGCACAGCCTGAGACATTTCTAGGAATAAAA  26%
16  ACAGCAGGATGTATTATATTACGTGAGGTGTTCGTTGTAACTCATACAAACTGAGTTCCCATGTTTTGC--------------------------ACAGCCTGAGACATTTCTAGGAATAAAA  55%
17  ACAGCAGGATGTATTATATTACGT-------------------------------------------------------------------CTGCACAGCCTGAGACATTTCTAGGAATAAAA  49%
```

Supplementary Figure 7. Unintended byproducts generated at the *Rnf2* target site when editing with PE3 and PE5 at the two-cell stage. a, Top) Percentages of outcome-classified reads from individual embryos microinjected with PE3 editing components at the two-cell stage. For each embryo, the programmed edit was a +1 C>G substitution in *Rnf2*. Bottom) Aligned sequence and percentage of the most common byproduct observed in each embryo. Percentages calculated from all reads with an unintended modification. Bolded green letters indicate the precise edit. Bolded red letters indicate errors at the target site. Dashed lines represent deletions. **b,** Same as (a) but for editing performed with PE5 components. Editing results are compiled from multiple experiments (Supplementary Table 8, Methods) and represent the same datasets as illustrated in Figure 1b.

a

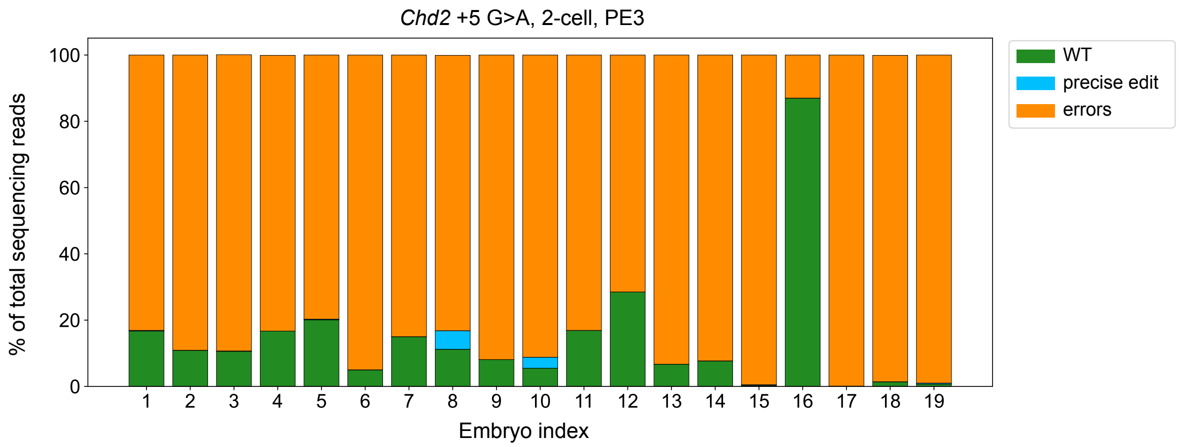


```
                                                                                                                                  Allele frequency
                                                                                                                                  amongst reads
Ref TCTGGACATGTTGTTAGGGCGGTAGCTCCCAGAACGGTGGGCATCCATATGCCTCCGGTCACCATAGTGGTGGTCCTTATACCAATGCTGCTCATACTGATGGTGCCGGTCGCCCCCCCAGGG    with errors

 1  TCTGGACATGTTGTTAGGGCGGTAGCTCCCAGAACGGTGAGCATCCATATGCCTCCGGTCACCATAGTGGTG------------------CTCATACTGATGGTGCCGGTCGCCCCCCCAGGG   42%
 2  TCTGGACATGTTGTTAGGGCGGTAGCTCCCAGAACGGTG---------------------------------------------------CTCATACTGATGGNGCCGGTCGCCCCCCCAGGG   55%
 3  TCTGGACATGTTGTTAGGGCGGTAGCTCCCAGAACGGTGAGCATCGCACCGACTCGGTGCCACTTTTTCAAGNTGATAACGGACTAGCCTCATACTGATG...                        28%
 4  TCTGGACATGTTGTTAGGGCGGTAGC---------------------------------------------------------------------------------------CCCCCCAGGG   39%
 5  --------------------------------------------------------------------------------------------CATACTGATGGTGCCGGTCGCCCCCCCAGGG   46%
 6  TCTGGACATGTTGTTAGGGCGGTAGCTCCCAGAACGGTGA-----------------------------------------------------------------------------------   32%
 7  TCTGGACATGTTGTTAGGGCGGTAGCTCCCAG-----------------------------------------------------------------------------------------GG   33%
 8  TCTGGACATGTTGTTAGGGCGGTAGCTCCCAGAACGGTGAGCATC------------------------------------------------------------------------------   30%
 9  -------------------------------------------------------------------------------------------TCATACTGATGGTGCCGGTCGCCCCCCCAGGG   48%
10  ------------------------------------------------------------ACCATAGTGGTGGTCCTTATAC---------TCATACTGATGGTGCCGGTCGCCCCCCCAGGG   56%
11  TCTGGACATGTTGTTAGGGCGGTAGCTCCCAGAAC---------------------------------------------------------CATACTGATGGTGCCGGTCGCCCCCCCAGGG   48%
12  TCTGGACATGTTGTTAGGGCGGTAGCTCCCAGAAC-----------------------------------------------------TGCTCATACTGATGGTGCCGGTCGCCCCCCCAGGG   54%
13  ----------------------------------------------------------------------------------------------------TGGTGCCGGTCGCCCCCCCAGGG   24%
14  ----------------------------------------------------------------------------------------TGCTCATACTGATGGTGCCGGTCGCCCCCCCAGGG   48%
15  TCTGGACATGTTGTTAGGGCGGTAGCTCCCAG-----------------------------------------------------------------------------------------GG   37%
16  TCTGGACATGTTGTTAGGGCGGTAGCTCCCAGAACGGTGGGCATCCGTTGATGAACG-----------------------------GCTGCTCATACTGATGGTGCCGGTCGCCCCCCCAGGG   58%
17  TCTGGACATGTTGTTAGGGCGGTAGCTCCCAGAACGGTGAGCATC-----------------------------------------GCTGCTCATACTGATGGTGCCGGTCGCCCCCCCAGGG   91%
18  TCTGGACATGTTGTTAGGGCGGTAGCTCCCAGAACGGTATA---------------------------------------------------------------------------CCCAGGG   31%
19  TCTGGAC---------------------------------------------------------------------------------TGCTCATACTGATGGTGCCGGTCGCCCCCCCAGGG   45%
```

b

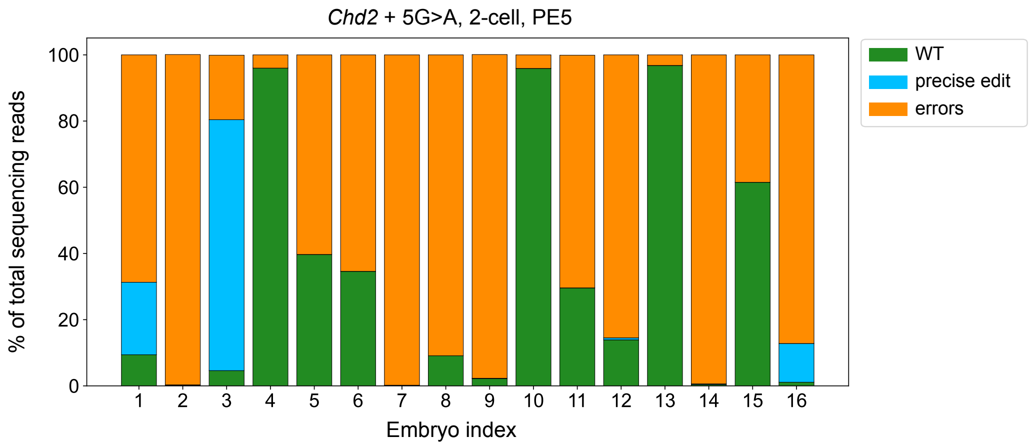


```
                                                                                                                                  Allele frequency
                                                                                                                                  amongst reads
Ref TCTGGACATGTTGTTAGGGCGGTAGCTCCCAGAACGGTGGGCATCCATATGCCTCCGGTCACCATAGTGGTGGTCCTTATACCAATGCTGCTCATACTGATGGTGCCGGTCGCCCCCCCAGGG    with errors

 1  TCTGGACATGTTGTTAGGGCGGTAGCTCCCAGAACGGTGAGCATGAG-----------------------------------------------------------------------CAGGG   46%
 2  TCTGGACATGTTGTTAGGGCGGTAGCTCCCAGAACA-----------------------------------------------------GCTCATACTGATGGTGCCGGTCGCCCCCCCAGGG   30%
 3  TCTGGACATGTTGTTAGGGCGGTA----------------------------------------------------------------TGCTCATACTGATGGTGCCGGTCGCCCCCCCAGGG   40%
 4  TCTGGACATGTTGTTAGGGCGGTAGCTCCCAGAACGGTGGGCATCCATATGCTTCCGGTCACCATAGTGGTGGTCCTTATACCAATGCTGCTCATACTGATGGTGCCGGTCGCCCCCCCAGGG    8%
 5  TCTGGACATGTTGTTAGGGCGGTAGCT------------------------------------------------------------------------------------------------   53%
 6  TCTGGACATGTTGTTAGGGCGGTAGCTCCCAGAACGGTGA-----------------------------------------------------------------------------------   44%
 7  TCTGGACATGTTGTTAGGGCGGTAGCTCCCAGAACGGTGAAATATGA------------------------------------------GCTCATACTGATGGTGCCGGTCGCCCCCCCAGGG   36%
 8  ----------------------------------------------------------------------------------------TGCTCATACTGATGGTGCCGGTCGCCCCCCCAGGG   26%
 9  TCTGGACATGTTGTTAGGGCGGTAGC---------------------------------------------------------------------------------------CCCCCCAGGG   32%
10  TCTGGACATGTTGTTAGGGCGGTAGCTCCCAGAACGGTGGGCATCCATATGCCTCCGGTCACCATAGTGGTGGTCCTTATACCAATGCTGCTCATACTGATGTTGCCGGTCGCCCCCCCAGGG   22%
11  TCTGGACATGTTGTTAGGGCGGTAGCTCCCAGAACGGT---------------------CACCATAGTGGTGGTCCTTATACCAATGCTGCTCATACTGATGGTGCCGGTCGCCCCCCCAGGG   23%
12  TCTGGACATGTTGTTAGGGCGGTAGCTCCCAGAACGGTGAG[CATCCATAT]2GCCTCCGGTCACTC-----------------------------------------------------------   30%
13  TCTGGACATGTTGTTAGGGCGGTAGCTCCCAGAATGGTGGGCATCCATATGCCTCCGGTCACCATAGTGGTGGTCCTTATACCAATGCTGCTCATACTGATGGTGCCGGTCGCCCCCCCAGGG    7%
14  ----------------------------------------------------------------------------------CAATGCTGCTCATACTGATGGTGCCGGTCGCCCCCCCAGGG   24%
15  TCTGGACATGTTGTTAGGGCGGTAGCTC-----------------------------------------------------------------ATACTGATGGTGCCGGTCGCCCCCCCAGGG   71%
16  TCTGGACATGTTGTTAGGGCGGTAGCTCCCAGAACGGTGAGCATC--------------------------------------------GCTCATACTGATGGTGCCGGTCGCCCCCCCAGGG   33%
```

Supplementary Figure 8. Unintended byproducts generated at the *Chd2* target site when editing with PE3 and PE5 at the two-cell stage. a, Top) Percentages of outcome-classified reads from individual embryos microinjected with PE3 editing components at the two-cell stage. For each embryo, the programmed edit was a +5 G>A substitution in *Chd2*. Bottom) Aligned sequence and percentage of the most common byproduct observed in each embryo. Percentages calculated from all reads with an unintended modification. Bolded green letters indicate the precise edit. Bolded red letters indicate errors at the target site. Dashed lines represent deletions. **b,** Same as (a) but for editing performed with PE5 components. Editing results are compiled from multiple experiments (Supplementary Table 8, Methods) and represent the same datasets as illustrated in Figure 1b.

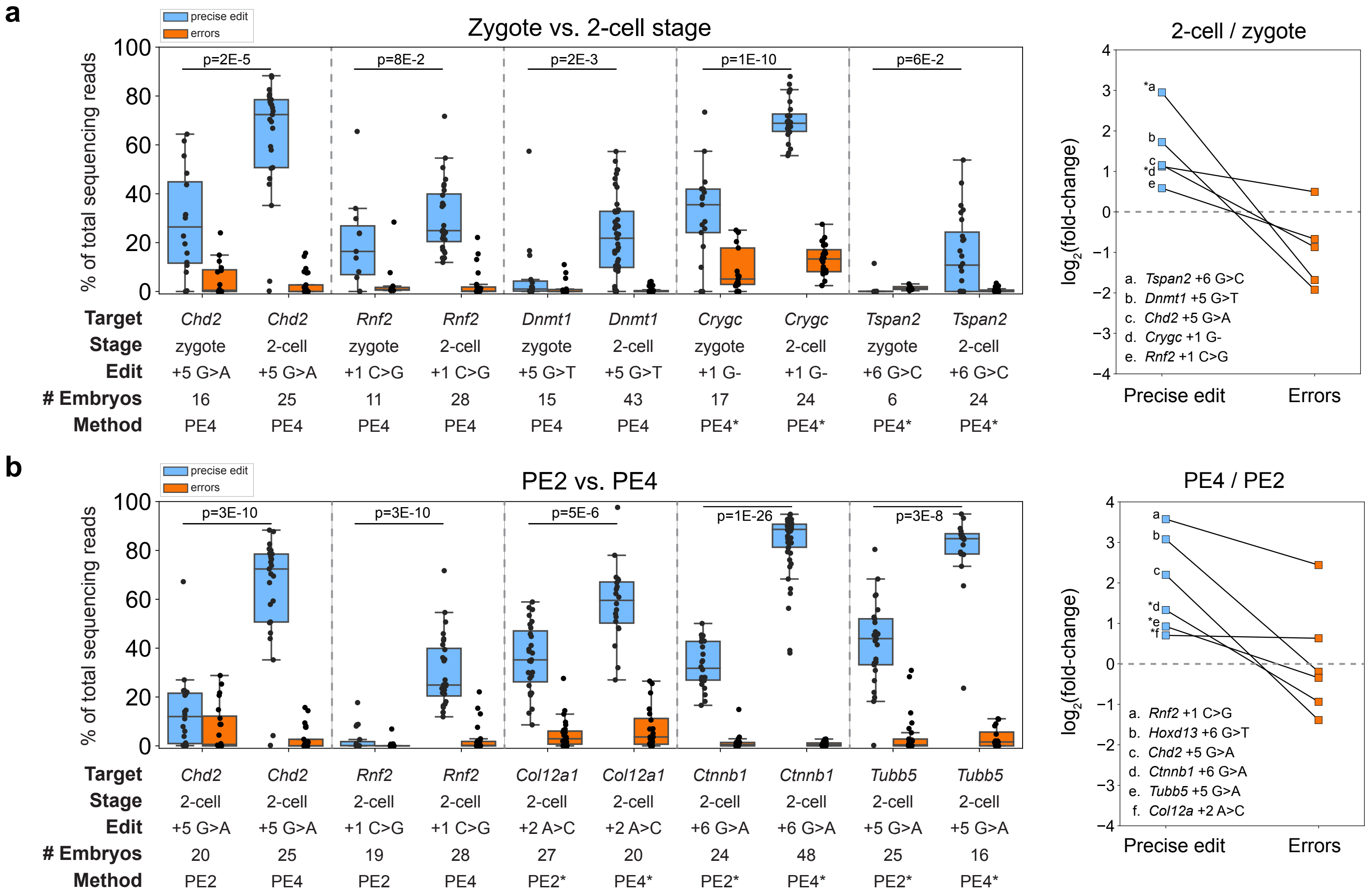
a
precise edit
errors
Zygote vs. 2-cell stage
% of total sequencing reads
p=2E-5
p=8E-2
p=2E-3
p=1E-10
p=6E-2
Target: Chd2, Chd2, Rnf2, Rnf2, Dnmt1, Dnmt1, Crygc, Crygc, Tspan2, Tspan2
Stage: zygote, 2-cell, zygote, 2-cell, zygote, 2-cell, zygote, 2-cell, zygote, 2-cell
Edit: +5 G>A, +5 G>A, +1 C>G, +1 C>G, +5 G>T, +5 G>T, +1 G-, +1 G-, +6 G>C, +6 G>C
Embryos: 16, 25, 11, 28, 15, 43, 17, 24, 6, 24
Method: PE4, PE4, PE4, PE4, PE4, PE4, PE4*, PE4*, PE4*, PE4*
2-cell / zygote
log2(fold-change)
a. Tspan2 +6 G>C
b. Dnmt1 +5 G>T
c. Chd2 +5 G>A
d. Crygc +1 G-
e. Rnf2 +1 C>G
Precise edit
Errors
b
precise edit
errors
PE2 vs. PE4
% of total sequencing reads
p=3E-10
p=3E-10
p=5E-6
p=1E-26
p=3E-8
Target: Chd2, Chd2, Rnf2, Rnf2, Col12a1, Col12a1, Ctnnb1, Ctnnb1, Tubb5, Tubb5
Stage: 2-cell, 2-cell, 2-cell, 2-cell, 2-cell, 2-cell, 2-cell, 2-cell, 2-cell, 2-cell
Edit: +5 G>A, +5 G>A, +1 C>G, +1 C>G, +2 A>C, +2 A>C, +6 G>A, +6 G>A, +5 G>A, +5 G>A
Embryos: 20, 25, 19, 28, 27, 20, 24, 48, 25, 16
Method: PE2, PE4, PE2, PE4, PE2*, PE4*, PE2*, PE4*, PE2*, PE4*
PE4 / PE2
log2(fold-change)
a. Rnf2 +1 C>G
b. Hoxd13 +6 G>T
c. Chd2 +5 G>A
d. Ctnnb1 +6 G>A
e. Tubb5 +5 G>A
f. Col12a +2 A>C
Precise edit
Errors

Supplementary Figure 9. Optimization of prime editing conditions in the early mouse embryo. a, Left) Percentages of total reads containing the precise edit (blue) or errors (orange) for *Chd2* +5 G>A, *Rnf2* +1 C>G, *Dnmt1* +5 G>T, *Crygc* +1 G- (deletion) and *Tspan2* +6 G>C edits. Each datapoint represents an individual embryo. Group size, edit, prime editing method, and stage of microinjection indicated. Right) Log2(fold-change) of the average precise edit frequency (blue) or average error frequency (orange) observed in embryos microinjected with PE4 components (editor mRNA, pegRNA, mMLH1dn mRNA) at the two-cell stage versus zygote stage across different edits. Black lines connect the fold change in precise edit and error frequencies for the same edit/comparison. Asterisks specify edits performed using the optimized PEmax editor (as opposed to the PE2 editor). Dashed grey line indicates no change between conditions. **b,** Left) Percentages of total reads containing the precise edit (blue) or errors (orange) for *Chd2* +5 G>A, *Rnf2* +1 C>G, *Col12a1* +2 A>C, *Ctnnb1* +6 G>A and *Tubb5* +5 G>A edits. Each datapoint represents an individual embryo. Group size, edit, prime editing method, and stage of microinjection indicated. Right) Log2(fold-change) of the average precise editing frequency (blue) or average error frequency (orange) observed in embryos microinjected at the two-cell stage with PE4 components (editor mRNA, pegRNA, mMLH1dn mRNA) versus PE2 components (editor mRNA, pegRNA) across different edits. Black lines connect the change in precise edit and error frequencies for the same edit/comparison. Asterisks specify edits performed using the PEmax editor (as opposed to the PE2 editor). Dashed grey line indicates no change between conditions. Editing results are compiled from multiple experiments (Supplementary Tables 9, Methods) and represent the same datasets as illustrated in Figure 1a-f. P-values from two-sided Student's t-tests. For box plots, boxes indicate the median and interquartile range (IQR) of each group with whiskers extending 1.5*IQR past the upper and lower quartiles.

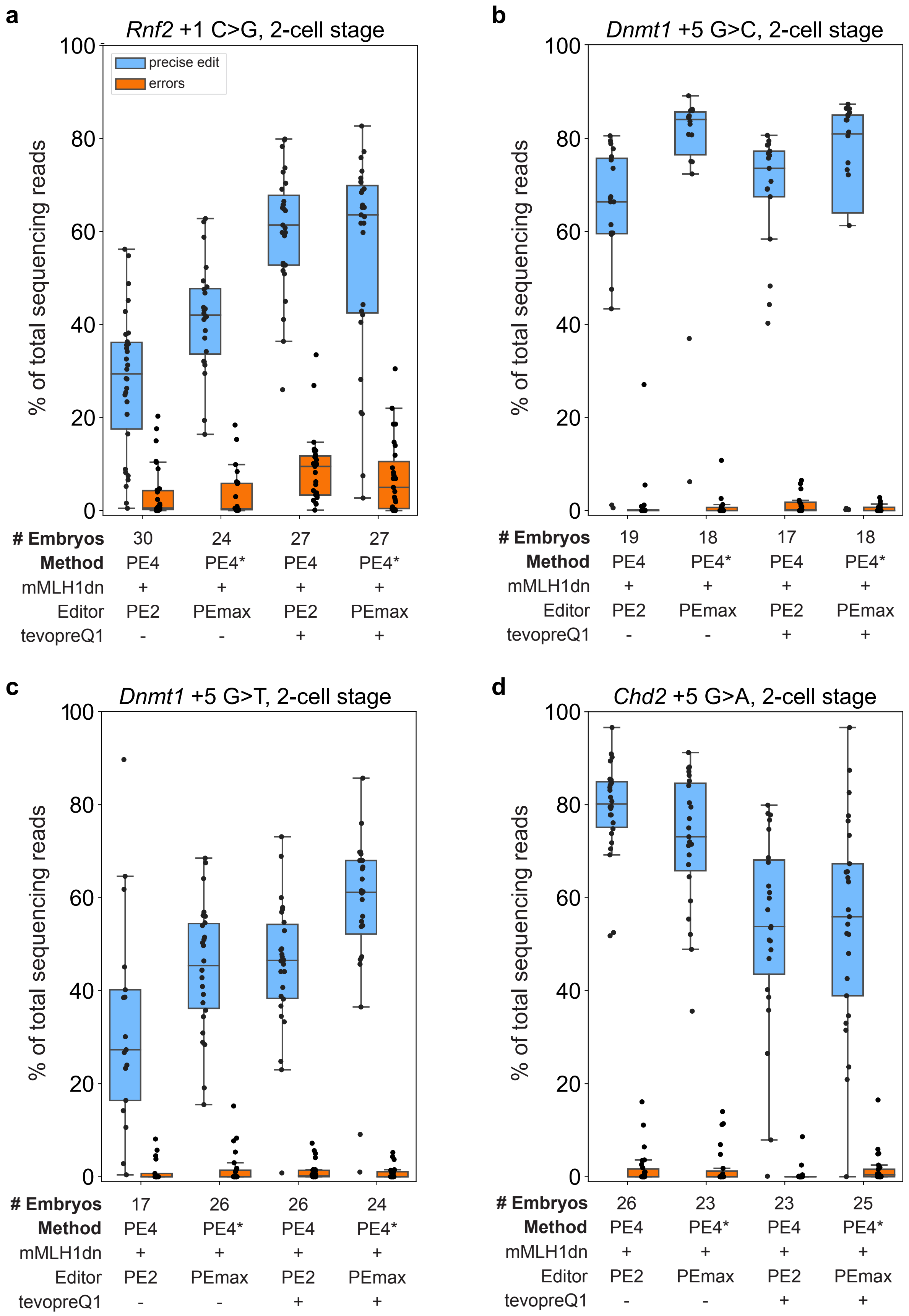

a
Rnf2 +1 C>G, 2-cell stage
precise edit
errors
% of total sequencing reads
0
20
40
60
80
100
Embryos 30 24 27 27
Method PE4 PE4* PE4 PE4*
mMLH1dn + + + +
Editor PE2 PEmax PE2 PEmax
tevopreQ1 - - + +
b
Dnmt1 +5 G>C, 2-cell stage
% of total sequencing reads
# Embryos 19 18 17 18
Method PE4 PE4* PE4 PE4*
mMLH1dn + + + +
Editor PE2 PEmax PE2 PEmax
tevopreQ1 - - + +
c
Dnmt1 +5 G>T, 2-cell stage
% of total sequencing reads
# Embryos 17 26 26 24
Method PE4 PE4* PE4 PE4*
mMLH1dn + + + +
Editor PE2 PEmax PE2 PEmax
tevopreQ1 - - + +
d
Chd2 +5 G>A, 2-cell stage
% of total sequencing reads
# Embryos 26 23 23 25
Method PE4 PE4* PE4 PE4*
mMLH1dn + + + +
Editor PE2 PEmax PE2 PEmax
tevopreQ1 - - + +

Supplementary Figure 10. Testing optimized prime editing components in embryos. a, Percentages of total reads containing the precise edit (blue) or errors (orange) from embryos microinjected with PE4 editing components (indicated editor mRNA, pegRNA, mMLH1dn mRNA) targeting *Rnf2* +1 C>G edit at the two-cell stage. Each datapoint represents an individual embryo. “tevopreQ1” indicates the engineered pegRNA (epegRNA) architecture from Nelson and colleagues[33] which incorporates a structured RNA motif (evopreQ$_1$-1 trimmed) at the 3' terminus for enhanced RNA stability. **b,** same as (a) but for *Dnmt1* +5 G>C edit. **c,** same as (a) but for *Dnmt1* +5 G>T edit. **d,** same as (a) but for *Chd2* +5 G>A edit. We note that while epegRNA data suggests that PEmbryo is compatible with modified guides, exact interpretation is difficult because addition of the 3′ motif increased the length of each RNA past the supported maximum for quality ensured commercial synthesis. Data are compiled from multiple experiments (Supplementary Table 10, Methods). PE4* denotes the PE4 editing method using the optimized PEmax editor. Box plots indicate the median and interquartile range (IQR) of each group with whiskers extending 1.5*IQR past the upper and lower quartiles.

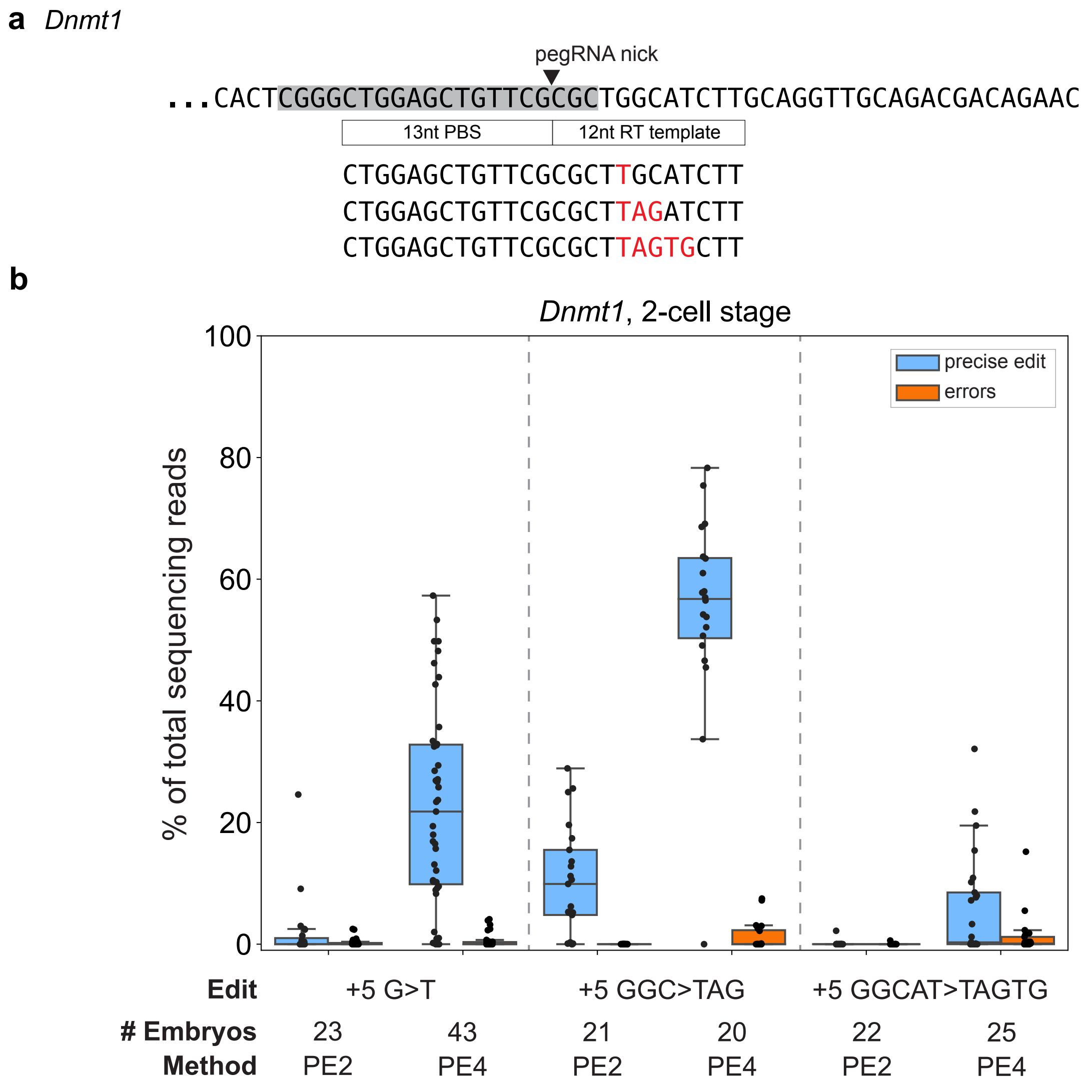
a Dnmt1
pegRNA nick
...CACTCGGGCTGGAGCTGTTCGCGCTGGCATCTTGCAGGTTGCAGACGACAGAAC...
13nt PBS
12nt RT template
CTGGAGCTGTTCGCGCTTGCATCTT
CTGGAGCTGTTCGCGCTTAGATCTT
CTGGAGCTGTTCGCGCTTAGTGCTT
b
Dnmt1, 2-cell stage
% of total sequencing reads
100
80
60
40
20
0
precise edit
errors
Edit
+5 G>T
+5 GGC>TAG
+5 GGCAT>TAGTG
Embryos
23
43
21
20
22
25
Method
PE2
PE4
PE2
PE4
PE2
PE4

Supplementary Figure 11. Effects of mMLH1dn on prime editing efficiency for edits with contiguous substitutions in embryos. a, Schematic of *Dnmt1* target site and pegRNA designs which hold the primer binding site (PBS) and RT template lengths constant while varying the number of consecutive base substitutions encoded in the template. **b,** Percentages of reads containing precise edits (blue) or errors (orange) from embryos microinjected with PE2 components (PE2 editor mRNA, pegRNA) or PE4 components (PE2 editor mRNA, pegRNA, mMLH1dn mRNA) at the two-cell stage. Editing results are compiled from multiple experiments (Supplementary Table 11, Methods) and include the same *Dnmt1* +5 G>T datasets as analyzed in Figure 1c,e-f. Box plots indicate the median and interquartile range (IQR) of each group with whiskers extending 1.5*IQR past the upper and lower quartiles.

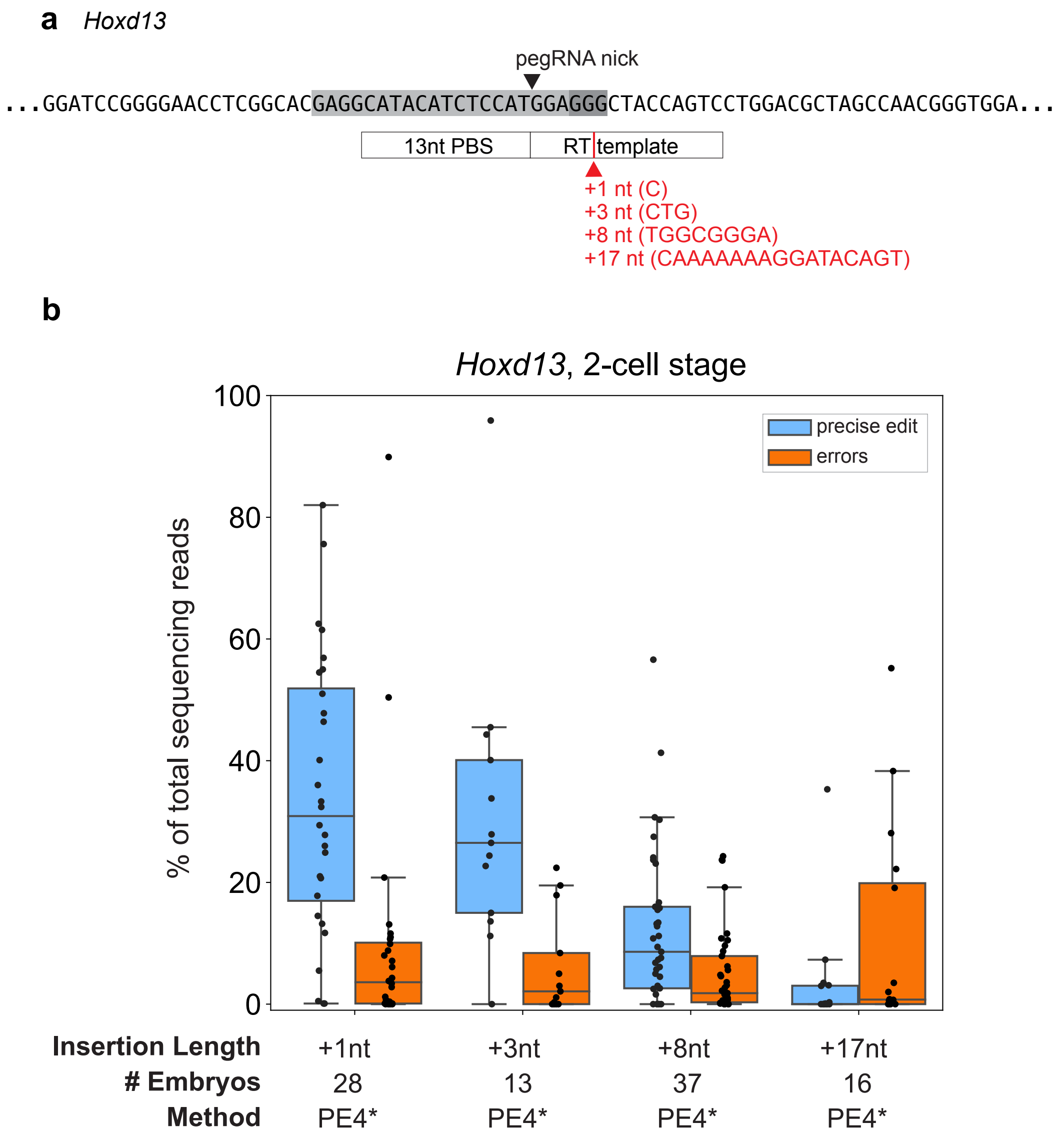
a
Hoxd13
pegRNA nick
...GGATCCGGGGAACCTCGGCACGAGGCATACATCTCCATGGAGGGCTACCAGTCCTGGACGCTAGCCAACGGGTGGA...
13nt PBS
RT template
+1 nt (C)
+3 nt (CTG)
+8 nt (TGGCGGGA)
+17 nt (CAAAAAAAGGATACAGT)
b
Hoxd13, 2-cell stage
% of total sequencing reads
precise edit
errors
Insertion Length
Embryos
Method
+1nt
28
PE4*
+3nt
13
PE4*
+8nt
37
PE4*
+17nt
16
PE4*

Supplementary Figure 12. Editing efficiencies for insertions of different lengths in *Hoxd13* with PEmbryo. a, Schematic of *Hoxd13* target site and pegRNA designs which hold the edit position, primer binding site (PBS), and 3' homology region within the RT template constant while varying the insertion length of the encoded edit. **b,** Percentages of total reads containing the precise edit (blue) or errors (orange) in embryos microinjected with PE4* components (PEmax editor mRNA, pegRNA, mMLH1dn mRNA) at the two-cell stage. Editing results include the *Hoxd13* +6 insertion datasets depicted in Figure 1f. Box plots indicate the median and interquartile range (IQR) of each group with whiskers extending 1.5*IQR past the upper and lower quartiles.

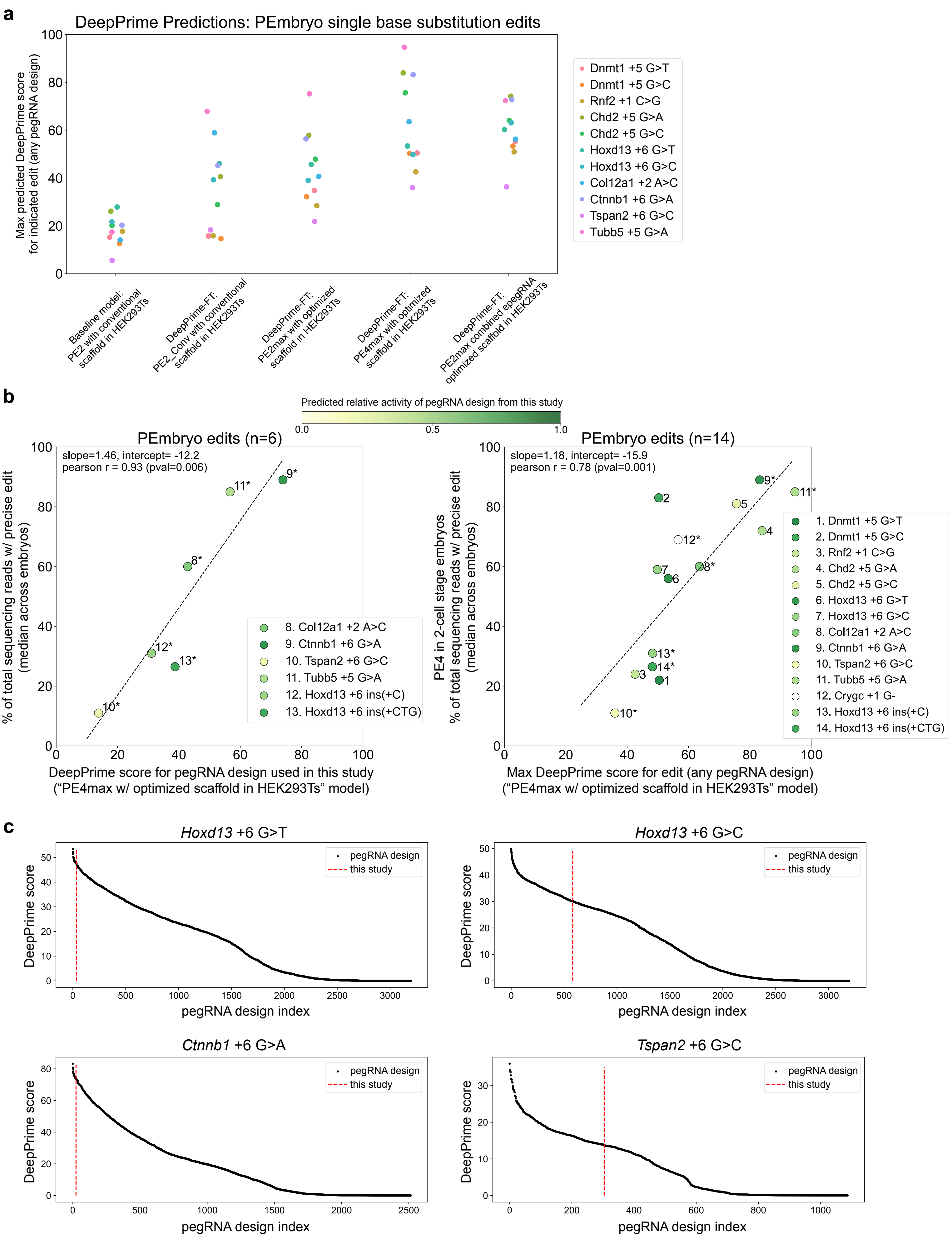
a
DeepPrime Predictions: PEmbryo single base substitution edits
Max predicted DeepPrime score
for indicated edit (any pegRNA design)
Baseline model:
PE2 with conventional
scaffold in HEK293Ts
DeepPrime-FT:
PE2_Conv with conventional
scaffold in HEK293Ts
DeepPrime-FT:
PE2max with optimized
scaffold in HEK293Ts
DeepPrime-FT:
PE4max with optimized
scaffold in HEK293Ts
DeepPrime-FT:
PE2max combined epegRNA
optimized scaffold in HEK293Ts
Dnmt1 +5 G>T
Dnmt1 +5 G>C
Rnf2 +1 C>G
Chd2 +5 G>A
Chd2 +5 G>C
Hoxd13 +6 G>T
Hoxd13 +6 G>C
Col12a1 +2 A>C
Ctnnb1 +6 G>A
Tspan2 +6 G>C
Tubb5 +5 G>A
b
Predicted relative activity of pegRNA design from this study
PEmbryo edits (n=6)
slope=1.46, intercept= -12.2
pearson r = 0.93 (pval=0.006)
PE4 in 2-cell stage embryos
% of total sequencing reads w/ precise edit
(median across embryos)
8. Col12a1 +2 A>C
9. Ctnnb1 +6 G>A
10. Tspan2 +6 G>C
11. Tubb5 +5 G>A
12. Hoxd13 +6 ins(+C)
13. Hoxd13 +6 ins(+CTG)
DeepPrime score for pegRNA design used in this study
(“PE4max w/ optimized scaffold in HEK293Ts” model)
PEmbryo edits (n=14)
slope=1.18, intercept= -15.9
pearson r = 0.78 (pval=0.001)
1. Dnmt1 +5 G>T
2. Dnmt1 +5 G>C
3. Rnf2 +1 C>G
4. Chd2 +5 G>A
5. Chd2 +5 G>C
6. Hoxd13 +6 G>T
7. Hoxd13 +6 G>C
8. Col12a1 +2 A>C
9. Ctnnb1 +6 G>A
10. Tspan2 +6 G>C
11. Tubb5 +5 G>A
12. Crygc +1 G-
13. Hoxd13 +6 ins(+C)
14. Hoxd13 +6 ins(+CTG)
Max DeepPrime score for edit (any pegRNA design)
(“PE4max w/ optimized scaffold in HEK293Ts” model)
c
Hoxd13 +6 G>T
Hoxd13 +6 G>C
Ctnnb1 +6 G>A
Tspan2 +6 G>C
DeepPrime score
pegRNA design index
pegRNA design
this study

Supplementary Figure 13. Comparison of PEmbryo editing efficiencies to predictions from DeepPrime[44]. a, Max predicted editing efficiency (DeepPrime score), irrespective of pegRNA design, for each single base substitution edit considered in our study across the original ("baseline") and finetuned ("DeepPrime-FT") models trained on editing results in HEK293T cells[44]. "PE2max" and "PE4max" methods are denoted as PE2* and PE4* in our study. **b,** Left) Comparison of predicted prime editing efficiencies (DeepPrime score) from the DeepPrime-FT model trained on results from applying PE4max in HEK293Ts to observed prime editing efficiencies (median % of total reads with precise edits across embryos) in mouse embryos microinjected at the two-cell stage with PE4max components (PEmax editor mRNA, pegRNA, mMLH1dn mRNA, denoted as PE4* in our study). Each dot represents a specific edit and pegRNA design used in our study. Color shade indicates the relative score of the pegRNA design compared to the maximum score for a given edit, across all possible pegRNA designs, as predicted with the DeepPrime-FT model (Methods). Right) Same as (left) but including additional PE4 results from our study and using the maximum scores predicted for each given edit by DeepPrime-FT, irrespective of pegRNA design. Note, our Crygc +1 G- pegRNA design was not considered by DeepPrime-FT. **c,** Scores from the "PE4max w/ optimized scaffold in HEK293Ts" DeepPrime-FT model for all possible pegRNA designs enabling the indicated edit using default model parameters (Methods). Rank and score for designs used in this study are indicated by the dashed red line. Editing results from mouse embryos are compiled from multiple experiments (Supplementary Table 9, Methods) and represent the same datasets as illustrated in Figure 1b-f and Supplementary Figure 9a-b.

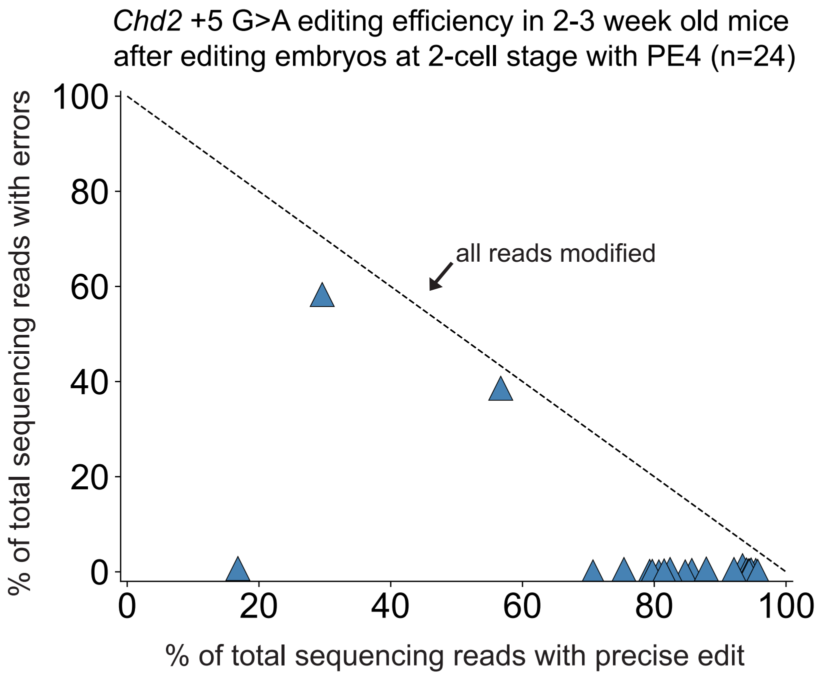
Chd2 +5 G>A editing efficiency in 2-3 week old mice
after editing embryos at 2-cell stage with PE4 (n=24)
% of total sequencing reads with errors
% of total sequencing reads with precise edit
all reads modified
0
20
40
60
80
100

Supplementary Figure 14. *Chd2* editing outcome frequencies in PEmbryo edited mice. Percentages of total reads with the precise edit (x-axis) verses errors (y-axis) in 2-3 week old mice developed from embryos microinjected at the two-cell stage with PE4 editing components (PE2 editor mRNA, pegRNA, mMLH1dn mRNA) targeting *Chd2* +5 G>A. Data are compiled from multiple experiments (Supplementary Table 13, Methods) and represent the same results depicted in Figure 2a.

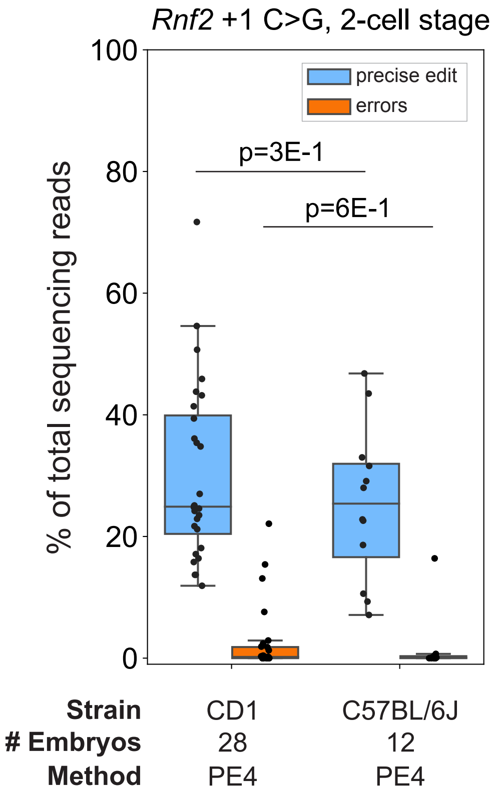

Rnf2 +1 C>G, 2-cell stage
% of total sequencing reads
100
80
60
40
20
0
precise edit
errors
p=3E-1
p=6E-1
Strain
Embryos
Method
CD1
28
PE4
C57BL/6J
12
PE4

Supplementary Figure 15. PEmbryo editing in common mouse strains. Percentages of classified reads observed in CD1 (n=28) and C57BL/6J (n=12) blastocysts from embryos microinjected with PE4 editing components (PE2 editor mRNA, pegRNA, mMLH1dn mRNA) at the two-cell stage. Percentages of total reads containing the precise edit (blue) or errors (orange) were assessed between strains by two-sided Welch's t-test and found to be insignificant (p-value > 0.05). Data are compiled from multiple experiments (Supplementary Table 14, Methods) and results from the CD1 strain are the same as depicted in Figure 1b. Box plots indicate the median and interquartile range (IQR) of each group with whiskers extending 1.5*IQR past the upper and lower quartiles.

PE4 Family: Variants detected in microsatellite regions

ID	Name	Length (bp)	Motif	Parent (M)	Parent (F)	Sibling 1*	Sibling 2*	Sibling 3*	Sibling 4	Sibling 5
MS1	U12235	23	(A)n	2	2	1	0	0	1	0
MS2	Aa003063	22	(A)n	1	0	1	0	0	0	1
MS3	L24372	29	(A)n	1	0	1	1	0	1	0
MS4	D1Mit79	62	(CA)n	1	1	1	1	1	0	1
MS5	D9Mit67	44	(CA)n	0	0	0	1	0	1	0
MS6	D1Mit355	64	(CA)n	1	1	2	0	0	0	0
MS7	D4Mit27	243	(TG)n	0	0	1	0	1	1	0
MS8	D15Mit59	132	(AC)n	0	0	0	0	0	0	0
MS9	D14Mit15	42	(AC)n	0	0	0	0	0	0	0
MS10	D18Mit15	44	(TC)n	0	1	1	1	1	0	1
MS11	D7Mit91	48	(AC)n	0	0	0	0	0	0	0
MS12	D10Mit2	48	(AC)n	0	0	0	0	0	0	0
Gene 1	*Tgfbr2*	87668	multiple	22	26	21	21	21	26	21
Gene 2	*Bax*	6770	multiple	0	0	0	0	0	0	0
		**Total (excluding *Tgfbr2*)**		**6**	**5**	**8**	**4**	**3**	**4**	**3**

Supplementary Figure 16. Assessment of genomic stability at select microsatellite regions. The sum of non-WT alleles detected within each considered microsatellite and genic region for each mouse/embryo comprising the PE4 Family subjected to whole genome sequencing. Asterisks (*) indicate embryos injected with PE4 components targeting the *Chd2* +5 G>A edit at the two-cell stage (see pedigree diagram in Figure 2b). The sum of all detected variants within considered regions per mouse/embryo excluding *Tgfbr2* is reported at the bottom.

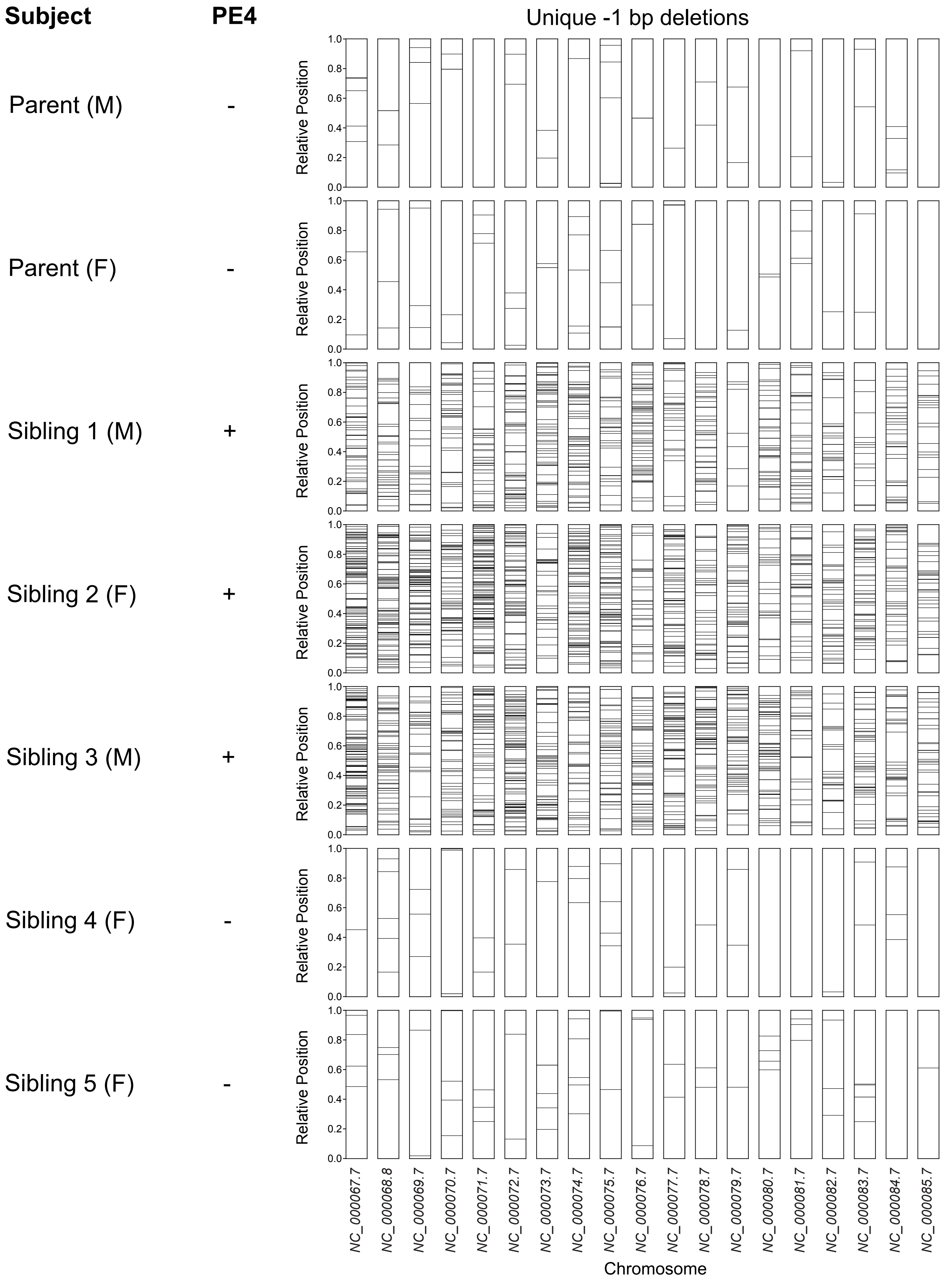

Subject
PE4
Unique -1 bp deletions
Parent (M) -
Parent (F) -
Sibling 1 (M) +
Sibling 2 (F) +
Sibling 3 (M) +
Sibling 4 (F) -
Sibling 5 (F) -
Relative Position
0.0
0.2
0.4
0.6
0.8
1.0
NC_000067.7
NC_000068.8
NC_000069.7
NC_000070.7
NC_000071.7
NC_000072.7
NC_000073.7
NC_000074.7
NC_000075.7
NC_000076.7
NC_000077.7
NC_000078.7
NC_000079.7
NC_000080.7
NC_000081.7
NC_000082.7
NC_000083.7
NC_000084.7
NC_000085.7
Chromosome

Supplementary Figure 17. Genomic distribution of unique -1 bp deletions in samples from PE4 mouse family. Chromosomal positions of unique -1 bp deletions detected in each mouse/embryo from the PE4 Family subjected to whole genome sequencing (see pedigree diagram in Figure 2b). Each vertical bar represents a mouse chromosome, excluding sex chromosomes (n=19), normalized by total size. Horizontal lines represent individual -1 bp deletions unique to the indicated sample (Methods). Embryos microinjected with PE4 components (PE2 editor mRNA, pegRNA, mMLH1dn mRNA) targeting the *Chd2* +5 G>A edit are indicated.

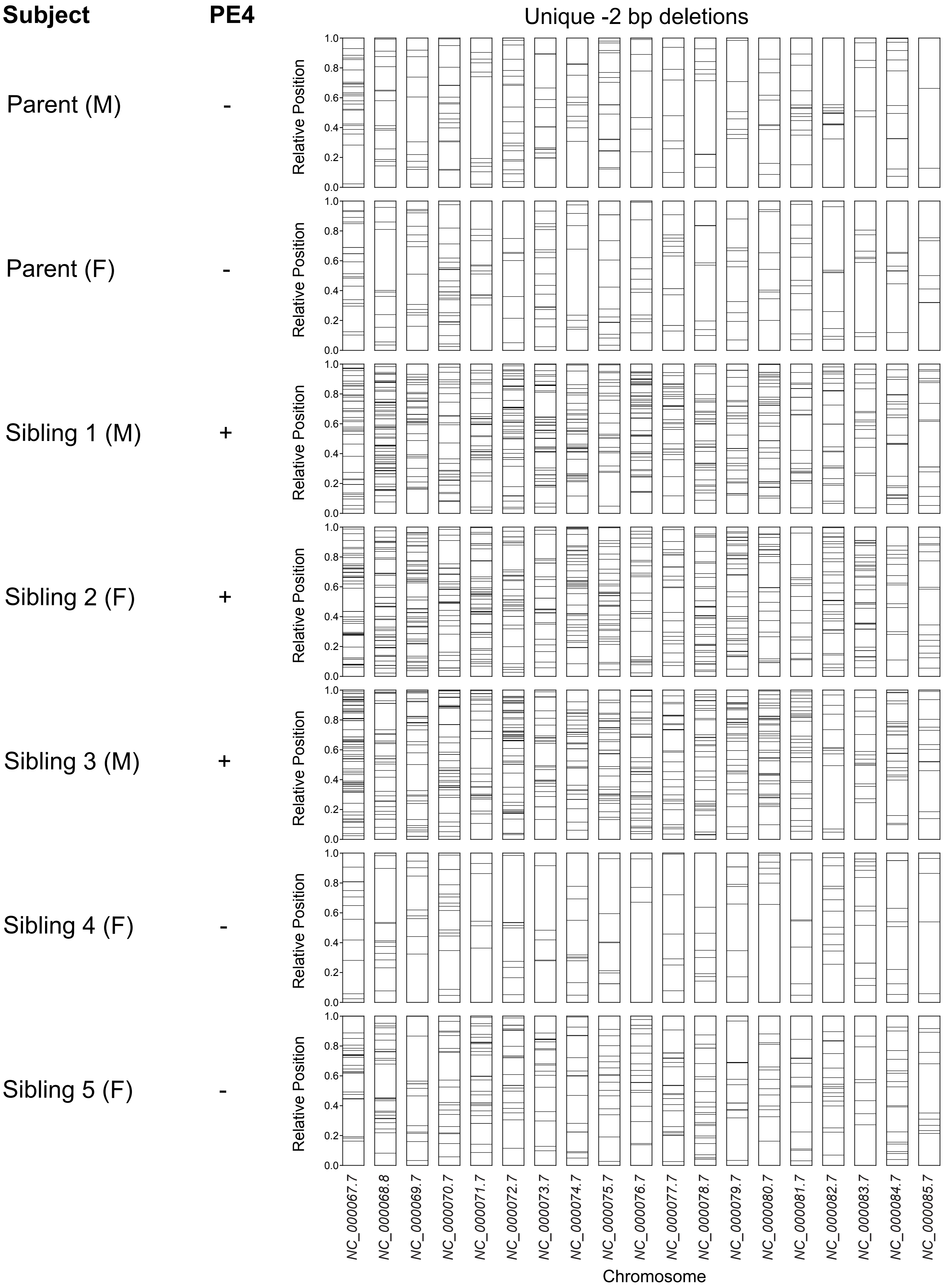

Subject
PE4
Unique -2 bp deletions
Parent (M)
-
Parent (F)
-
Sibling 1 (M)
+
Sibling 2 (F)
+
Sibling 3 (M)
+
Sibling 4 (F)
-
Sibling 5 (F)
-
Relative Position
0.0
0.2
0.4
0.6
0.8
1.0
NC_000067.7
NC_000068.8
NC_000069.7
NC_000070.7
NC_000071.7
NC_000072.7
NC_000073.7
NC_000074.7
NC_000075.7
NC_000076.7
NC_000077.7
NC_000078.7
NC_000079.7
NC_000080.7
NC_000081.7
NC_000082.7
NC_000083.7
NC_000084.7
NC_000085.7
Chromosome

Supplementary Figure 18. Genomic distribution of unique -2 bp deletions in samples from PE4 mouse family. Chromosomal positions of unique -2 bp deletions detected in each mouse/embryo from the PE4 Family subjected to whole genome sequencing (see pedigree diagram in Figure 2b). Each vertical bar represents a mouse chromosome, excluding sex chromosomes (n=19), normalized by total size. Horizontal lines represent individual -2 bp deletions unique to the indicated sample (Methods). Embryos microinjected with PE4 components (PE2 editor mRNA, pegRNA, mMLH1dn mRNA) targeting the *Chd2* +5 G>A edit are indicated.

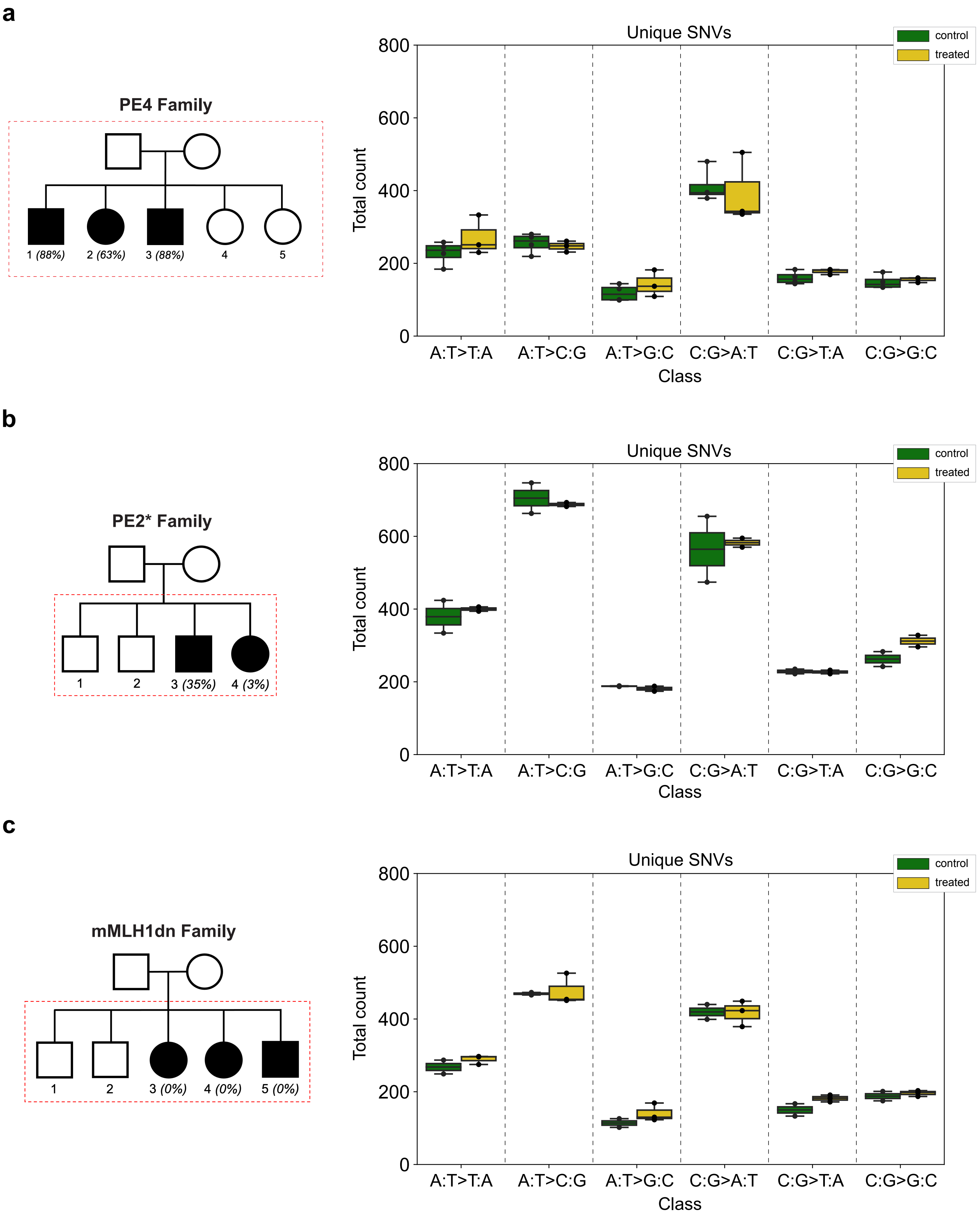
a
PE4 Family
1 (88%)
2 (63%)
3 (88%)
4
5
Unique SNVs
control
treated
Total count
800
600
400
200
0
A:T>T:A
A:T>C:G
A:T>G:C
C:G>A:T
C:G>T:A
C:G>G:C
Class
b
PE2* Family
1
2
3 (35%)
4 (3%)
Unique SNVs
control
treated
Total count
800
600
400
200
0
A:T>T:A
A:T>C:G
A:T>G:C
C:G>A:T
C:G>T:A
C:G>G:C
Class
c
mMLH1dn Family
1
2
3 (0%)
4 (0%)
5 (0%)
Unique SNVs
control
treated
Total count
800
600
400
200
0
A:T>T:A
A:T>C:G
A:T>G:C
C:G>A:T
C:G>T:A
C:G>G:C
Class

Supplementary Figure 19. Number of unique SNVs by type for treated and control samples in mouse families. a, Left) Pedigree of mouse family in which select embryos (black, "treated" group) were microinjected with PE4 components (PE2 editor mRNA, pegRNA, mMLH1dn mRNA) targeting the *Chd2* +5 G>A edit at the two-cell stage. Percentages indicate precise edit efficiency in treated embryos evaluated at E12.5. "Control" group comprised of unshaded mice/embryos from pedigree including both parents and sibling embryos microinjected with PE2 editor mRNA only. Right) Number of unique SNVs with the indicated classification detected in samples from control (n=4 mice/embryos) and treated (n=3 embryos) groups. **b,** Left) Pedigree of mouse family in which select embryos (black, "treated" group) were microinjected with PE2* components (PEmax editor mRNA, *Chd2* +5 G>A pegRNA) at the two-cell stage. Percentages indicate precise edit efficiency (% of total reads) in treated embryos evaluated at E12.5. "Control" group comprised of unshaded sibling embryos from pedigree which were microinjected with pegRNA only. Right) Number of unique SNVs with the indicated classification detected in samples from control (n=2 embryos) and treated (n=2 embryos) groups. **c,** Left) Pedigree of mouse family in which select embryos (black, "treated" group) were microinjected with mMLH1dn mRNA and *Chd2* +5 G>A pegRNA (but no editor) at the two-cell stage. Percentages indicate precise edit efficiency (% of total reads) in treated embryos evaluated at E12.5. "Control" group comprised of unshaded sibling embryos from pedigree which were microinjected with pegRNA only. Right) Number of unique SNVs with the indicated classification detected in samples from control (n=2 embryos) and treated (n=3 embryos) groups. The red dashed box in pedigree diagrams indicates samples subjected to whole genome sequencing. Box plots indicate the median and interquartile range (IQR) of each group with whiskers extending 2.5*IQR past the upper and lower quartiles.

a

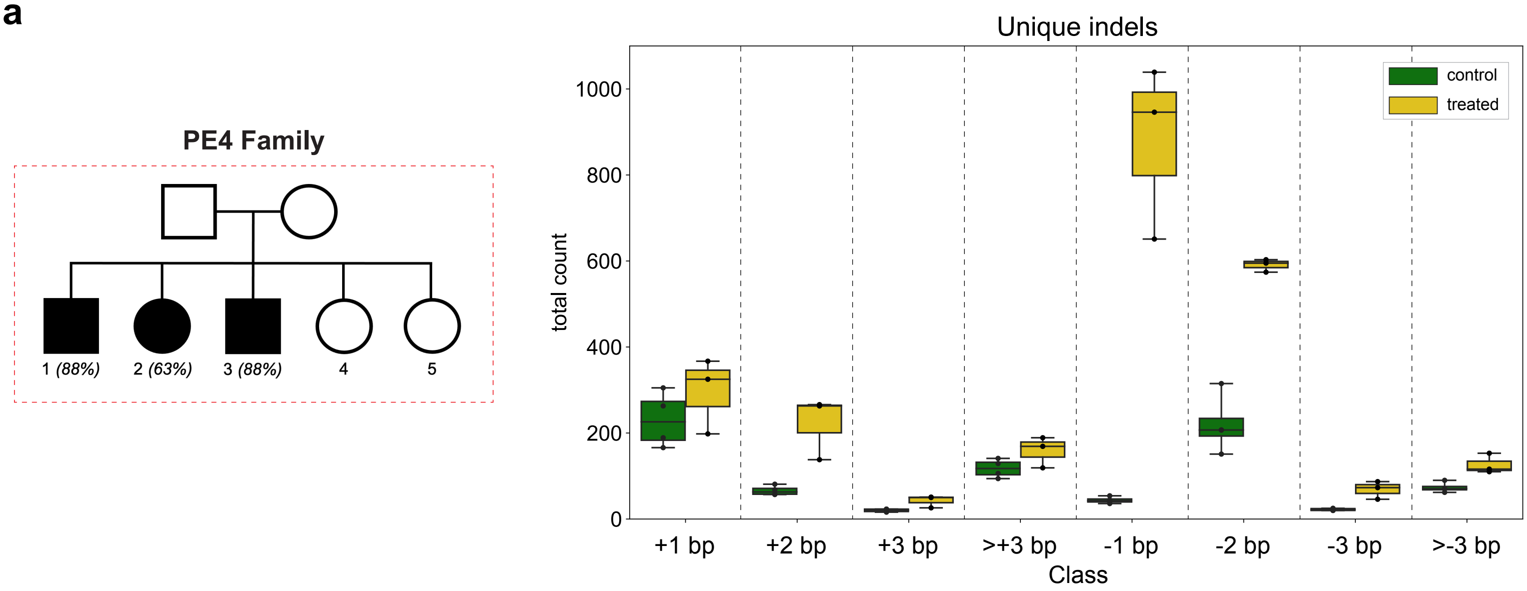
PE4 Family
1 (88%)
2 (63%)
3 (88%)
4
5
Unique indels
total count
0
200
400
600
800
1000
+1 bp
+2 bp
+3 bp
>+3 bp
-1 bp
-2 bp
-3 bp
>-3 bp
Class
control
treated

b

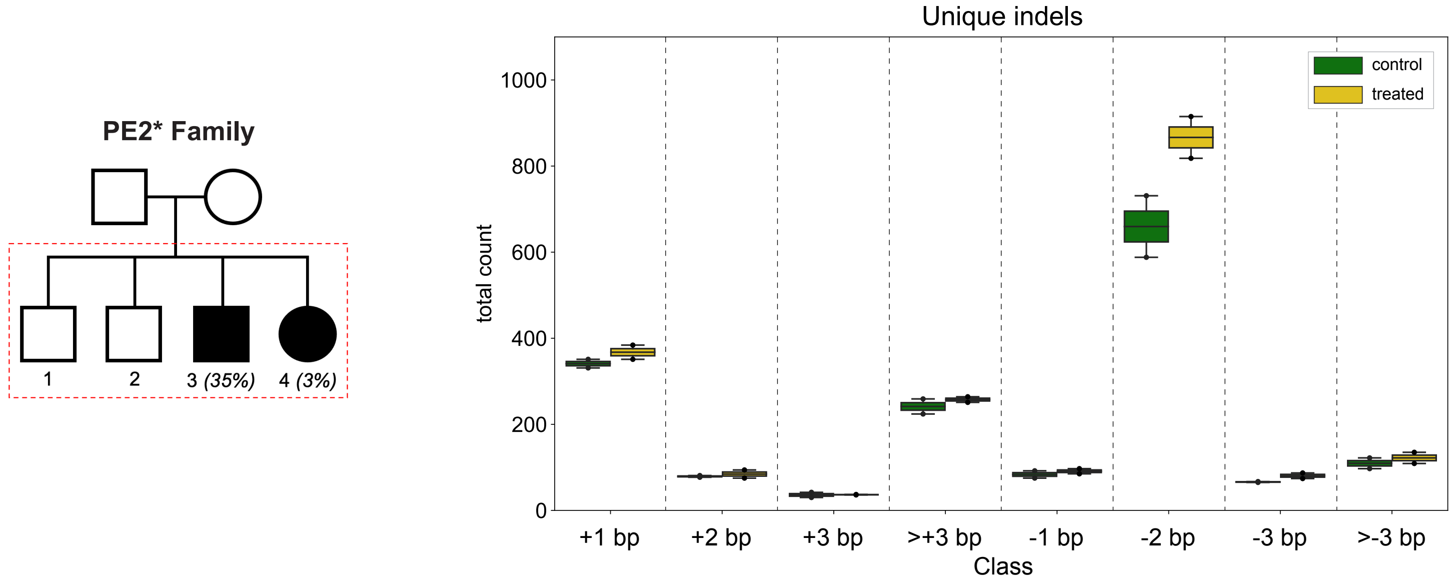
PE2* Family
1
2
3 (35%)
4 (3%)
Unique indels
total count
0
200
400
600
800
1000
+1 bp
+2 bp
+3 bp
>+3 bp
-1 bp
-2 bp
-3 bp
>-3 bp
Class
control
treated

c

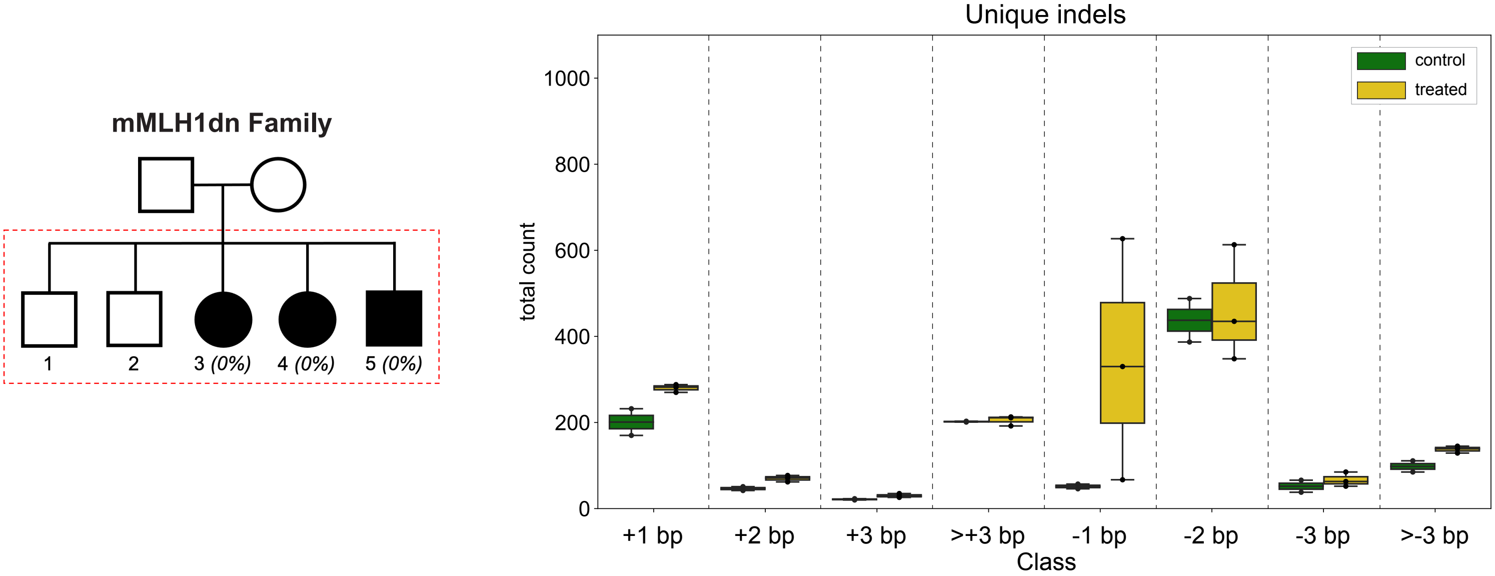
mMLH1dn Family
1
2
3 (0%)
4 (0%)
5 (0%)
Unique indels
total count
0
200
400
600
800
1000
+1 bp
+2 bp
+3 bp
>+3 bp
-1 bp
-2 bp
-3 bp
>-3 bp
Class
control
treated

Supplementary Figure 20. Number of unique indels by type for treated and control samples in mouse families. a, Left) Pedigree of mouse family in which select embryos (black, "treated" group) were microinjected with PE4 components (PE2 editor mRNA, pegRNA, mMLH1dn mRNA) targeting the *Chd2* +5 G>A edit at the two-cell stage. Percentages indicate precise edit efficiency in treated embryos evaluated at E12.5. "Control" group comprised of unshaded mice/embryos from pedigree including both parents and sibling embryos microinjected with PE2 editor mRNA only. Right) Number of unique indels with the indicated classification detected in samples from control (n=4 mice/embryos) and treated (n=3 embryos) groups. **b,** Left) Pedigree of mouse family in which select embryos (black, "treated" group) were microinjected with PE2* components (PEmax editor mRNA, *Chd2* +5 G>A pegRNA) at the two-cell stage. Percentages indicate precise edit efficiency (% of total reads) in treated embryos evaluated at E12.5. "Control" group comprised of unshaded sibling embryos from pedigree which were microinjected with pegRNA only. Right) Number of unique indels with the indicated classification detected in samples from control (n=2 embryos) and treated (n=2 embryos) groups. **c,** Left) Pedigree of mouse family in which select embryos (black, "treated" group) were microinjected with mMLH1dn mRNA and *Chd2* +5 G>A pegRNA (but no editor) at the two-cell stage. Percentages indicate precise edit efficiency (% of total reads) in treated embryos evaluated at E12.5. "Control" group comprised of unshaded sibling embryos from pedigree which were microinjected with pegRNA only. Right) Number of unique indels with the indicated classification detected in samples from control (n=2 embryos) and treated (n=3 embryos) groups. Red dashed box in pedigree diagrams indicate samples subjected to whole genome sequencing. Box plots indicate the median and interquartile range (IQR) of each group with whiskers extending 2.5*IQR past the upper and lower quartiles.

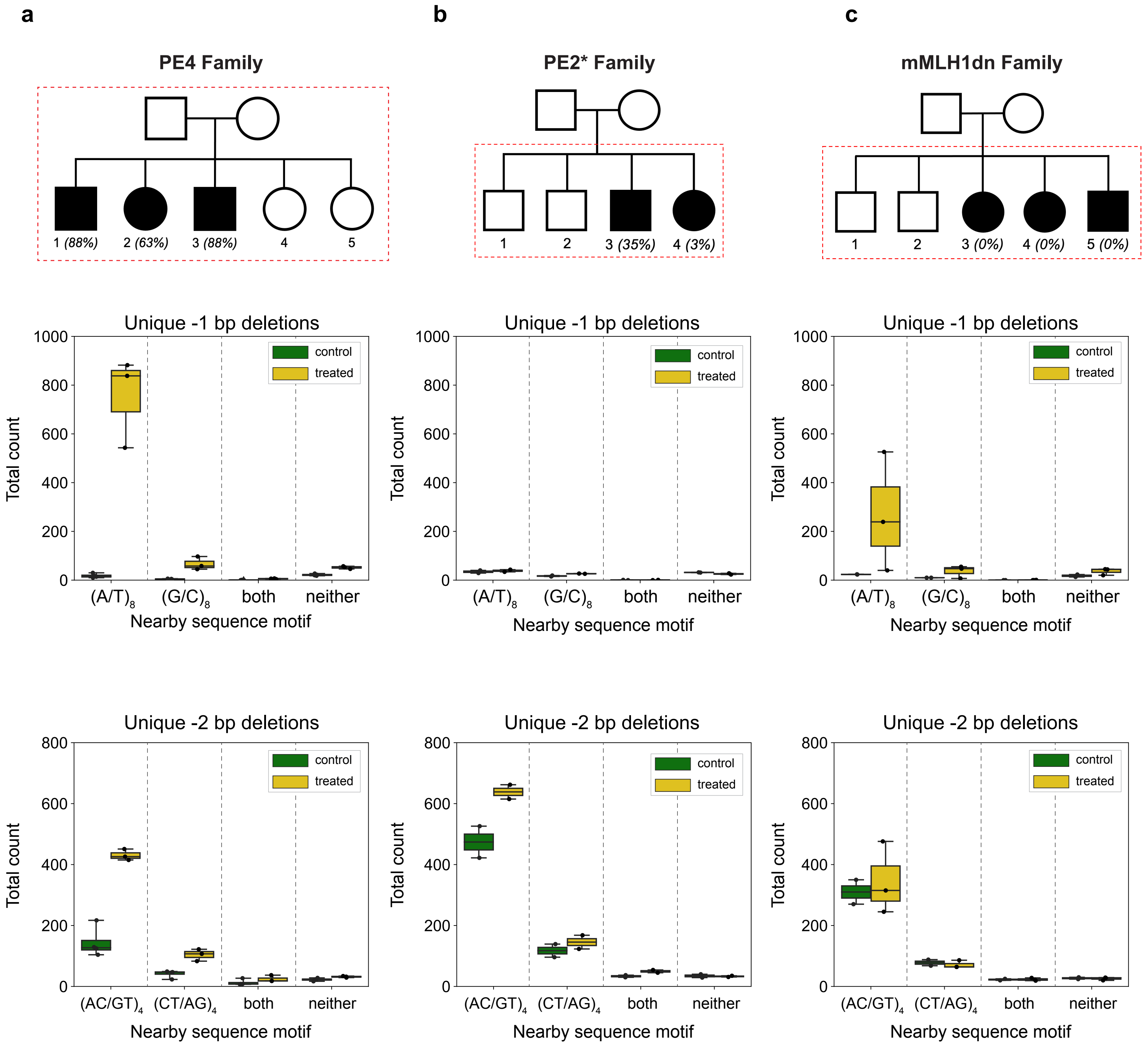
a
PE4 Family
1 (88%)
2 (63%)
3 (88%)
4
5
b
PE2* Family
1
2
3 (35%)
4 (3%)
c
mMLH1dn Family
1
2
3 (0%)
4 (0%)
5 (0%)
Unique -1 bp deletions
Total count
control
treated
(A/T)8
(G/C)8
both
neither
Nearby sequence motif
Unique -2 bp deletions
(AC/GT)4
(CT/AG)4

Supplementary Figure 21. Sequence motifs near unique deletions detected in whole genome sequenced mouse families. a, Top) Pedigree of mouse family in which select embryos (black, "treated" group) were microinjected with PE4 components (PE2 editor mRNA, pegRNA, mMLH1dn mRNA) targeting the *Chd2* +5 G>A edit at the two-cell stage. Percentages indicate precise edit efficiency (% of total reads) in treated embryos evaluated at E12.5. "Control" group comprised of unshaded mice/embryos from pedigree including both parents and sibling embryos microinjected with PE2 editor mRNA only. Middle) Number of unique -1 bp deletions directly adjacent to the indicated sequence motif for individual samples from treated (n=3 embryos) and control (n=4 mice/embryos) groups within the PE4 family. Bottom) Number of unique -2 bp deletions directly adjacent to the indicated sequence motif for individual samples from treated (n=3 embryos) and control (n=4 mice/embryos) groups within the PE4 family. **b,** Top) Pedigree of mouse family in which select embryos (black, "treated" group) were microinjected with PE2* components (PEmax editor mRNA, *Chd2* +5 G>A pegRNA) at the two-cell stage. Percentages indicate precise edit efficiency (% of total reads) in treated embryos evaluated at E12.5. "Control" group comprised of unshaded sibling embryos from pedigree which were microinjected with pegRNA only. Middle) Number of unique -1 bp deletions directly adjacent to the indicated sequence motif for individual samples from treated (n=2 embryos) and control (n=2 embryos) groups within the PE2* family. Bottom) Number of unique -2 bp deletions directly adjacent to the indicated sequence motif for individual samples from treated (n = 2 embryos) and control (n=2 embryos) groups within the PE2* family. **c,** Top) Pedigree of mouse family in which select embryos (black, "treated" group) were microinjected with mMLH1dn mRNA and *Chd2* +5 G>A pegRNA (but no editor) at the two-cell stage. Percentages indicate precise edit efficiency (% of total reads) in treated embryos evaluated at E12.5. "Control" group comprised of unshaded sibling embryos from pedigree which were microinjected with pegRNA only. Middle) Number of unique -1 bp deletions directly adjacent to the indicated sequence motif for individual samples from treated (n=3 embryos) and control (n=2 embryos) groups within the mMLH1dn family. Bottom) Number of unique -2 bp deletions directly adjacent to the indicated sequence motif for individual samples from treated (n=3 embryos) and control (n=2 embryos) groups within the mMLH1dn family. Red dashed box in pedigree diagrams indicate samples subjected to whole genome sequencing. Box plots indicate the median and interquartile range (IQR) of each group with whiskers extending 3.0*IQR past the upper and lower quartiles.

Hoxd13 +6 G>T editing efficiency in 2-3 week old mice after editing embryos at 2-cell stage with PE4 (n=34)

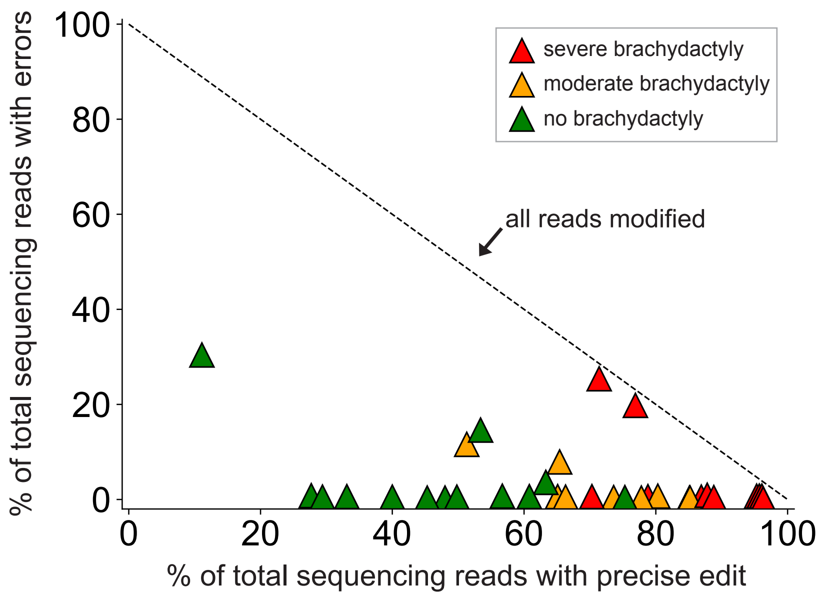

Supplementary Figure 22. *Hoxd13* editing outcome frequencies in PEmbryo edited mice. Percentages of total reads with the precise edit (x-axis) verses errors (y-axis) in 2-3 week old mice developed from embryos microinjected at the two-cell stage with PE4 editing components (PE2 editor mRNA, pegRNA, mMLH1dn mRNA) targeting *Hoxd13* +6 G>T, colored by observed brachydactyly phenotype. Data (Supplementary Table 13) represent the same results depicted in Figure 3a.
