# Supplementary material for: Efficient prime editing in two-cell mouse embryos using PEmbryo
Source: Nat Biotechnol. 2024 Feb 6;42(12):1822–30. doi: 10.1038/s41587-023-02106-x (PMC11631759; doi:10.1038/s41587-023-02106-x)
Supplement: Supplementary file 2 — Reporting Summary [file 41587_2023_2106_MOESM2_ESM.pdf]

nature research

Corresponding author(s): Eszter Posfai
Britt Adamson

Last updated by author(s): Dec 8, 2023

Reporting Summary

Nature Research wishes to improve the reproducibility of the work that we publish. This form provides structure for consistency and transparency in reporting. For further information on Nature Research policies, see our Editorial Policies and the Editorial Policy Checklist.

Statistics

For all statistical analyses, confirm that the following items are present in the figure legend, table legend, main text, or Methods section.

n/a	Confirmed	
☐	☒	The exact sample size (*n*) for each experimental group/condition, given as a discrete number and unit of measurement
☐	☒	A statement on whether measurements were taken from distinct samples or whether the same sample was measured repeatedly
☐	☒	The statistical test(s) used AND whether they are one- or two-sided<br>*Only common tests should be described solely by name; describe more complex techniques in the Methods section.*
☒	☐	A description of all covariates tested
☐	☒	A description of any assumptions or corrections, such as tests of normality and adjustment for multiple comparisons
☐	☒	A full description of the statistical parameters including central tendency (e.g. means) or other basic estimates (e.g. regression coefficient) AND variation (e.g. standard deviation) or associated estimates of uncertainty (e.g. confidence intervals)
☐	☒	For null hypothesis testing, the test statistic (e.g. *F*, *t*, *r*) with confidence intervals, effect sizes, degrees of freedom and *P* value noted<br>*Give P values as exact values whenever suitable.*
☒	☐	For Bayesian analysis, information on the choice of priors and Markov chain Monte Carlo settings
☒	☐	For hierarchical and complex designs, identification of the appropriate level for tests and full reporting of outcomes
☐	☒	Estimates of effect sizes (e.g. Cohen's *d*, Pearson's *r*), indicating how they were calculated

Our web collection on statistics for biologists contains articles on many of the points above.

Software and code

Policy information about availability of computer code

Data collection	Amplicon sequencing was performed on an Illumina MiSeq. Whole genome sequencing was performed on an Illumina NovaSeq 6000. Output base call files (.bcl) were converted to sequence files (.fastq) using bcl2fastq2 Conversion Software v2.20.
Data analysis	Determination of editing outcomes from target site sequencing data was performed with custom python (v3.8.12) scripts as described in Methods. Amplicon reads were aligned to a reference sequence using the pairwise2 module from Biopython (v1.78). Reference sequences were obtained from NCBI using mouse reference genome assembly GRCm39 (GCF_000001635.27). Whole genome sequencing (WGS) reads were aligned using bwa (v0.7.17). WGS analysis was performed with established bioinformatic software packages (Fastq-multx v1.4.2, Trimmomatic v0.39, Samtools v1.15.1, GATK pipeline v4.2.6.1, Picard v2.27.1) as described in Methods. The resulting variant call formatted (.vcf) files were analyzed with custom python (v3.8.12) scripts. All relevant code used for analysis and for reproducing published results are available at https://github.com/badamsonlab/PEmbryo.

For manuscripts utilizing custom algorithms or software that are central to the research but not yet described in published literature, software must be made available to editors and reviewers. We strongly encourage code deposition in a community repository (e.g. GitHub). See the Nature Research guidelines for submitting code & software for further information.

Data

Policy information about availability of data

All manuscripts must include a data availability statement. This statement should provide the following information, where applicable:

- Accession codes, unique identifiers, or web links for publicly available datasets
- A list of figures that have associated raw data
- A description of any restrictions on data availability

Demultiplexed sequence datasets for all samples included in this work are available on NCBI's Sequence Read Archive (SRA) through BioProject accession

PRJNA1040158. Metadata on all collected samples is included in Supplementary Tables 7-14. Variant call formatted (VCF) files from WGS analysis of mouse pedigrees are available at https://github.com/badamsonlab/PEmbryo/. Any additional information is available from the corresponding author upon request.

Field-specific reporting

Please select the one below that is the best fit for your research. If you are not sure, read the appropriate sections before making your selection.

☒ Life sciences ☐ Behavioural & social sciences ☐ Ecological, evolutionary & environmental sciences

For a reference copy of the document with all sections, see nature.com/documents/nr-reporting-summary-flat.pdf

Life sciences study design

All studies must disclose on these points even when the disclosure is negative.

Sample size	No statistical methods were used to pre-determine sample sizes. Group sample sizes were maximized against both biological (e.g. litter size) and technical (e.g. the number of embryos which could be microinjected by a single technician within a reasonable time period) constraints and compare favorably to other publications that evaluate genome editing technologies in mouse embryos. For amplicon sequencing, a target depth of 10,000 reads/sample was chosen in accordance with the standard of the field. For whole genome sequencing, an average genomic coverage of 100x was targeted per mouse to obtain maximal resolution of somatic mutations against technical (Novaseq 6000 produces up to 10e9 paired reads) and economic constraints.
Data exclusions	For the mMLH1dn mouse family subjected to whole genome sequencing to evaluate off-target effects, one control embryo was removed from the dataset after reporting a significantly lower percentage of properly paired reads (93% vs 99% for all other samples). Raw reads from this sample are included in BioProject accession PRJNA1040158.
Replication	Datasets were generated across many individual experiments that each took place over several days. Results reported in the text are therefore an agglomeration of results from multiple experiments. Annotations for each individual embryos, including date of processing, are included in Supplementary Tables 7-14. For repeated treatments groups (i.e., specific prime editor, pegRNA design, target site, edit, and stage of microinjection), editing rates were consistent across separate experiments.
Randomization	Wild type mouse embryos from CD1 or C57Bl/6J backgrounds were used in all experiments. Collected embryos were selected randomly for microinjection of editing components. Illumina indexes were assigned randomly during library preparation. For all other aspects of the study, randomization was not relevant as mouse maintenance and husbandry required careful planning to obtain sufficient number of embryos and final litters for the study and downstream analysis of sequence datasets required mapping results back to specific embryos / treatments to calculate and compare group statistics.
Blinding	For phenotypic assessment of Hoxd13 edited mice, phenotype severity was recorded prior to determination of prime editing efficiency. For preparation and analysis of sequence libraries, investigators were not blinded due to the need to PCR amplify the correct target locus from each embryo and map Illumina indexes back to specific samples / treatments. For determination of editing efficiencies, all sequence datasets were demultiplexed and analyzed identically as detailed in Methods.

Reporting for specific materials, systems and methods

We require information from authors about some types of materials, experimental systems and methods used in many studies. Here, indicate whether each material, system or method listed is relevant to your study. If you are not sure if a list item applies to your research, read the appropriate section before selecting a response.

Materials & experimental systems

n/a	Involved in the study
☒	☐ Antibodies
☒	☐ Eukaryotic cell lines
☒	☐ Palaeontology and archaeology
☐	☒ Animals and other organisms
☒	☐ Human research participants
☒	☐ Clinical data
☒	☐ Dual use research of concern

Methods

n/a	Involved in the study
☒	☐ ChIP-seq
☒	☐ Flow cytometry
☒	☐ MRI-based neuroimaging

Animals and other organisms

Policy information about studies involving animals; ARRIVE guidelines recommended for reporting animal research

Laboratory animals	Wild type mice from CD1 and C57Bl/6J backgrounds were used in all experiments. Males were between 8-16 weeks of age, females were 4-6 weeks old. Mice were subjected to a daily light cycle of 14 hours, with an ambient temperature of 21 deg C and average ambient humidity of 48%.

Wild animals	This study did not involve wild animals.
Field-collected samples	This study did not involve field-collected samples.
Ethics oversight	Mice were housed in an AAALAC-accredited facility following the Guide for the Care and Use of Laboratory Animals. Animal maintenance and husbandry followed the laboratory Animal Welfare Act. Princeton University's Institutional Animal Care and Use Committee (IACUC) approved all animal procedures (protocol number 2133).

Note that full information on the approval of the study protocol must also be provided in the manuscript.
